# Atractylenolide-1 Targets FLT3 to Regulate PI3K/AKT/HIF1-α Pathway to Inhibit Osteogenic Differentiation of Human Valve Interstitial Cells

**DOI:** 10.3389/fphar.2022.899775

**Published:** 2022-04-25

**Authors:** Jie Wang, Penghua Zhang, Jing Zhang, Zhaohui Ma, Xingqin Tian, Yan Liu, Guanghui Lv, Linghang Qu

**Affiliations:** ^1^ Department of Pharmacy, Taihe Hospital, Hubei University of Medicine, Shiyan, China; ^2^ Department of Combine Traditional Chinese and Western Medicine, Taihe Hospital, Hubei University of Medicine, Shiyan, China; ^3^ Children’s Medical Center, Taihe Hospital, Hubei University of Medicine, Shiyan, China; ^4^ College of Pharmacy, Hubei University of Chinese Medicine, Wuhan, China

**Keywords:** Atractylenolide-1, calcification, PI3K, FLT3, HIF1-α

## Abstract

Atractylenolide-1 (AT-1), a natural active ingredient extracted from *Atractylodes macrocephal*a, was reported to have good anti-fibrotic and anti-inflammatory effects. Osteogenic changes induced by the inflammation of valve interstitial cells (VICs) play a role in the development of calcified aortic valve disease (CAVD). This study aimed to investigate the anti-osteogenic effects of AT-1 in human VICs. Human VICs were exposed to osteogenic induction medium (OM) containing AT-1 to analyze cell viability, as well as protein and osteogenic gene expression. Anti-calcification tests were also performed. mRNA transcriptome sequencing was performed to identify differential genes and pathways regulated by AT-1. Western blotting was used to verify the enrichment pathway, protein-protein interaction (PPI) analysis was conducted to identify drug targets. Finally, molecular docking and inhibitors are used to verify the drug targets. Treatment of VICs with 20 μM AT-1 resulted in no significant cytotoxicity. The addition of AT-1 to OM prevented the accumulation of calcified nodules, and decreases in the level of (Alkaline Phosphatase) ALP and RUNX2 gene and protein expression were observed. Atractylenolide-1 can target FLT3 protein and inhibit the phosphorylation of FLT3, thereby blocking PI3K/AKT pathway activation, reducing the production of Hypoxia inducible factor(HIF)1-α, and inhibiting the osteogenic differentiation of VICs. These results suggest AT-1 as a potential drug for treating calcified aortic valve disease.

## Graphical Abstract

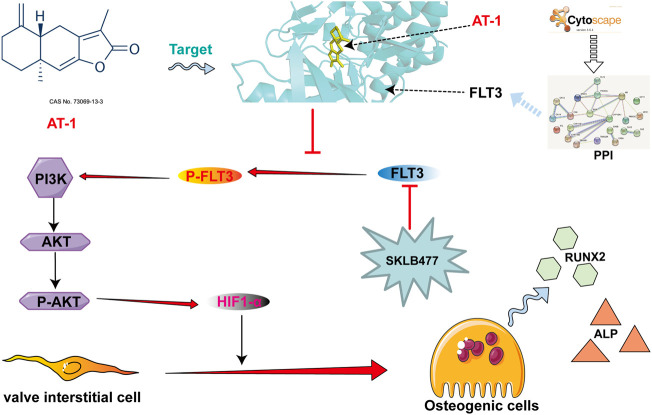

## Introduction

Calcified aortic valve disease (CAVD) is one of the most common heart valve diseases worldwide (Liu et al., 2020). About 1.6 million CAVD patients are predicted worldwide by 2017. CAVD leads to heart failure, resulting in tens of thousands of deaths each year (Yadgir et al., 2020). The lesion is characterized by fibrosis, as well as thickening and calcification of the aortic valve (Katsi et al., 2021). CAVD refers to chronic degeneration, fibrosis, and calcium deposition of the fibrous support of the heart valve, which can lead to thickening and hardening of the heart valve, thereby inducing heart valve stenosis, heart failure, or death (K. Xu et al., 2020). The factors influencing its incidence are complex, and the incidence increases with age, seriously threatening human health and life. The early symptoms of CAVD are the destruction of the endothelial layer on the aortic side of the valve leaflets. Stromal changes occur with age, resulting in thickening of the valve, and then, the leaflets are infiltrated by immune cells (Mohler, 2004). At the same time, angiogenesis is accompanied by the accumulation of cellular debris including lipids ad proteoglycans, which deforms the valve leaflets, calcifies the valve matrix, and stiffens the aortic valve, resulting in aortic valve stenosis and obstruction of the blood flow (Chen & Simmons, 2011). It is estimated that approximately 13% of calcified aortic valves at the time of surgery contain true bone as well as osteoblasts and osteoclasts (Mohler, 2004). The aortic valve consists of three small collagen leaflets, each of which is filled with valve interstitial cells (VICs) (Misfeld & Sievers, 2007). The osteoblastic differentiation of VICs is similar to physiological osteogenesis (Miller et al., 2011). Previous studies showed that inhibiting the osteogenic differentiation of VICs caused by inflammation could be the focus of CAVD treatment (Wang Y. et al., 2021).

Once valve calcification occurs, the lesion is histologically irreversible. The only clinically effective treatment for cardiac valve calcification is valve replacement (Hutcheson et al., 2014). At present, there is no suitable drug for the clinical treatment of CAVD. Therefore, the search for effective drugs for treating CAVD has attracted more and more attention. Traditional Chinese medicine (TCM) has played an important role in the prevention and treatment of heart-related diseases. Previously, the research group conducted anti-calcification screening of a large number of natural active components of Chinese herbal medicines and found some components that may have a therapeutic effect on CAVD, such as nobiletin (K. Xu et al., 2019), andrographolide (AGP) (Wang, et al., 2021a), and cardamonin (Wang, et al., 2021b). Among them, nobiletin and andrographolide have been patented.

Atractylenolide-1 (AT-1) is a natural product extracted from the TCM *Atractylodes macrocephala* or Atractylodes Rhizoma (Qu, et al., 2022a). Many previous studies reported that AT-1 had good anti-inflammatory and anti-fibrotic activity (Du et al., 2022; Guo et al., 2021; Liu, et al., 2021; H.; Xu et al., 2021). Our previous study also showed that AT-1 had a better therapeutic effect on (Dextran sulfate sodium) DSS-induced colitis (Qu, et al., 2022b). However, the therapeutic effect of AT-1 on CAVD has not been reported. Thus, we investigated the inhibitory effect and potential mechanism of AT-1 on the osteogenic differentiation of VICs.

## Materials and Methods

### Cell Culture and Treatments

Human aortic VICs were isolated from patients undergoing Bentall surgery due to acute type I aortic dissection in Tongji Medical College, Huazhong University of Science and Technology. The patients provided written informed consent (Supplementary Table S1). The cells were separated according to the method in our previous study (K. Xu et al., 2019). After amplification, fourth or fifth-generation cells were used in all experiments. *In vitro*, osteogenic medium(OM) (Cyagen Biosciences, HUXMA-90021) was used to induce the osteogenic differentiation of the VICs. SKLB4771 was purchased from selleck(#S1099,China).

### Cell Viability Testing

VICs were inoculated into 96-well plates, incubated with different concentrations of AT-1 for 48 h, and cell viability was measured using the CCK-8 (GLPBIO,#GK10001) method. VICs were inoculated into 48-well plates, incubated with or without 20 μm AT-1, and photographed under the microscope to record the cell morphology and density at the same time every day (Liu et al., 2020).

### Calcification Analysis

VICs were inoculated into 24-well plates and divided into five groups: the control group, OM-stimulated group, OM + AT-1 10 μm group, OM + AT-1 20 μm group, and the OM + AGP group. The OM medium and therapeutic drugs were changed every three days. After 18 days of continuous culture, the calcified crystals were stained with Alizarin Red. Then, 10% aqueous solution of cetyl-pyridinium chloride solution was added to each well to dissolve the Alizarin Red dye. The amount of Alizarin Red dye was quantified by the absorbance at 550 (Liu et al., 2020).

### RT-PCR

VICs were inoculated into 6-well plates, osteogenic differentiation was induced using OM medium, and the cells were treated with different concentrations of AT-1 and positive control drugs. After 48 h, total RNA was isolated from the cells using Trizol reagent.RNA was reverse transcribed into cDNA using a HiScript® II Q RT SuperMix (R223-01) (Vazyme Biotech Co., Ltd., Nanjing, China), and amplified on a PCR instrument, using 2X Universal SYBR Green Fast qPCR Mix (RK21203) (ABclonal Technology, Wuhan, China) for quantification. All primers were as in previous studies and synthesized by Tsingke Biotechnology Co., Ltd. Supplementary Table S2 shows the primer sequences(Liu et al., 2020). The 2^−∆∆CT^ method was used to analyze the final data (Qu et al., 2021).

### Western Blotting

VICs were inoculated into a 6-cm diameter dish and osteogenic differentiation was induced using OM medium. The cells were treated with different concentrations of AT-1 and positive control drugs. Total cellular proteins were collected in RIPA lysate. The protein concentration in the cells was measured using the enhanced BCA protein detection kit. Equal amounts of protein underwent 10% sodium dodecyl sulfate-polyacrylamide gel electrophoresis (SDS-PAGE) and were transferred to a polyvinylidene fluoride (PVDF) membrane overnight. The membranes were then blocked in 5% skimmed milk powder for one hour and incubated with primary antibodies against β-actin (Solarbio, #K2000558M), RUNX2 (Abcam, ab236639), ALP (Zenbio,#381009), HIF1-α (Abcam, ab179483, FLT3 (Cell Signaling Technology, #3462s), or P-FLT3 (Cell Signaling Technology, #60413) overnight at 4°C. Then, the membranes were washed with Tris-buffered saline with Tween 20 (TBST) at room temperature, followed by incubation with a secondary antibody for one hour. Enhanced chemiluminescence reagents were added, the protein bands were visualized using the Tanon-5200 system. ImageJ software was used to quantify the band density.

### Colorimetric Detection of ALP Activity

After the VICs were treated with OM medium and drugs, the culture medium was aspirated, and the cells were washed once with phosphate-buffered saline (PBS). The cells were harvested by scraping them into PBS and collected by centrifugation at 1000 × g at 4°C for 10 min. Homogenization medium was added for mechanical homogenization at 4°C, centrifuged at 10,000 × g for 10 min, and the supernatant was collected. ALP activity was assayed according to the kit manufacturer, and the absorbance at 520 nm was measured with a UV spectrophotometer.

### mRNA Profile Detection

Changes in cell mRNA profiles in the different treatment groups were analyzed by RNA-sequencing (RNA-seq). Isolated RNA samples were sent to BGI Co., LTD. in Wuhan for mRNA-seq using BGISEQ-500. Differentially expressed gene (DEG), Kyoto Encyclopedia of Genes and Genomes (KEGG), and Gene Ontology (GO) pathway enrichment analyses were performed using the online platform “Dr. Tom 2.0” designed by BGI Tech Co., LTD.

### Immunofluorescence

OM stimulated VIC cells were administered with or without AT-1. After 48 h incubation, the medium was discarded and washed three times with PBS. Cells were fixed in 4% paraformaldehyde for 20 min at 25°C. Washed 3 times with PBS. Add 0.1% Triton X-100 and permeabilize for 20 min. Washed 3 times with PBS. Block with goat serum for 20 min. Primary antibody to RUNX2 was added and incubated overnight at 4 degrees. Washed 3 times with PBS. Add secondary antibody and incubate for 1 h. Washed 3 times with PBS. Slides were made and an anti-fluorescence quencher containing DAPI was added. The results were observed under a fluorescence microscope.

### Protein-Protein Interaction Network

The String plugin in the Cytoscape software was installedAfter the installation was complete, the proteins that required protein interaction were imported. The cutoff value was set to 0.7. After the protein was imported, a preliminary network diagram was obtained.

### Statistical Analysis

Statistical analysis was performed using GraphPad v8.0. Shapiro - Wilk test was applied for Normality tests and variance homogeneity. Data with normal distribution were analyzed by one-way analysis of variance (ANOVA), results are shown as mean ± standard deviation (SD). While those non-normally distributed or with uneven variance were analyzed by Kruskal-Wallis, results are shown as median (quartile). All data except RNA sequencing were analyzed by the above method. A value of *p* < 0.05 was considered statistically significant.

## Results

### Viability and Calcification of VICs Treated With AT-1

**FIGURE 1:**
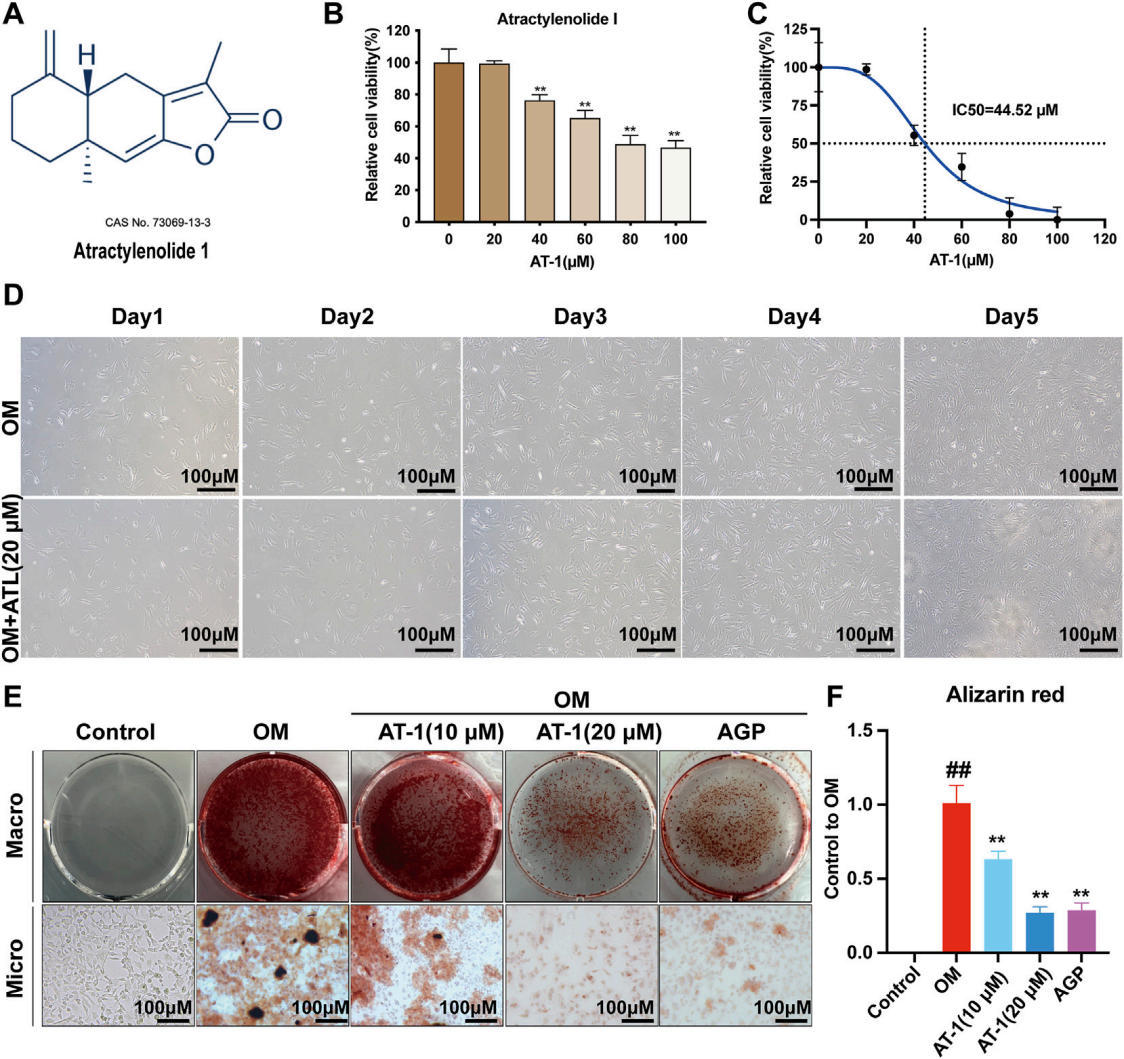
Viability and calcification of VICs treated with AT-1. **(A)** Chemical structure of atractylenolide-1. **(B)** The CCK8 method was used to analyze the effect of treatment with different atractylenolide-1 concentrations on VICs. **(C)** IC50 of atractylenolide-1 in VIC cells. **(D)** Effect of no AT-1 and 20 μM AT-1 on the proliferation of OM-induced VIC cells in 5 days. **(E)** Cells with different treatments (control (normal culture medium), OM (osteogenic medium), OM+10 μM AT-1 (osteoblastic medium plus10 μM AT-1 treatment), OM+20 μM AT-1 (osteoblastic medium plus 20 μM AT-1 treatment), and OM + AGP (osteoblastic medium plus 10 μM andrographolide) were stained with Alizarin Red S. **(F)** Alizarin Red staining statistics. *p < 0.05, **p < 0.01 indicated a significant difference compared to the control group and #*p* < 0.05, ##*p* < 0.01 indicated a significant difference compared to the OM group, *n* = 3.

Different concentrations of AT-1 were incubated with VICs for 48 h. The results showed that 0–20 μM AT-1 was not cytotoxic to the VICs (Figures 1A,B). Treatment with AT-1 at concentrations of over 40 μM obviously inhibited the proliferation of VICs, with an IC50 of 44.52 μM (Figure 1C). We further investigated state of cells treated with 20 μM AT-1 co-incubated with OM-induced VICs for 5 days. The results showed that 20 μM AT-1 did not affect the normal growth and proliferation of OM-induced VICs (Figure 1D). VICs were induced in OM medium for 18 days and treated with different concentrations of AT-1. AGP was used as the positive control. The Alizarin Red results showed that 10 and 20 μM AT-1 significantly inhibited the calcification of VICs (Figures 1E,F).

### Effect of AT-1 on OM-Induced Calcific-Related Gene and Protein Expression in VICs

**FIGURE 2:**
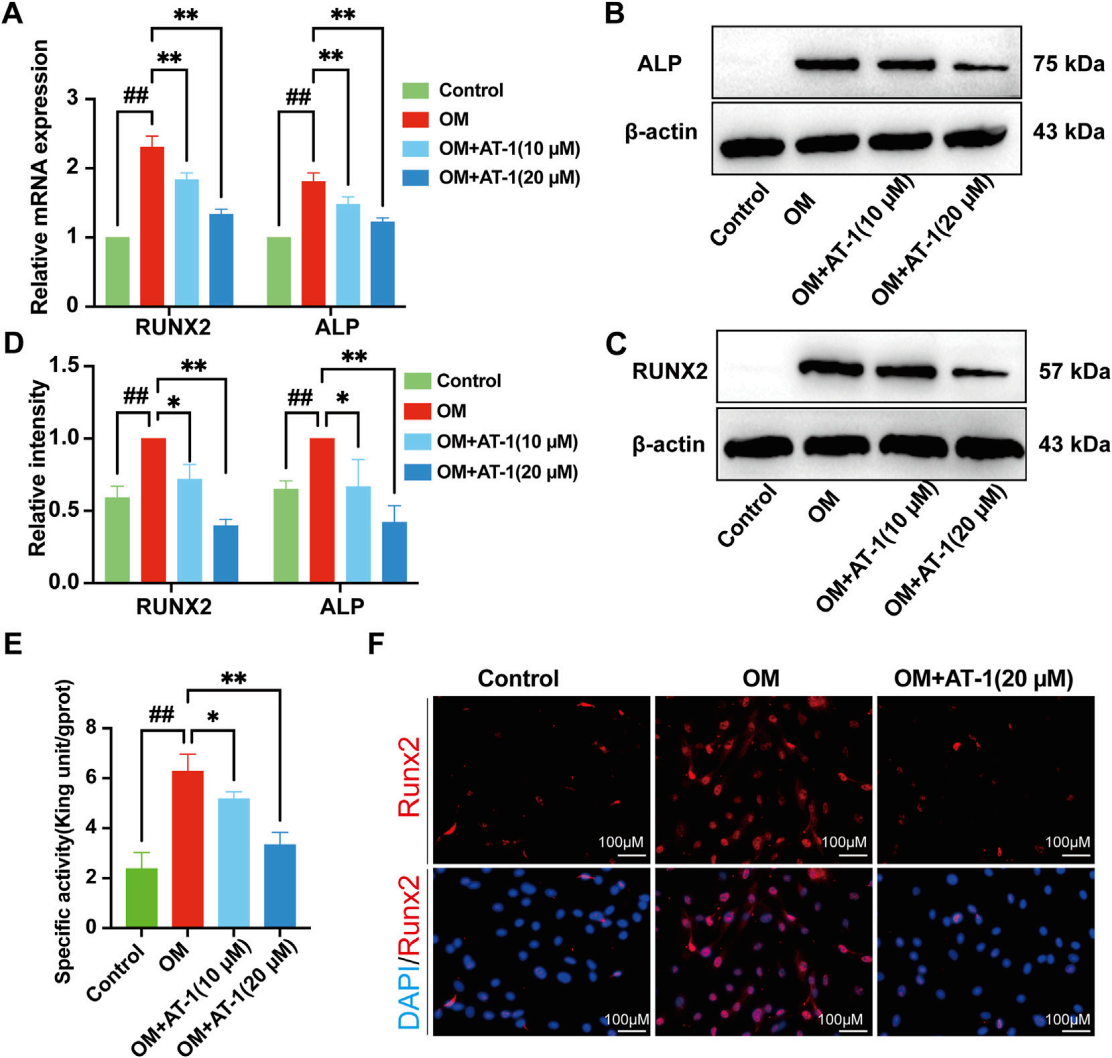
Effect of AT-1 on OM-induced calcific-related gene and protein expression in VICs. **(A)** VICs were stimulated with OM and then treated or not with AT-1 for 48 h. The mRNA RUNX2 and ALP expression levels were analyzed by quantitative real-time polymerase chain reaction (qRT-PCR). **(B, C)** VICs were stimulated with OM and then treated or not with AT-1 and AGP for 48 h and the expression levels of RUNX2 and ALP protein were detected by Western blotting. **(D)** Statistical analysis of Runx2 and ALP protein expression. **(E)** The ALP activity of the VICs was measured using the ALP biochemical kit. **(F)** The expression of RUNX2 in VICs was evaluated at 48 h by immunofluorescence. *p < 0.05, **p < 0.01 indicated a significant difference compared to the control group and #*p* < 0.05, ##*p* < 0.01 indicated a significant difference compared to the OM group, *n* = 3.

The expression of the osteogenic differentiation-related genes RUNX2 and ALP was analyzed in VICs cultured in OM with or without AT-1 for 48 h by RT-PCR. OM significantly upregulated ALP and RUNX2 expression (*p < 0.05) compared to the control group, whereas ALP and RUNX2 were significantly downregulated in VICs treated with different concentrations of AT-1 in OM medium (*p* < 0.05) (Figure 2A).We detected the protein levels of RUNX2 and ALP in VICs stimulated in OM for 48 h by Western blots. AT-1 significantly inhibited increases in the protein expression of RUNX2 and ALP (*p* < 0.05) (Figures 2B–D). We used the ALP biochemical kit to measure ALP activity in the cells, which showed that the ALP activity of VICs stimulated by OM increased, and AT-1 inhibited the ALP activity (Figure 2E). We also detected the protein expression of RUNX2 in VICs induced by OM medium and treated with 20 μM AT-1 using immunofluorescence. The results showed that 20 μM AT-1 could significantly inhibit the increase in RUNX2 proteins induced by OM medium (Figure 2F).

### Changes in Gene Expression Under Different Treatments Analyzed by mRNA-Seq

**FIGURE 3:**
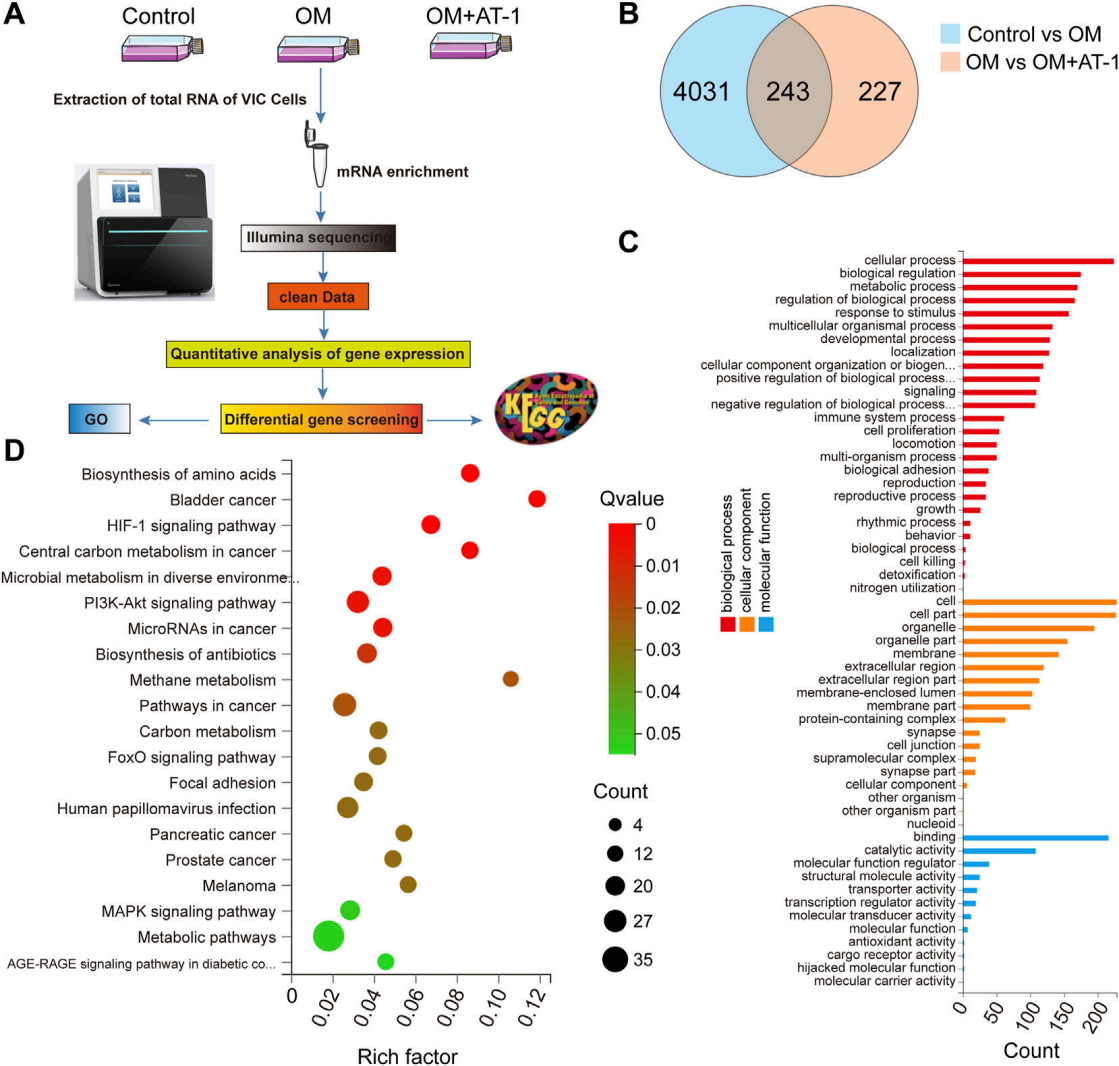
Changes in gene expression under different treatments analyzed by mRNA-seq. **(A)** Schematic diagram of the mRNA transcriptome sequencing process. **(B)** Venn interaction of DEGs of the Control versus the OM group, and the OM group versus the OM + AT-1 group. **(C)** GO enrichment of the commonly detected DEGs, which included molecular function, cellular components, and biological processes. **(D)** KEGG pathway enrichment bubble map, where a larger *p*-value (-log10) indicates a higher degree of enrichment.

Investigate the mechanism by which AT-1 inhibited the osteogenic differentiation of valve cells, we collected mRNA from the normal group, OM-stimulated group, and the OM-stimulated group plus AT-1 group, and performed mRNA sequencing analysis, then GO enrichment and KEGG enrichment analyses (Figure 3A). The Venn diagram showed that there were 243 genes altered by AT-1 (Figure 3B). GO enrichment analysis showed that AT-1 mainly affected the biological processes of the cells (Figure 3C). KEGG enrichment analysis showed that AT-1 mainly regulated the PI3K-AKT pathway and the HIF1-α pathway (Figure 3D).

### Effect of AT-1 on the PI3K/AKT/HIF1-α Pathway and Investigation of its Upstream Targets

**FIGURE 4:**
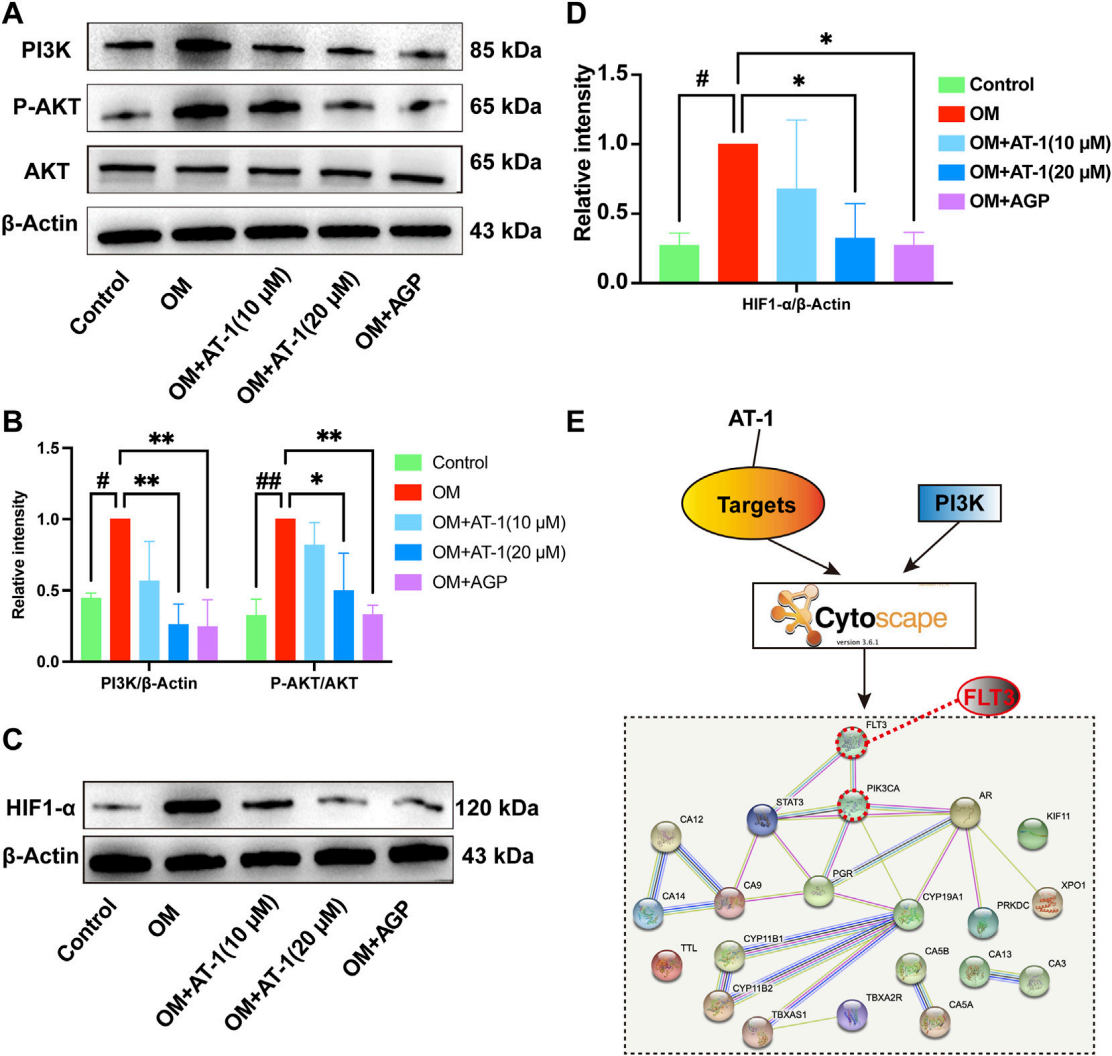
The effect of AT-1 on the PI3K/AKT/HIF1-α pathway and investigations into its upstream targets. **(A)** Effect of different AT-1 and AGP concentrations on the expression of PI3K, AKT, and P-AKT proteins **(B)** Statistical analysis of PI3K/β-actin and P-AKT/AKT. **(C)** Effect of different AT-1 and AGP concentrations on the expression of HIF1-α protein. **(D)** Statistical analysis of HIF1-α/β-actin. **(E)** The predicted AT-1 targets and PI3K pathway proteins were subjected to PPI network association analysis to identify the upstream targets of AT-1 regulating the PI3K pathway. *p < 0.05, **p < 0.01 indicated a significant difference compared to the control group and #*p* < 0.05, ##*p* < 0.01 indicated a significant difference compared to the OM group, *n* = 3.

To verify the mRNA transcriptome sequencing results, we collected proteins from the normal group, OM-stimulated group, OM-stimulated group plus different concentrations of AT-1, and the AGP administration group and performed Western blot analysis. The results showed that AT-1 dose-dependently inhibited the expression of PI3K protein, P-AKT protein, and HIF1-α protein (Figures 4A–D). To further clarify how AT-1 regulated the PI3K/AKT/HIF1-α pathway, we predicted the targets of AT-1 through the SwissTarget website, and then imported all the predicted targets and PI3K pathway proteins into Cytoscape software for PPI analysis. The results suggested that AT-1 may regulate the PI3K/AKT pathway by targeting FLT3 (Figure 4E).

### AT-1 Inhibits the Phosphorylation of FLT3 and Inhibits the Activation of PI3K/AKT/HIF1-α in VICs

**FIGURE 5:**
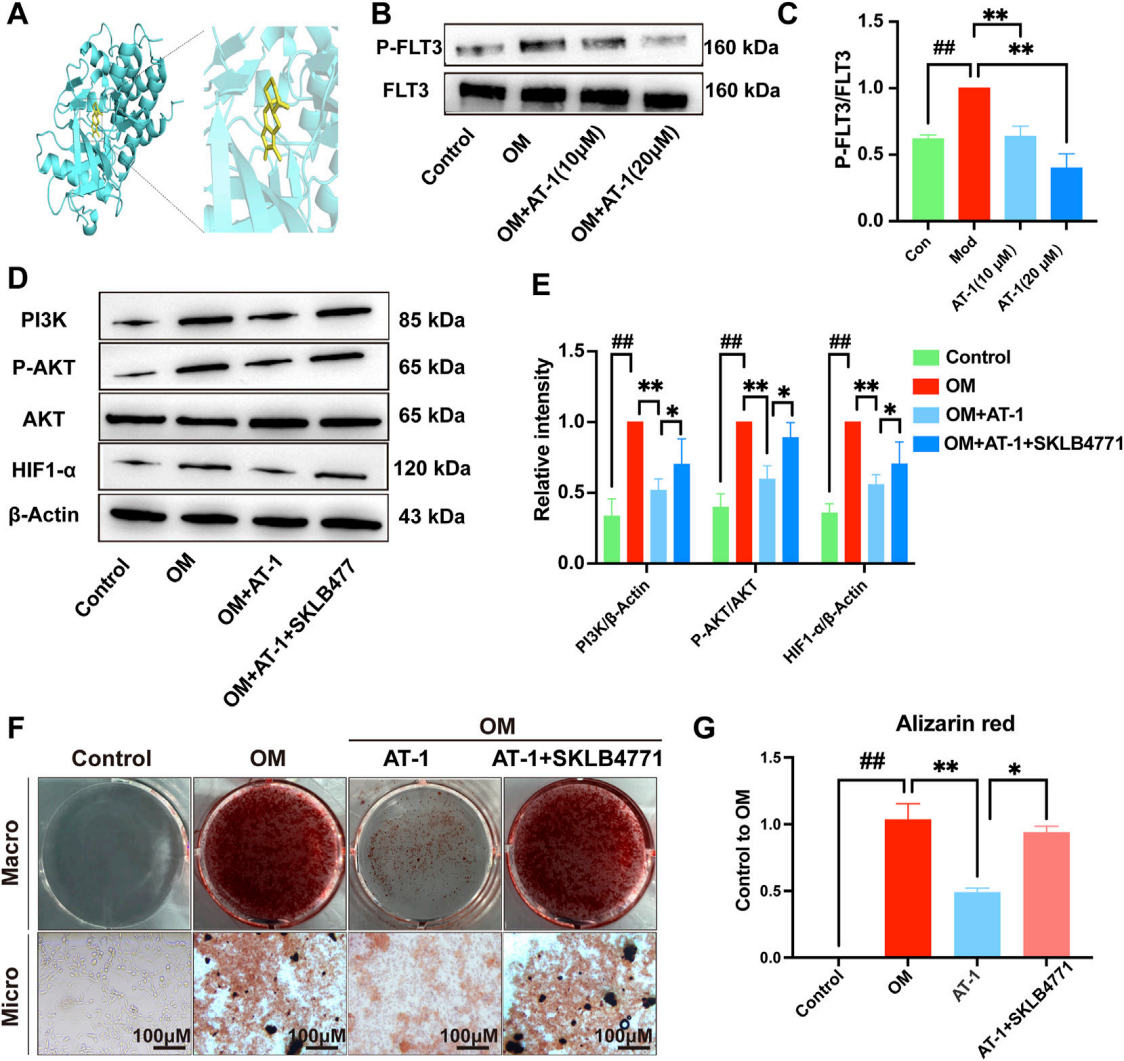
AT-1 inhibits the phosphorylation of FLT3 and the activation of PI3K/AKT/HIF1-α, thereby inhibiting the osteogenic differentiation of VICs. **(A)** Molecular docking technology was used to detect the binding ability of AT-1 to FLT3 protein. **(B)** Effect of different AT-1 concentrations on the level of phosphorylated FLT3 protein in OM-induced VIC cells. **(C)** Statistical analysis of P-FLT3/FLT3. **(D)** Effect of AT-1 treatment with or without FLT3 inhibitor on the regulation of PI3K/AKT/HIF1-α in OM-induced VICs. **(E)** Statistical analysis of PI3K/β-actin, P-AKT/AKT, and HIF1-α/β-actin. **(F)** Cells with different treatments (control, OM, OM+20 μM AT-1, and OM+20 μM AT-1+10 nM SKLB4771) were stained with Alizarin Red S. **(G)** Alizarin Red staining statistics. *p < 0.05, **p < 0.01 indicated a significant difference compared to the control group and #*p* < 0.05, ##*p* < 0.01 indicated a significant difference compared to the OM group, *n* = 3.

To verify the PPI analysis results, we used molecular docking technology to calculate the binding energy between AT-1 and FLT3 protein. The results showed that the binding energy of AT-1 and FLT3 protein was as high as −8.4 kcal/mol, indicating strong binding ability (Figure 5A). We compared the expression levels of FLT3 and its phosphorylation in the normal group, OM-stimulated group, and OM-stimulated group plus different doses of AT-1. The results showed that AT-1 dose-dependently inhibited the expression of phosphorylated FLT3 protein **(** Figures 5B,C). To further verify whether AT-1 targeted FLT3 to regulate the PI3K/AKT/HIF1-α pathway, we used the FLT3 inhibitor SKLB477. The addition of SKLB477 prevented the AT-1 inhibition of the PI3K/AKT/HIF1-α pathway (Figures 5D,E). The Alizarin Red results showed that the inhibitory effect of AT-1 on the osteogenic differentiation of valve cells was reversed after adding SKLB477 (Figures 5F,G).

## Discussion

Our results show that AT-1 targeted the FLT3 protein and inhibited the phosphorylation of FLT3, blocking PI3K/AKT pathway activation and reducing the production of HIF1-α, thereby inhibiting the osteogenic differentiation of VICs.

Overactivation or alterations in the PI3K/AKT pathway, which regulates cellular processes including metabolism, proliferation, growth, survival, angiogenesis, and metastasis, are seen in many cancer types. The PI3K/AKT/mTOR pathway is regulated by multiple upstream signaling proteins, which affect many downstream effectors by cooperating with various compensatory signaling pathways (Ersahin, Tuncbag, & Cetin-Atalay, 2015). Many studies have shown that the activation of the PI3K/AKT pathway was involved in promoting the osteogenic differentiation of valve cells and that inhibiting the activation of the PI3K/AKT pathway was an important strategy to prevent valve cell calcification (Zhu et al., 2019). For example, curcumin was shown to inhibit the osteogenic differentiation of valve cells by inhibiting the PI3K/AKT pathway (Zhou et al., 2020). Studies showed that caffeic acid improved calcification in human aortic valve interstitial cells by inhibiting the activation of the AKT/NF-κB/NLRP3 inflammasome pathway (Liu et al., 2020). This study found that AT-1 inhibited PI3K protein and P-AKT protein overexpression in VICs.

HIF1-α is a key transcription factor in response to hypoxia. HIF-1 was shown to be a key regulator of vascular calcification (Patten et al., 2010). HIF-1 *α* expression was significantly increased in various ischemic organs and tissues such as the retina, nervous system, and myocardium. The protective effect of HIF-1 has also been widely reported in various ischemic models (Yao et al., 2016). Previous studies have also shown that HIF-1 *α* was regulated by the PI3K/Akt/FRAP and PI3K/Akt/mTOR signaling pathways. HIF-1α and P-Akt protein levels were increased in response to hypoxia in human mesenchymal stem cells. Additionally, peak p-Akt expression occurred earlier than that of HIF-1 *α*. The PI3K inhibitor LY294002 and the PI3K/mTOR inhibitor NVP-BEZ235 inhibited ischemia-induced p-Akt and HIF-1α activation. The Akt inhibitor wortmannin was also shown to inhibit HIF-1α (Zhong et al., 2000) (Li, Qu, Mao, Xiong, & Mu, 2008). Our study showed that HIF1-α was highly expressed in the osteogenic differentiation process of VICs, and AT-1 significantly inhibited HIF1-α expression.

FLT3 (Fms-like tyrosine kinase, FMS-like tyrosine kinase 3) is a member of the type III receptor tyrosine kinase (RTK III) family (Chougule et al., 2016). In recent years, many large sample studies have reported the important pathological role played by the activation of FLT3 mutation in the occurrence and progression of acute myelocytic leukemia(AML). FLT3 receptor tyrosine kinase is activated in the plasma membrane to transduce RAS/MAPK and PI3K/Akt signaling (Lv et al., 2021). Our study showed that AT-1 could target FLT3 protein and inhibit the phosphorylation of FLT3 protein, thus inhibiting PI3K/Akt/HIF1 pathway activation.

In conclusion, our study found for the first time that AT-1 could inhibit the osteogenic differentiation of VICs, and preliminarily revealed that its mechanism may be related to targeting FLT3 and inhibiting PI3K/Akt/HIF1 pathway activation, providing a new strategy for treating aortic calcification. However, our research still had some limitations. Due to the lack of appropriate animal models and the long time required for the existing animal models, we could not verify the efficacy of AT-1 in the treatment of valve calcification in the appropriate animal models. Next, we will explore its efficacy and mechanism in animal models.

## Data Availability

The datasets presented in this study can be found in online repositories. The names of the repository/repositories and accession number(s) can be found below: https://www.ncbi.nlm.nih.gov/; PRJNA817634.
