# Supplementary material for: Atractylenolide-1 Targets FLT3 to Regulate PI3K/AKT/HIF1-α Pathway to Inhibit Osteogenic Differentiation of Human Valve Interstitial Cells
Source: Front Pharmacol. 2022 Apr 25;13:899775. doi: 10.3389/fphar.2022.899775 (PMC9097085; doi:10.3389/fphar.2022.899775)
Supplement: Supplementary file 1 [file Table1.DOCX]

**Supplementary Table 1. Human aortic valve samples**

| **Admission number** | **Sex** | **Age** |
| --- | --- | --- |
| 1931505 | F | 63 |

**Supplementary Table 2. Primer sequences**

| **Gene name** | Primer sequences（5^，^-3^，^） |
| --- | --- |
| **RUNX2(F)** | CCGCCTCAGTGATTTAGGGC |
| **RUNX2(R)** | GGGTCTGTAATCTGACTCTGTCC |
| **ALP(F)** | TACACGGTCCTCCTATACGGAA |
| **ALP(R)** | CTCTCGCTCTCGGTAACATC |
| **β-Actin（F）** | GGATTCCTATGTGGGCGACGA |
| **β-Actin（R）** | GCGTACAGGGATAGCACAGC |
