# Supplementary figures and images for: Atractylenolide-1 Targets FLT3 to Regulate PI3K/AKT/HIF1-α Pathway to Inhibit Osteogenic Differentiation of Human Valve Interstitial Cells
Source: Front Pharmacol. 2022 Apr 25;13:899775. doi: 10.3389/fphar.2022.899775 (PMC9097085; doi:10.3389/fphar.2022.899775)

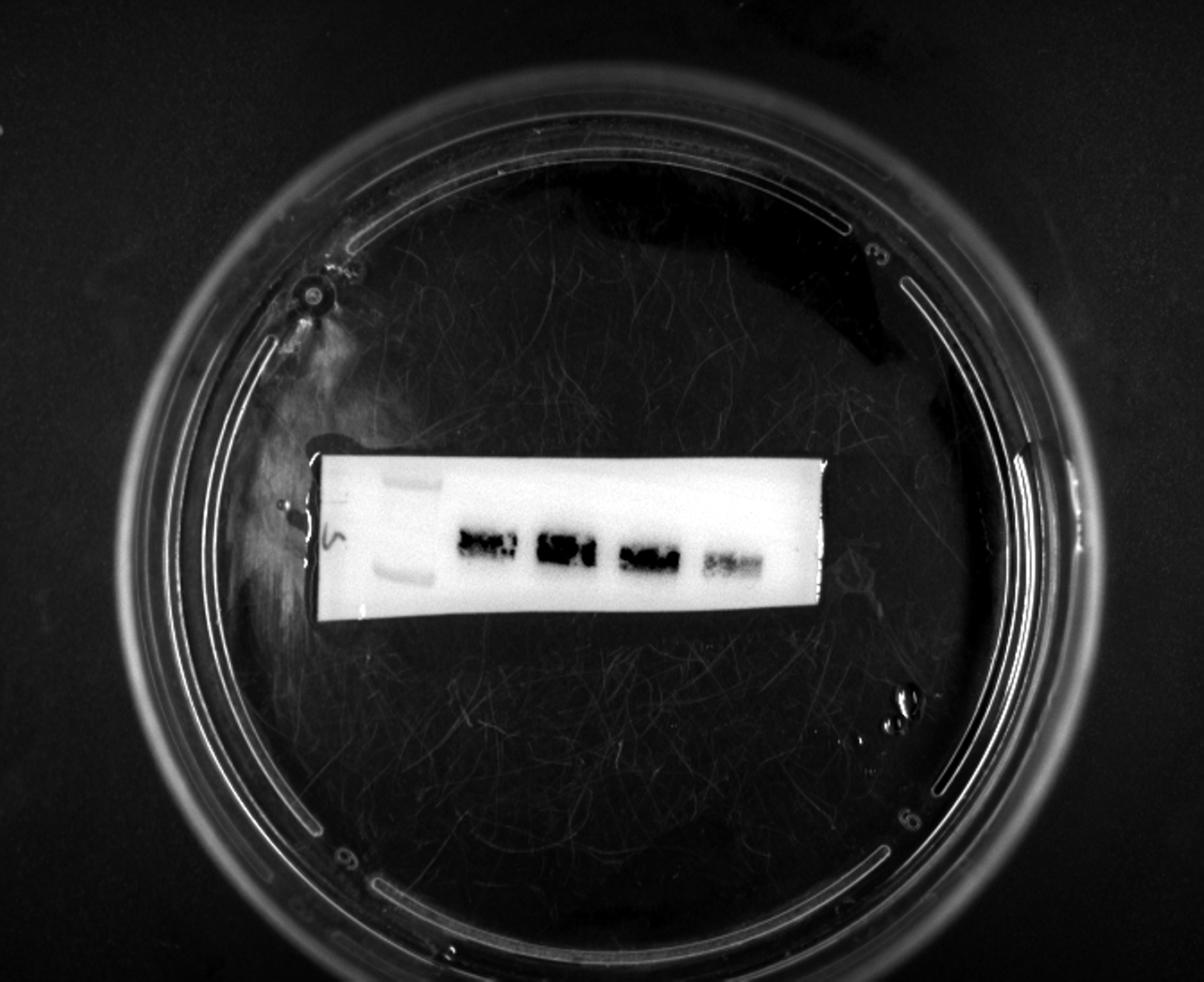

Supplement: Supplementary file 2 [file DataSheet1.ZIP › WB-rawdata/FIG5B/P-FLT3-3.Tif]

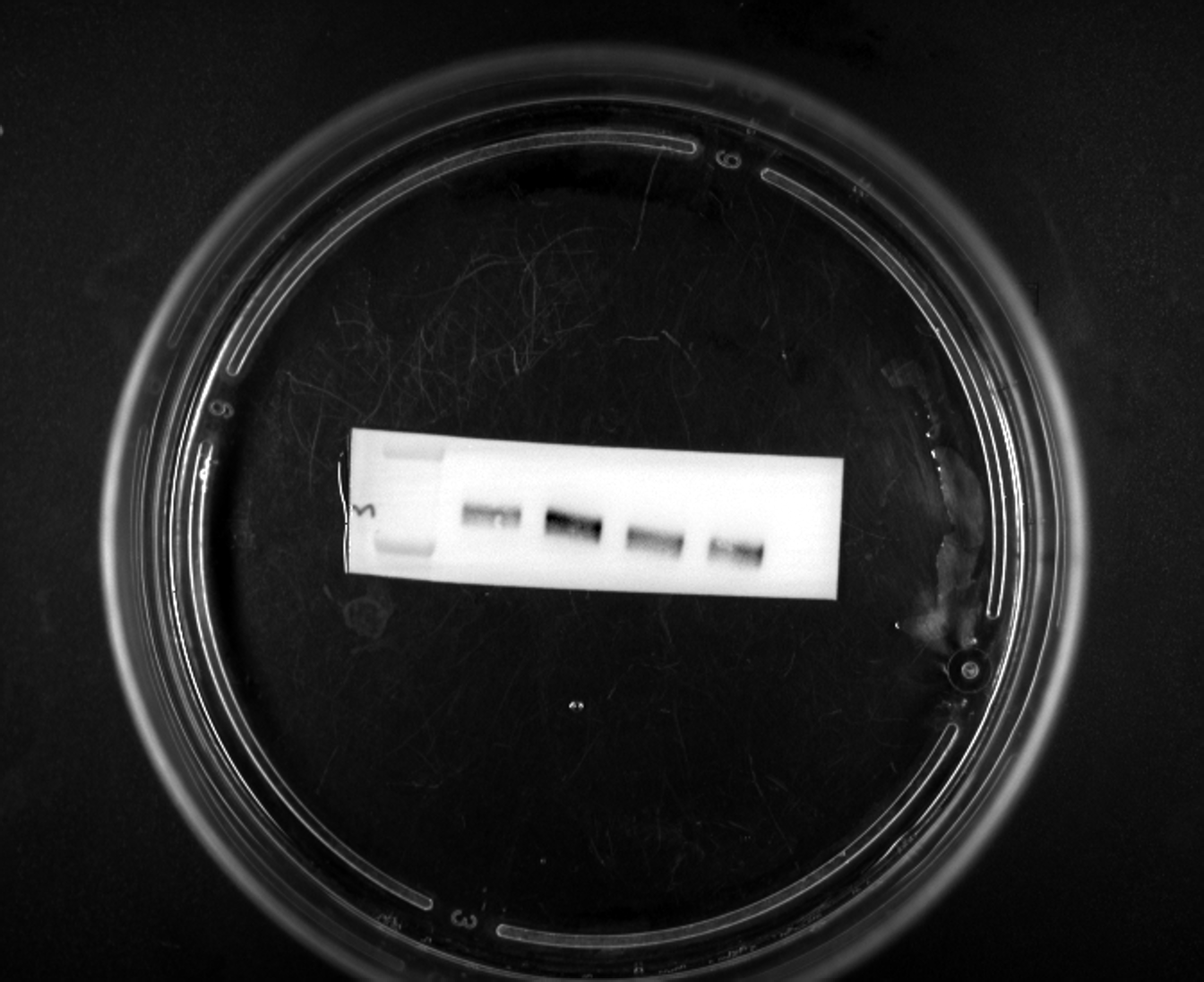

Supplement: Supplementary file 2 [file DataSheet1.ZIP › WB-rawdata/FIG5B/P-FLT3-2.Tif]

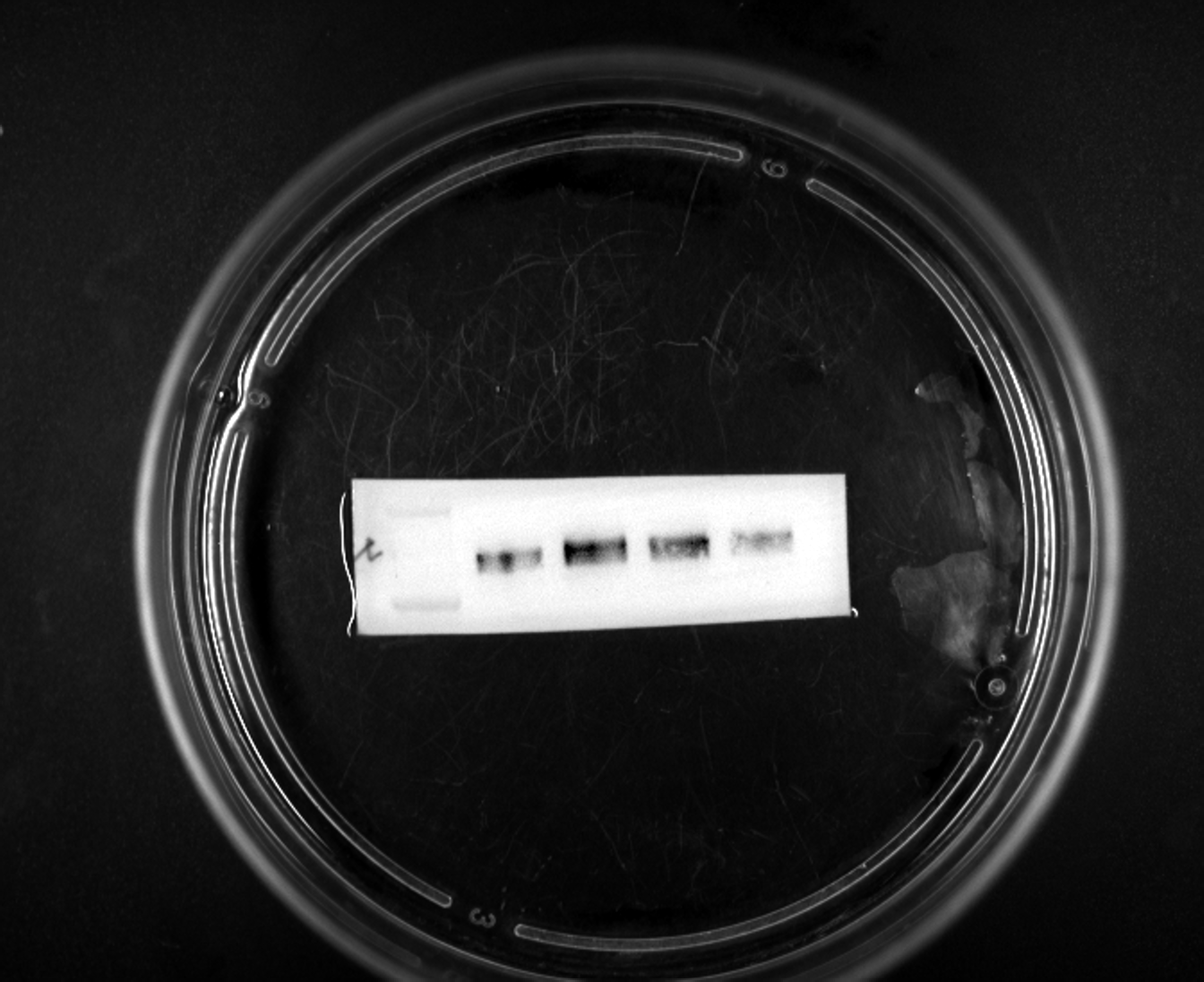

Supplement: Supplementary file 2 [file DataSheet1.ZIP › WB-rawdata/FIG5B/P-FLT3-1.Tif]

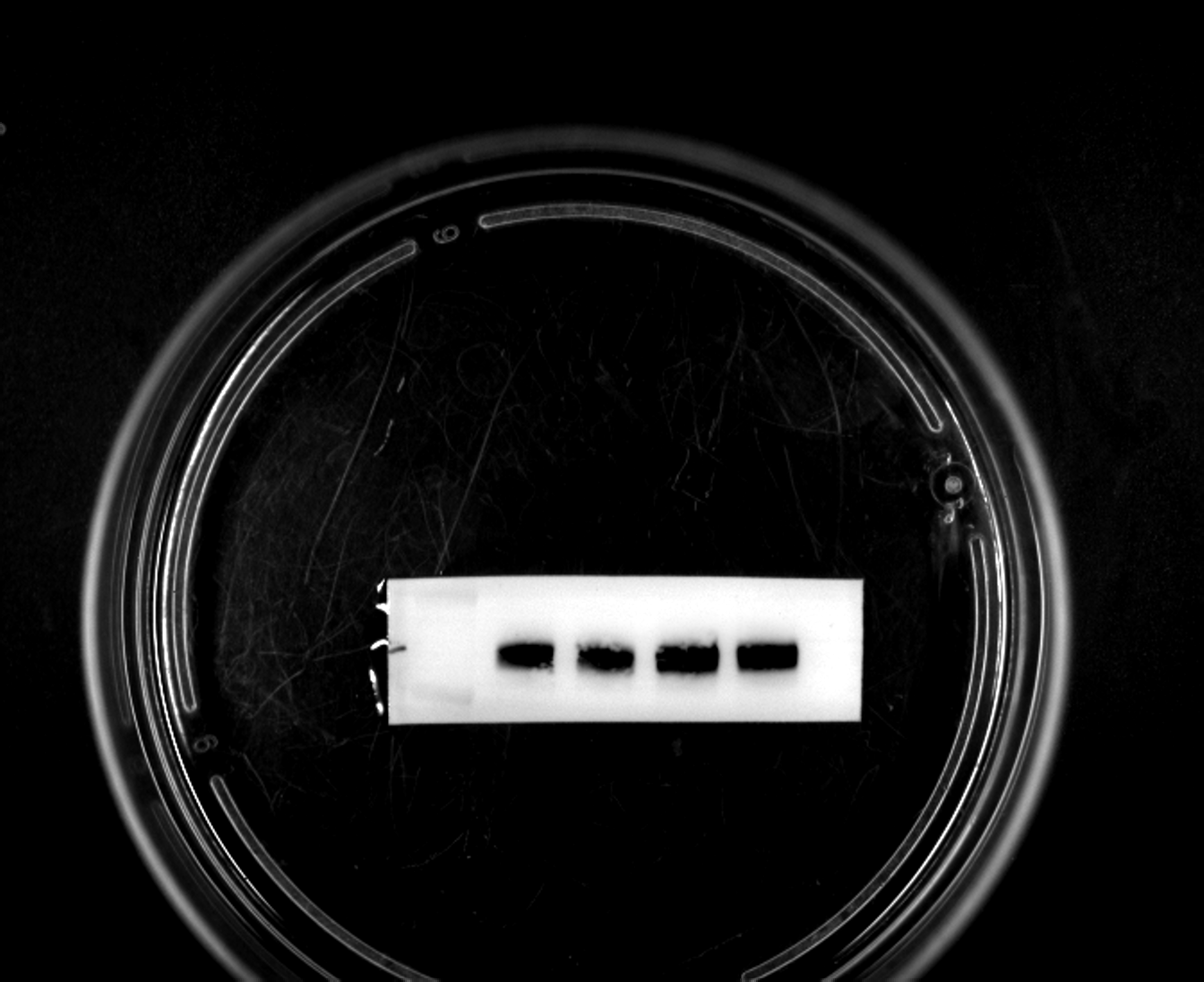

Supplement: Supplementary file 2 [file DataSheet1.ZIP › WB-rawdata/FIG5B/FLT3-3.Tif]

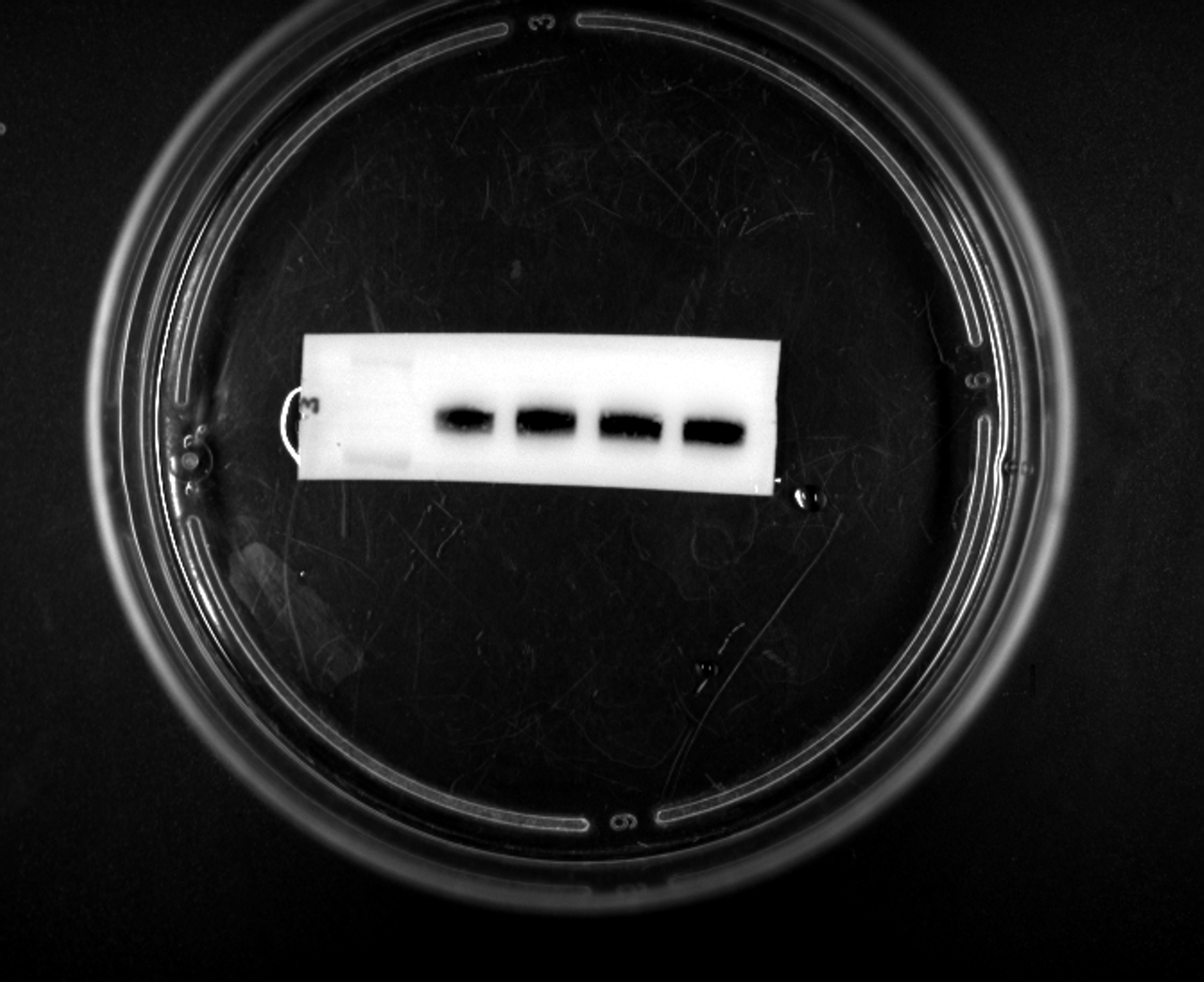

Supplement: Supplementary file 2 [file DataSheet1.ZIP › WB-rawdata/FIG5B/FLT3-2.Tif]

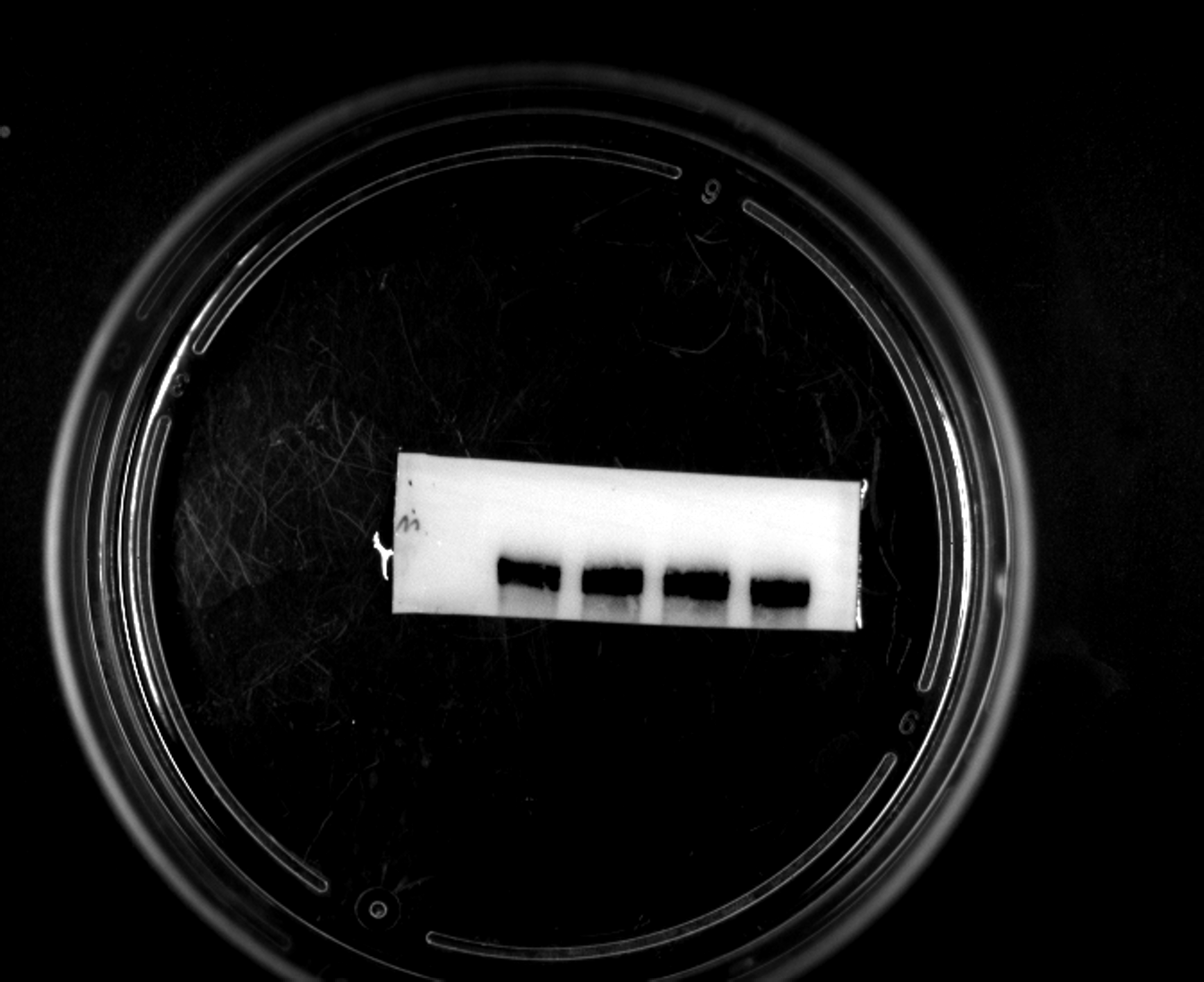

Supplement: Supplementary file 2 [file DataSheet1.ZIP › WB-rawdata/FIG5B/FLT3-1.Tif]

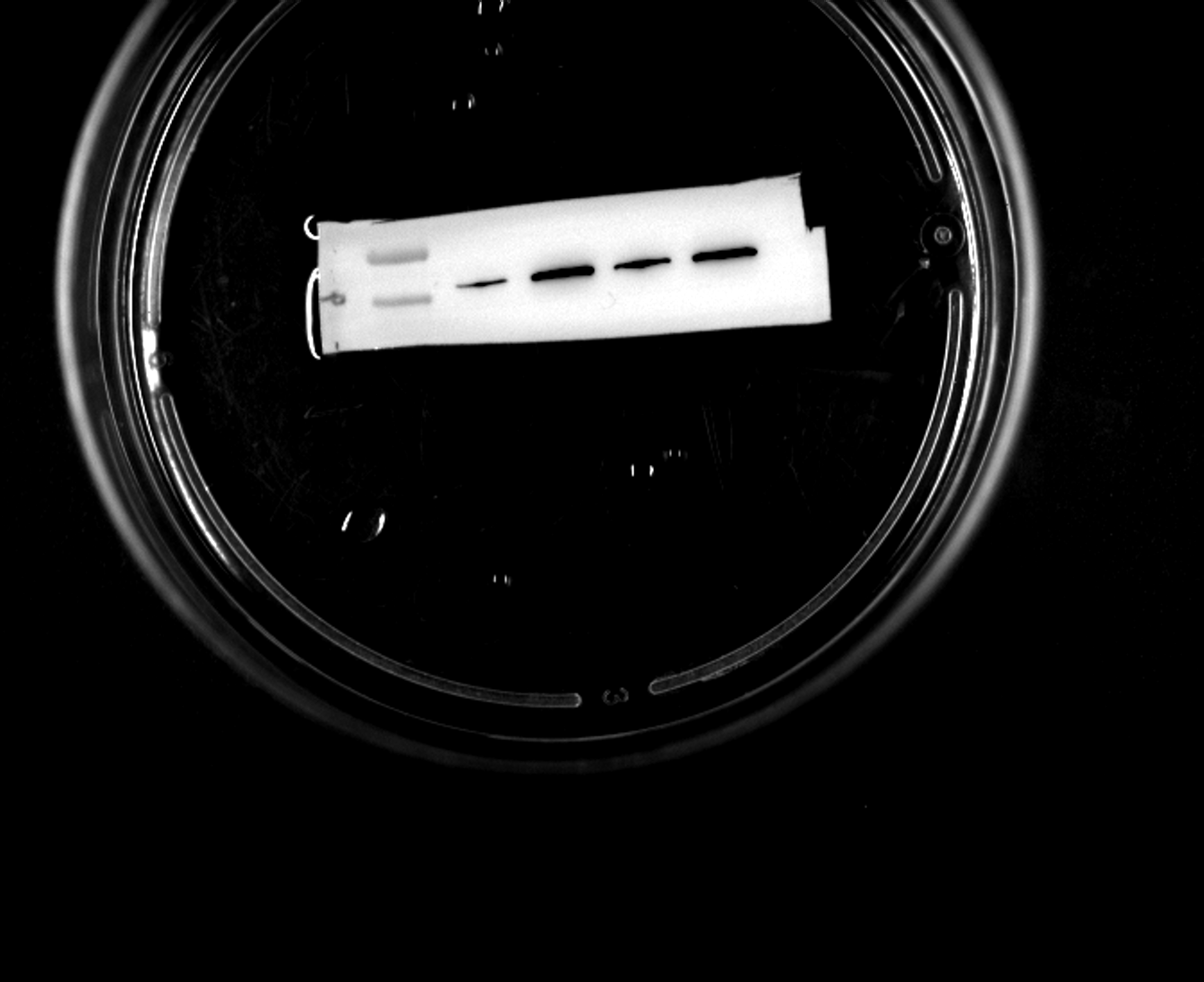

Supplement: Supplementary file 2 [file DataSheet1.ZIP › WB-rawdata/FIG5D/p-akt-1.Tif]

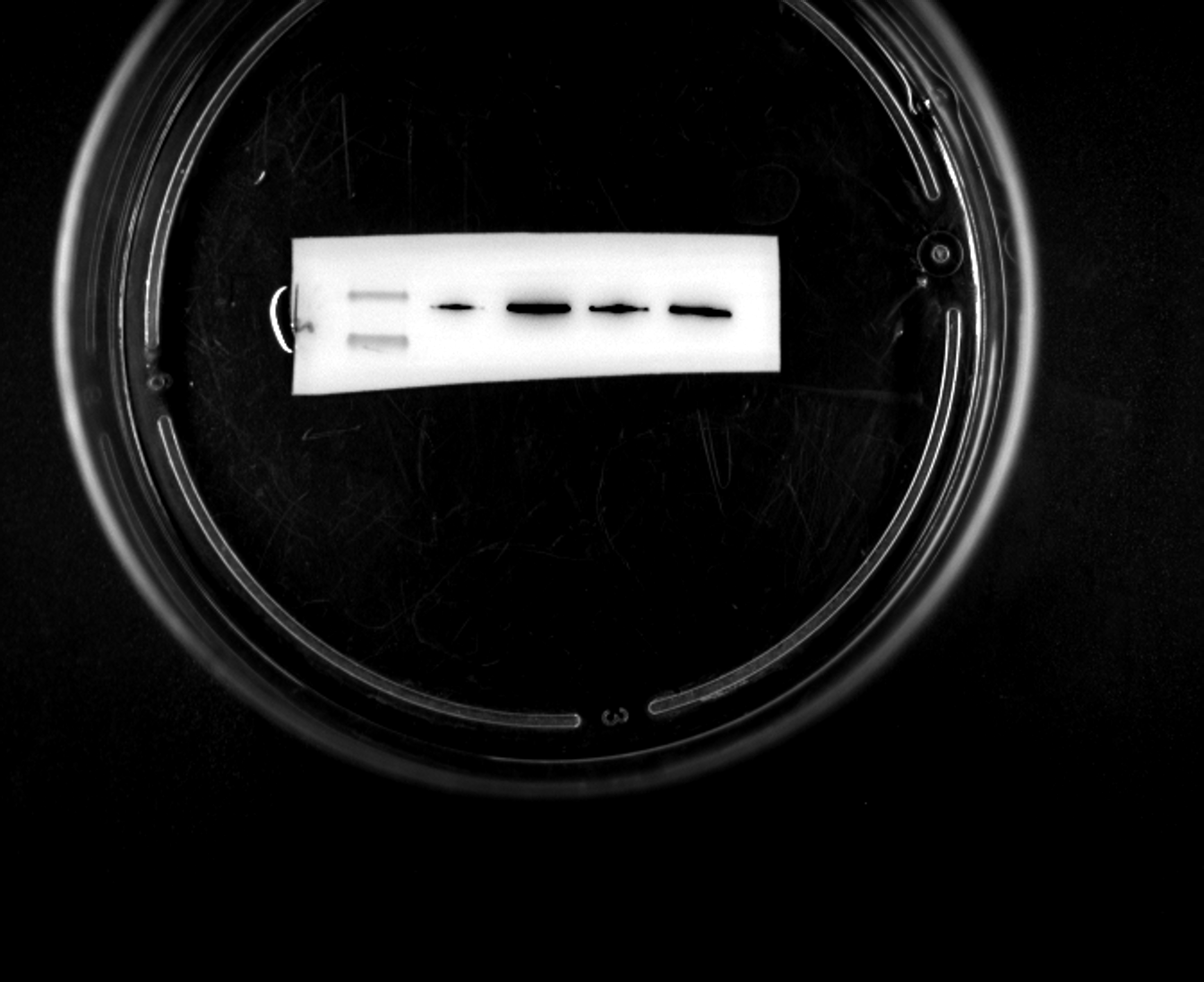

Supplement: Supplementary file 2 [file DataSheet1.ZIP › WB-rawdata/FIG5D/p-akt-2.Tif]

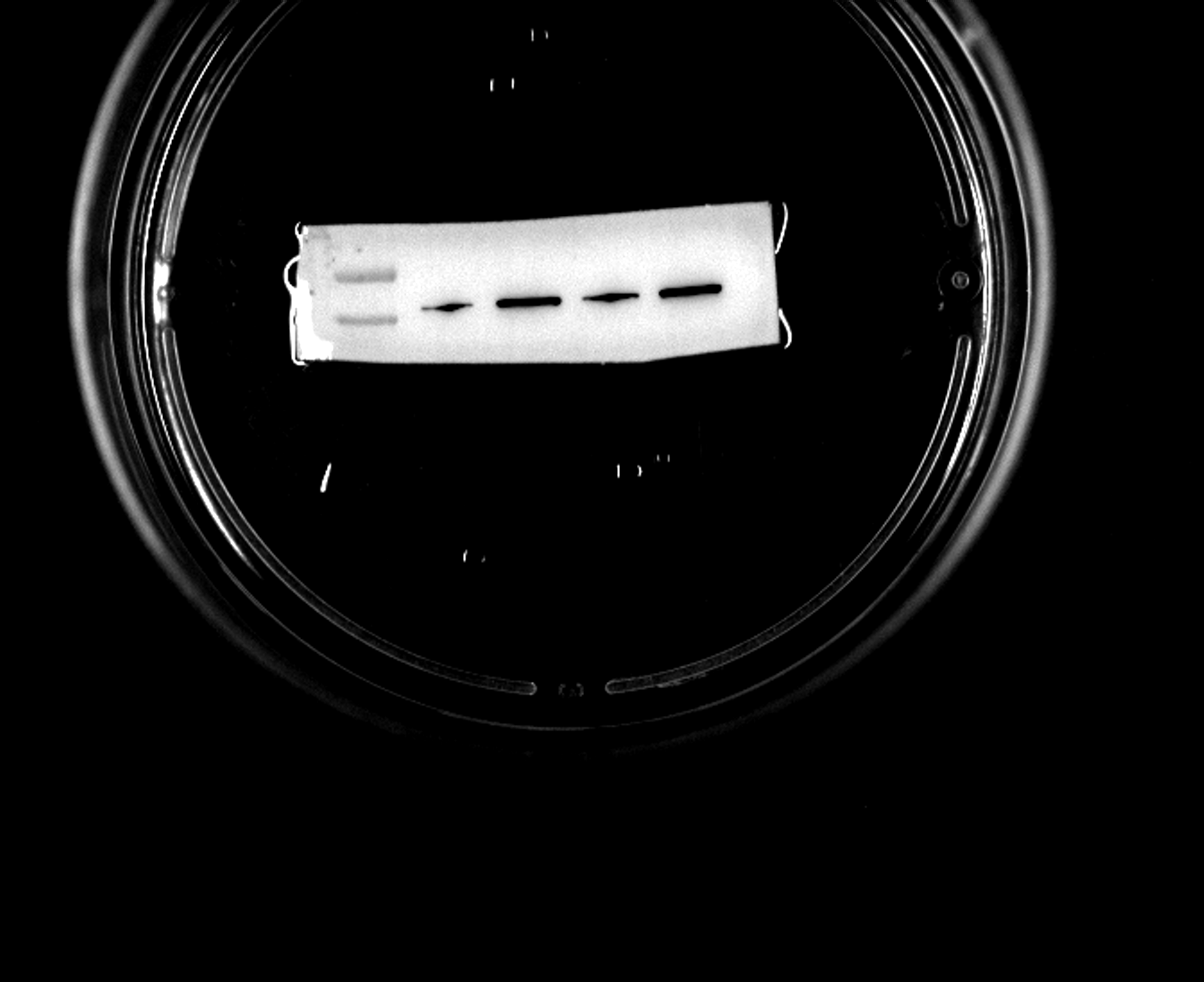

Supplement: Supplementary file 2 [file DataSheet1.ZIP › WB-rawdata/FIG5D/p-akt-3.Tif]

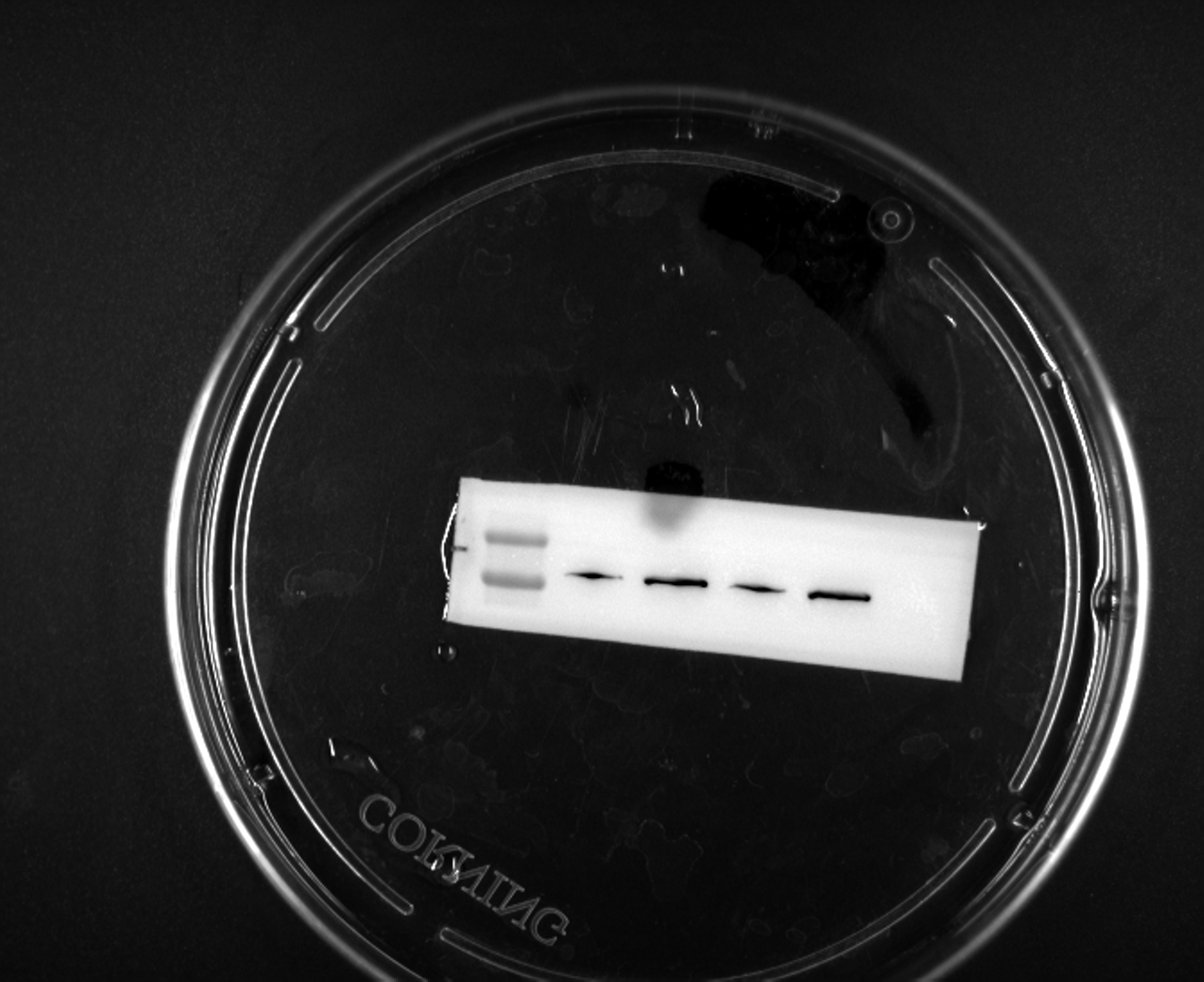

Supplement: Supplementary file 2 [file DataSheet1.ZIP › WB-rawdata/FIG5D/HIF1╬▒-1.Tif]

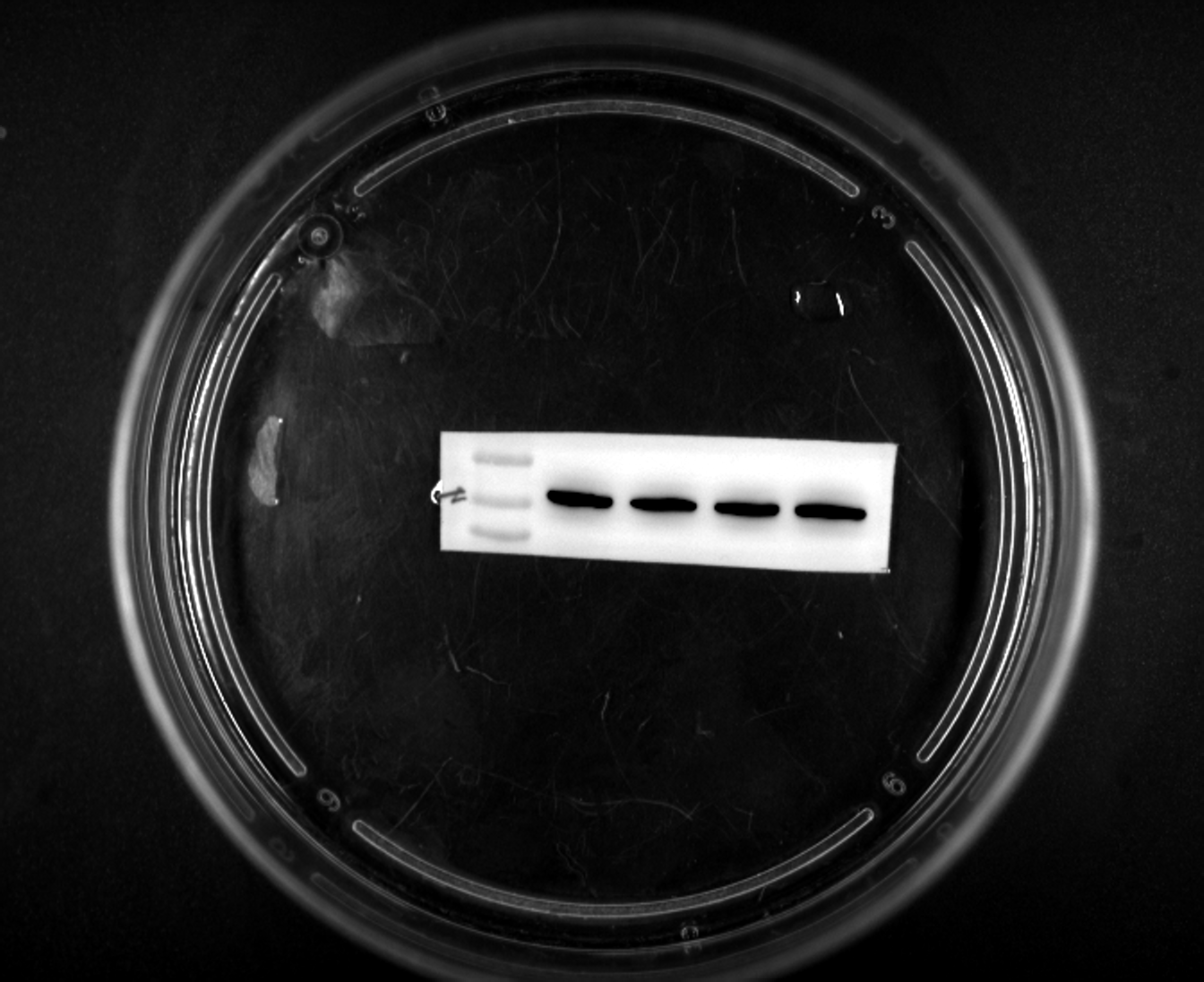

Supplement: Supplementary file 2 [file DataSheet1.ZIP › WB-rawdata/FIG5D/actin-1.Tif]

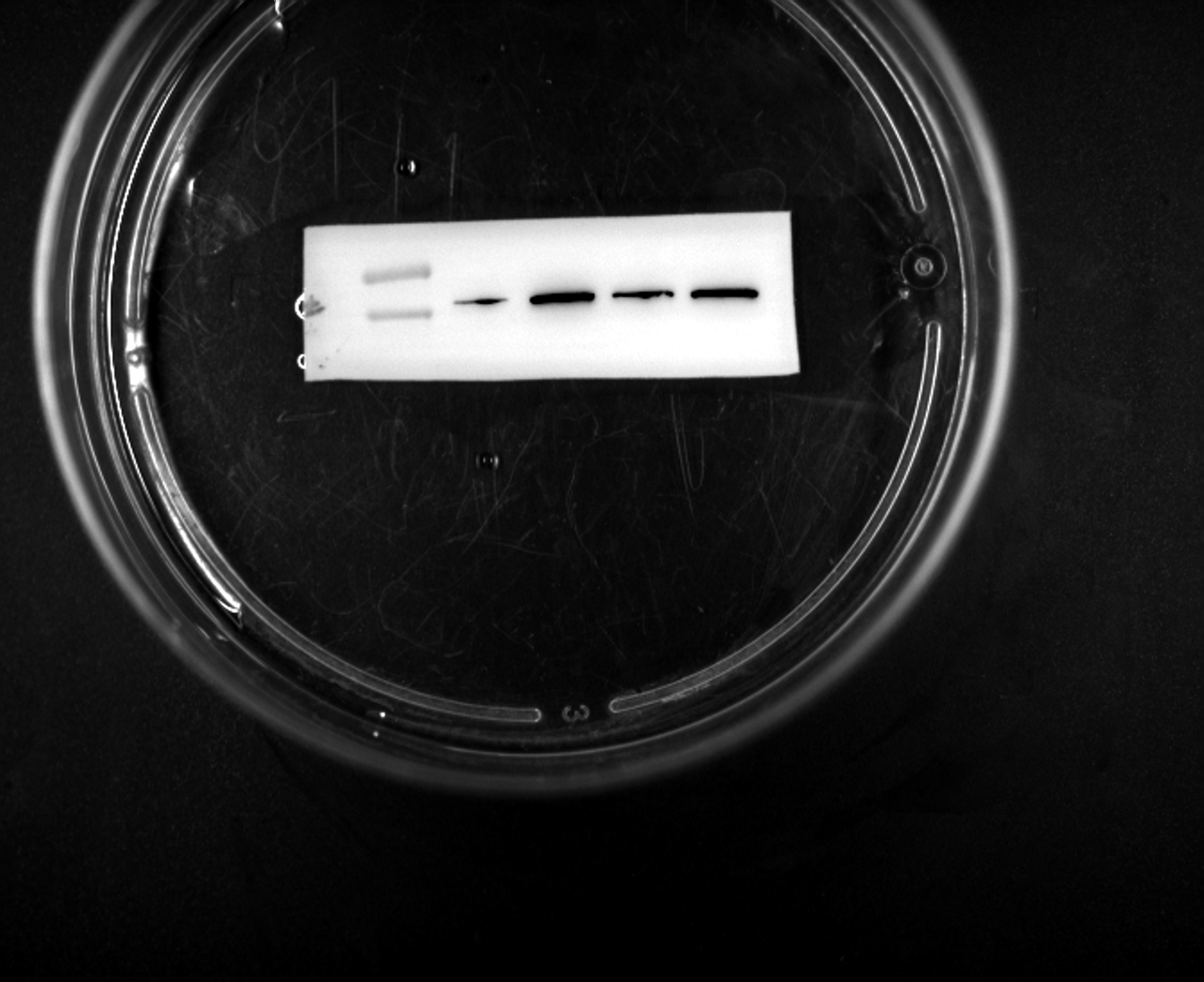

Supplement: Supplementary file 2 [file DataSheet1.ZIP › WB-rawdata/FIG5D/HIF1╬▒-2.Tif]

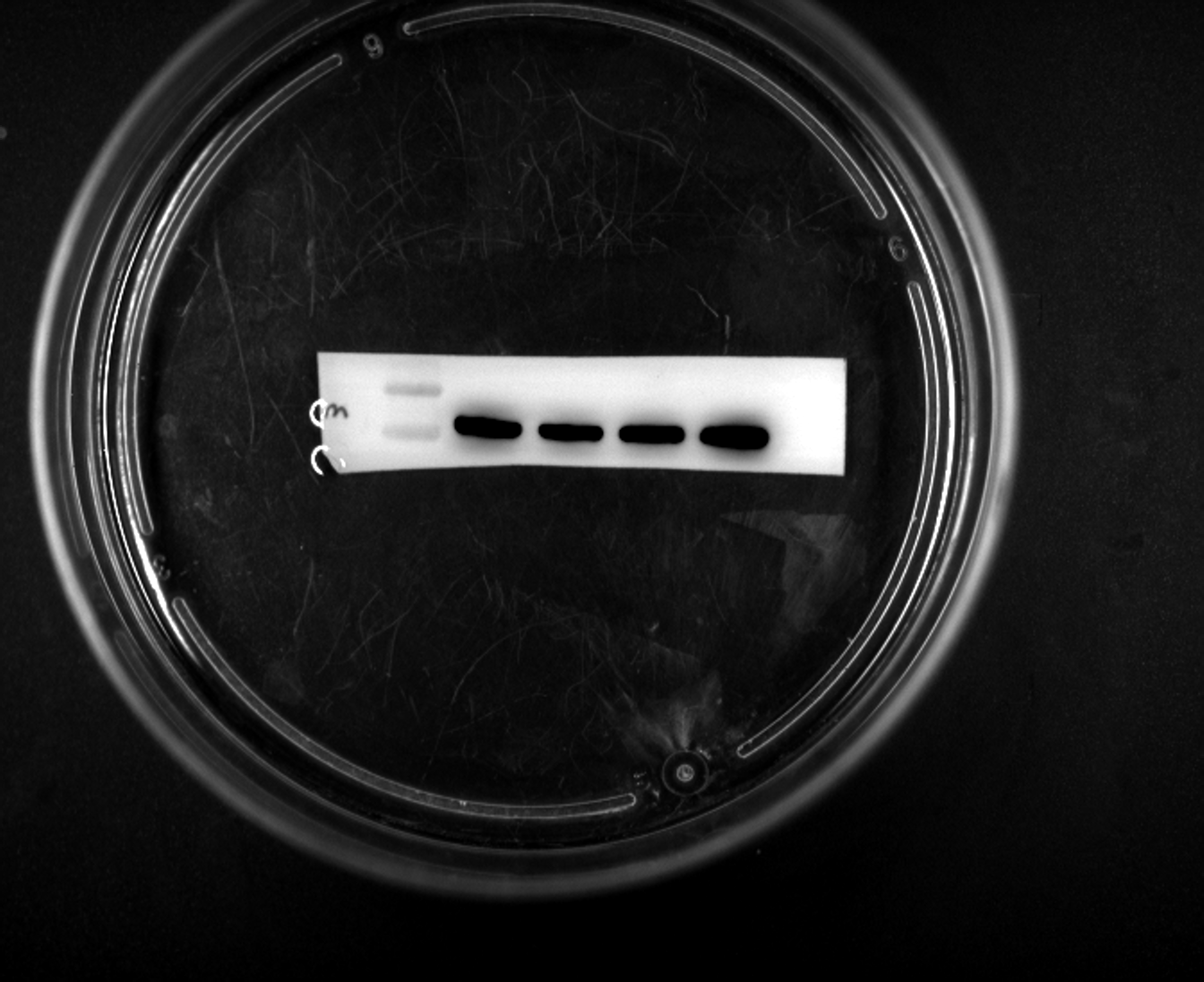

Supplement: Supplementary file 2 [file DataSheet1.ZIP › WB-rawdata/FIG5D/actin-3.Tif]

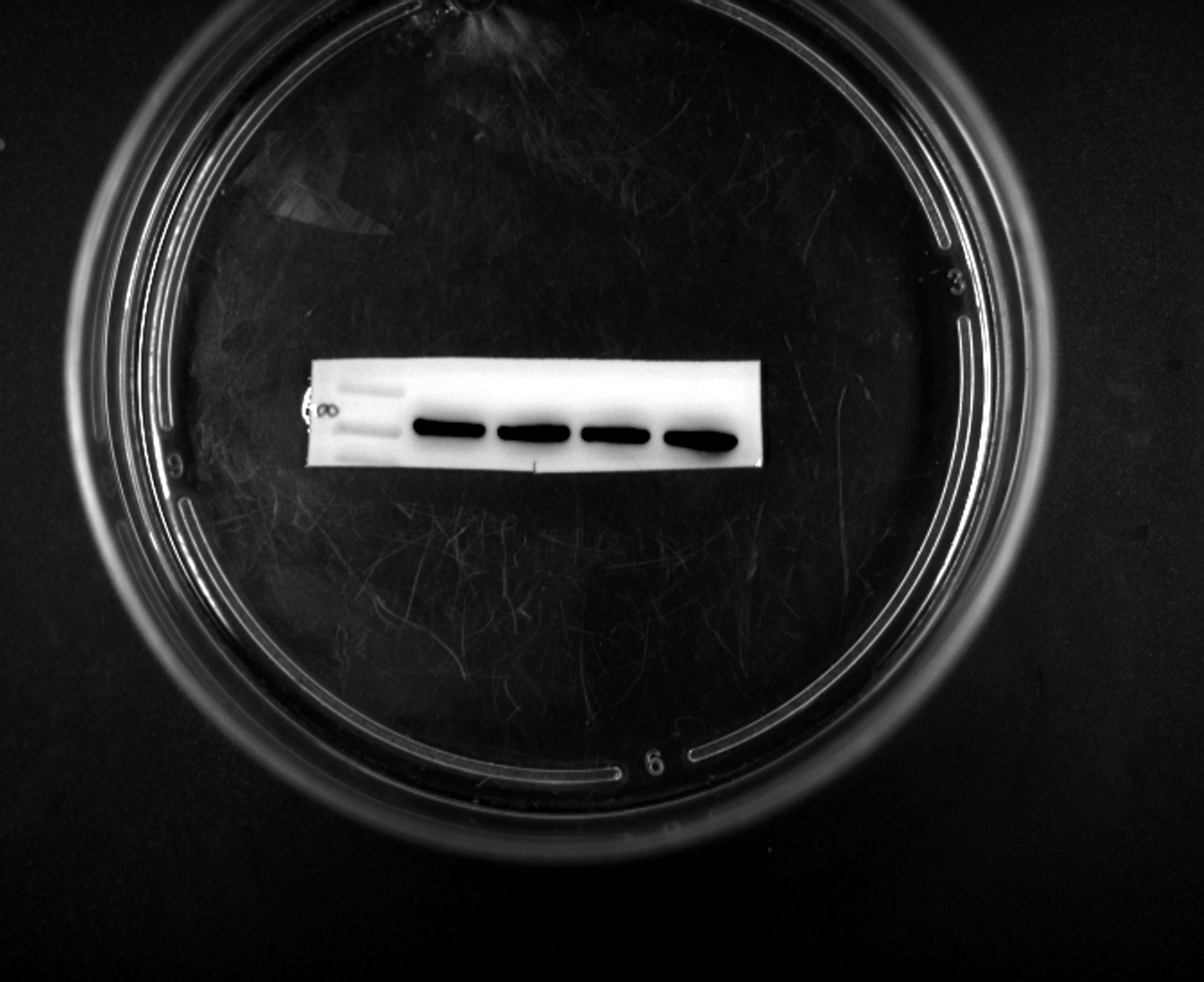

Supplement: Supplementary file 2 [file DataSheet1.ZIP › WB-rawdata/FIG5D/actin-2.Tif]

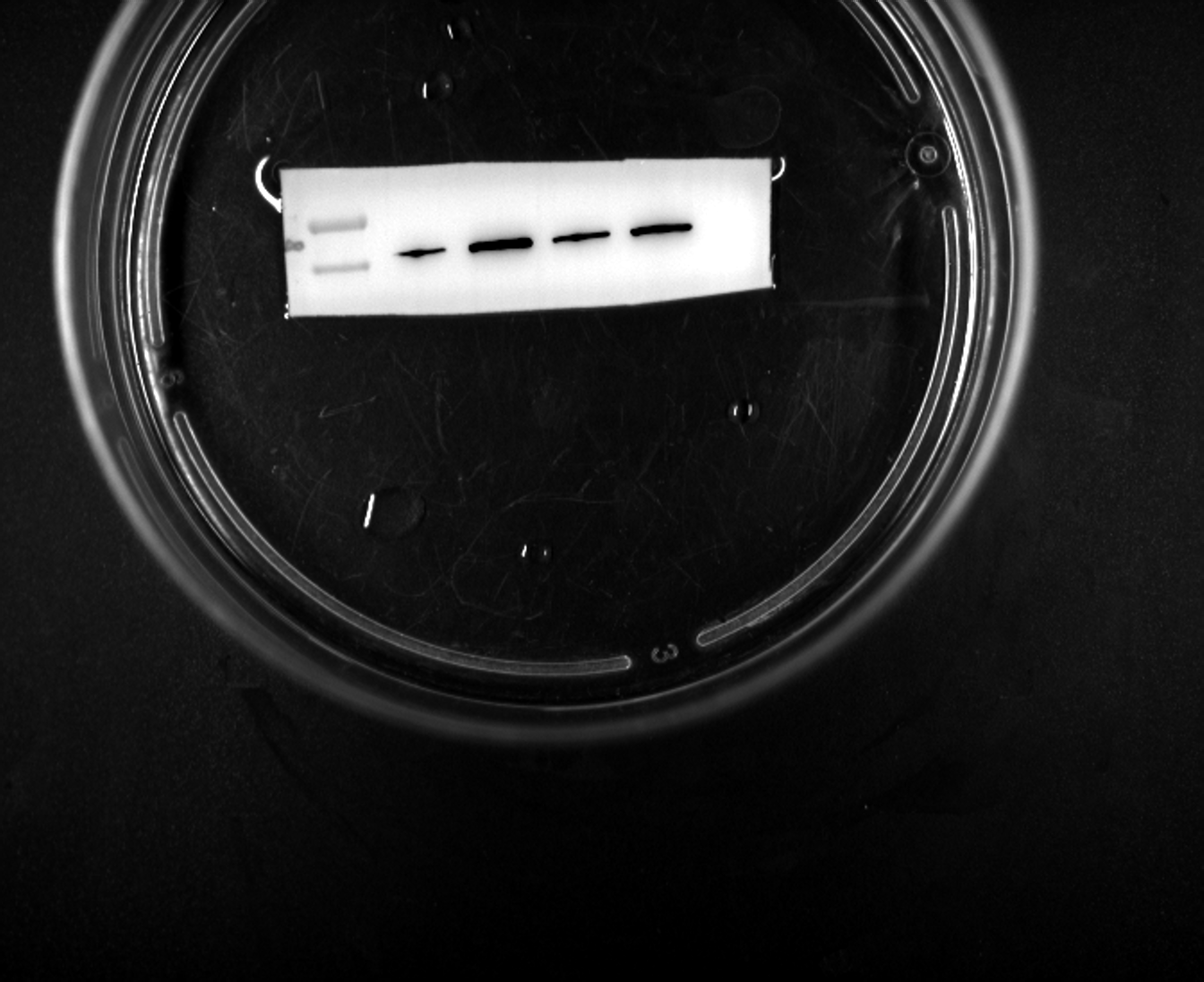

Supplement: Supplementary file 2 [file DataSheet1.ZIP › WB-rawdata/FIG5D/HIF1╬▒-3.Tif]

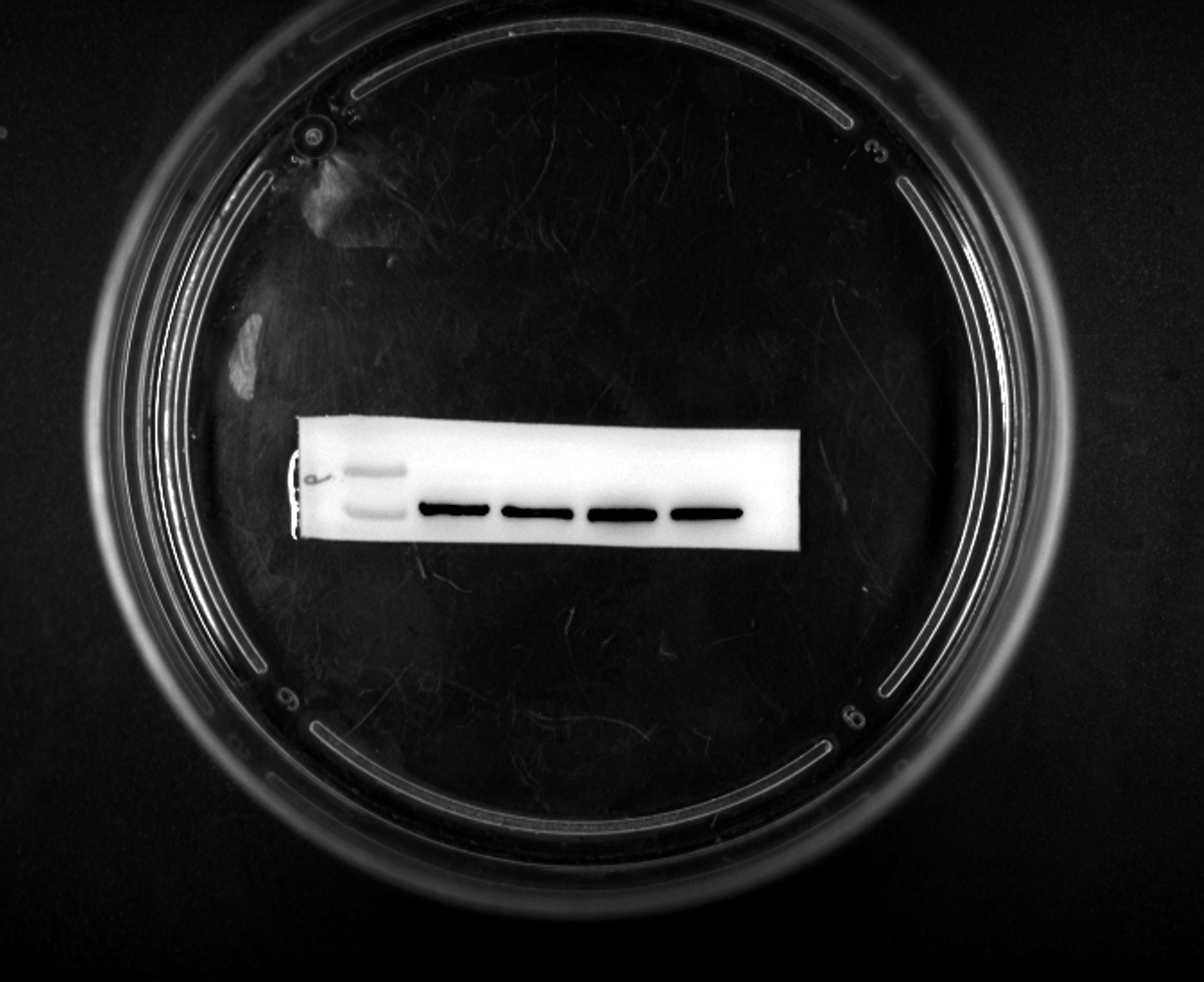

Supplement: Supplementary file 2 [file DataSheet1.ZIP › WB-rawdata/FIG5D/AKT-1.Tif]

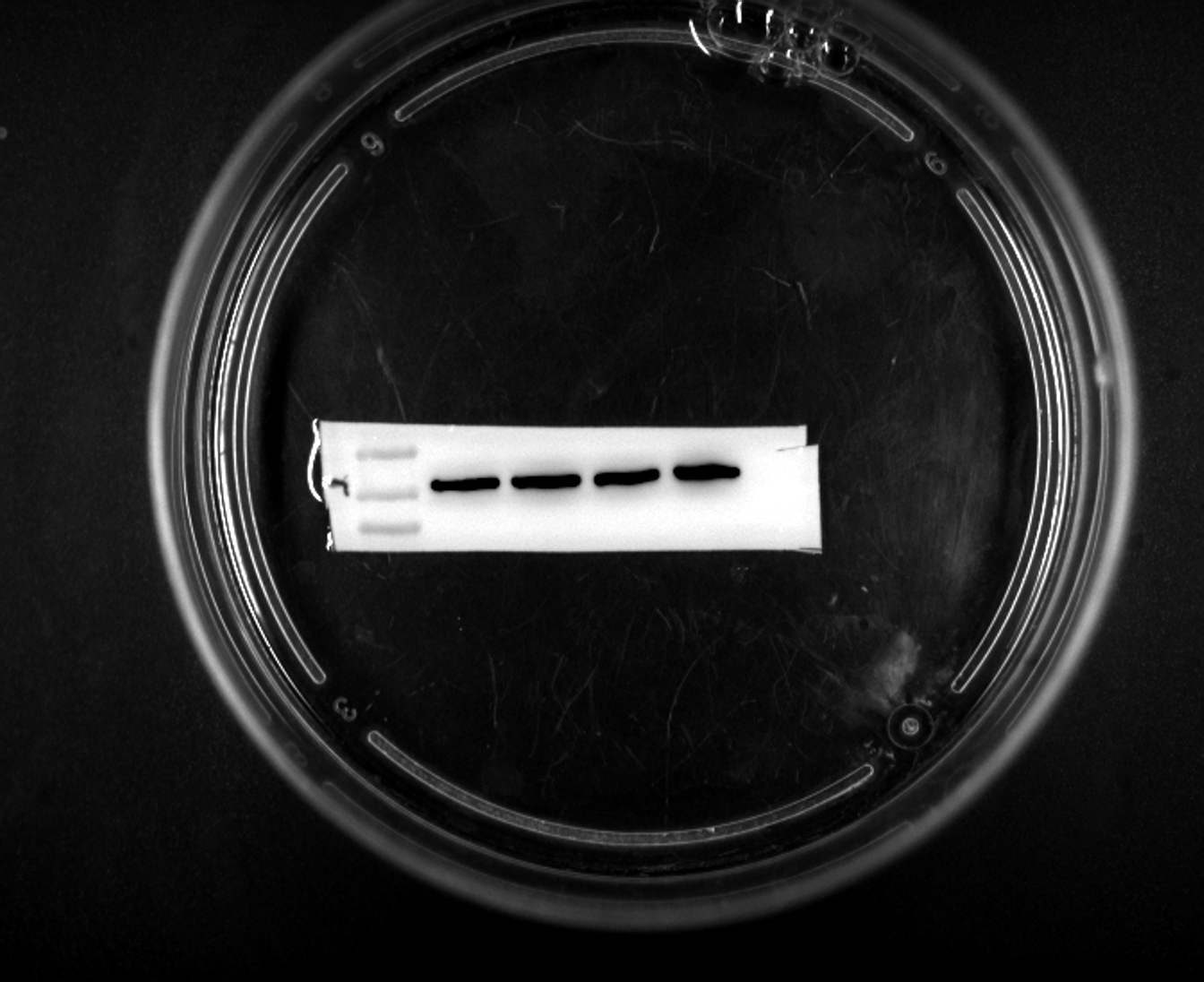

Supplement: Supplementary file 2 [file DataSheet1.ZIP › WB-rawdata/FIG5D/AKT-3.Tif]

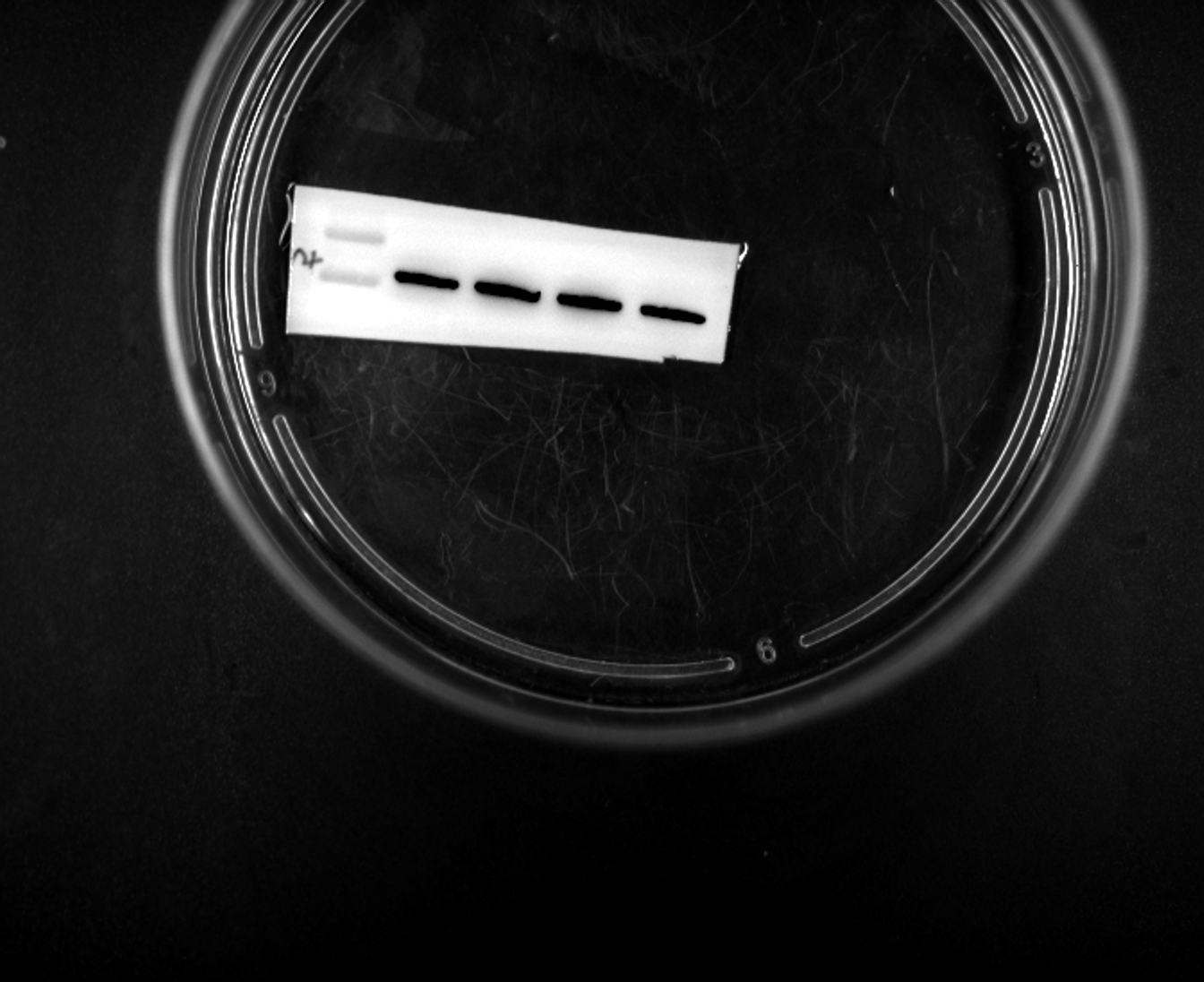

Supplement: Supplementary file 2 [file DataSheet1.ZIP › WB-rawdata/FIG5D/AKT-2.Tif]

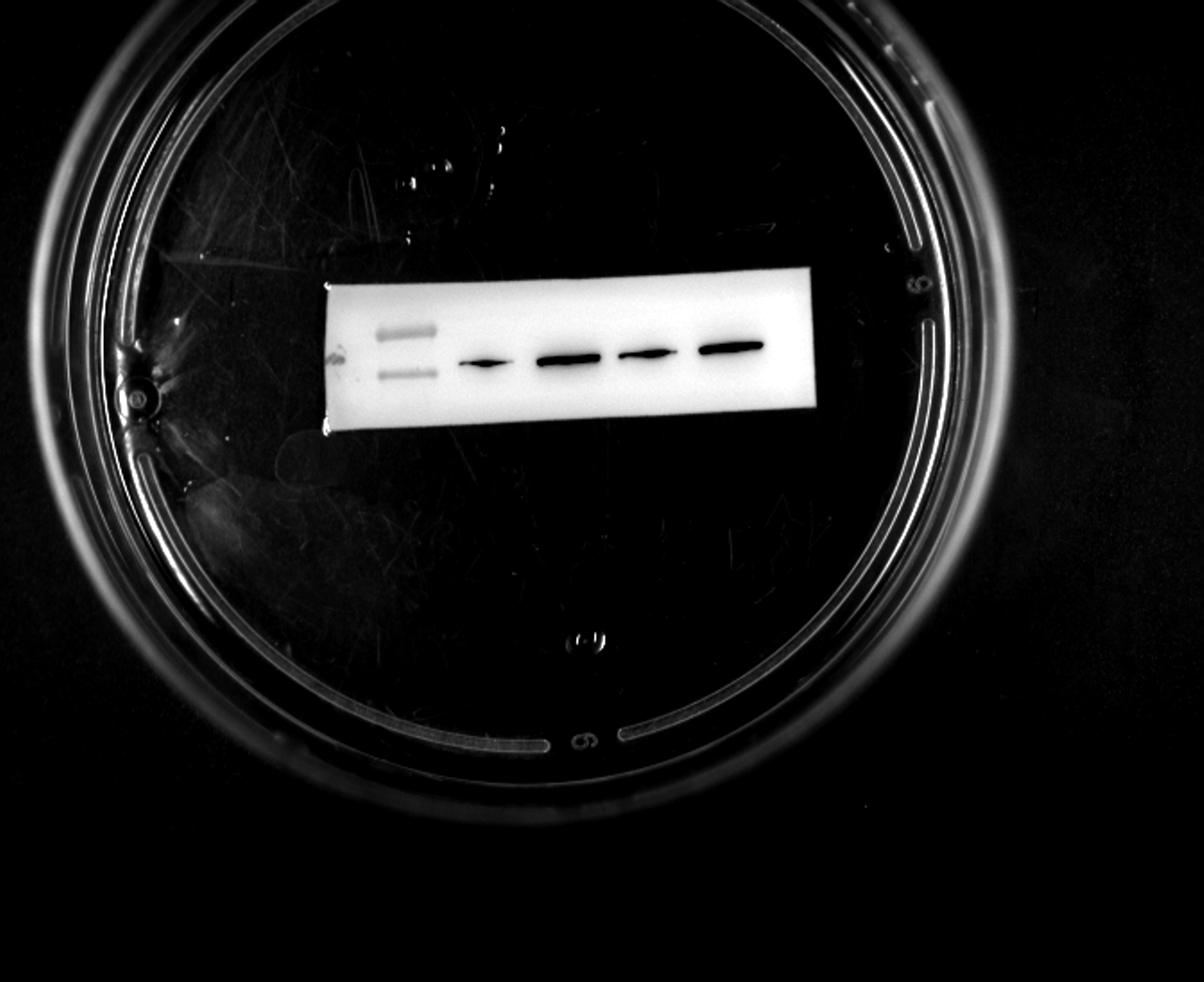

Supplement: Supplementary file 2 [file DataSheet1.ZIP › WB-rawdata/FIG5D/PI3K-1.Tif]

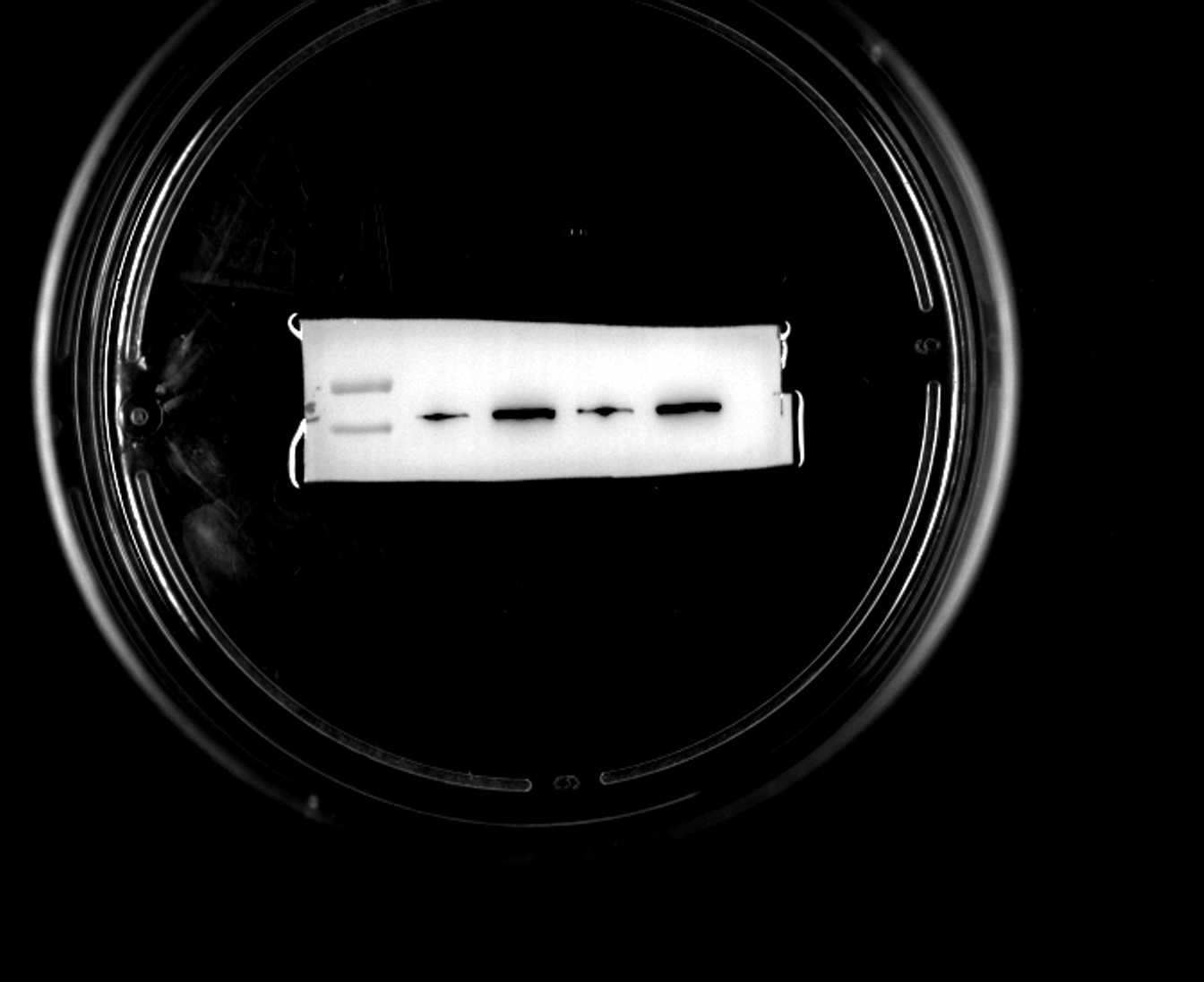

Supplement: Supplementary file 2 [file DataSheet1.ZIP › WB-rawdata/FIG5D/PI3K-2.Tif]

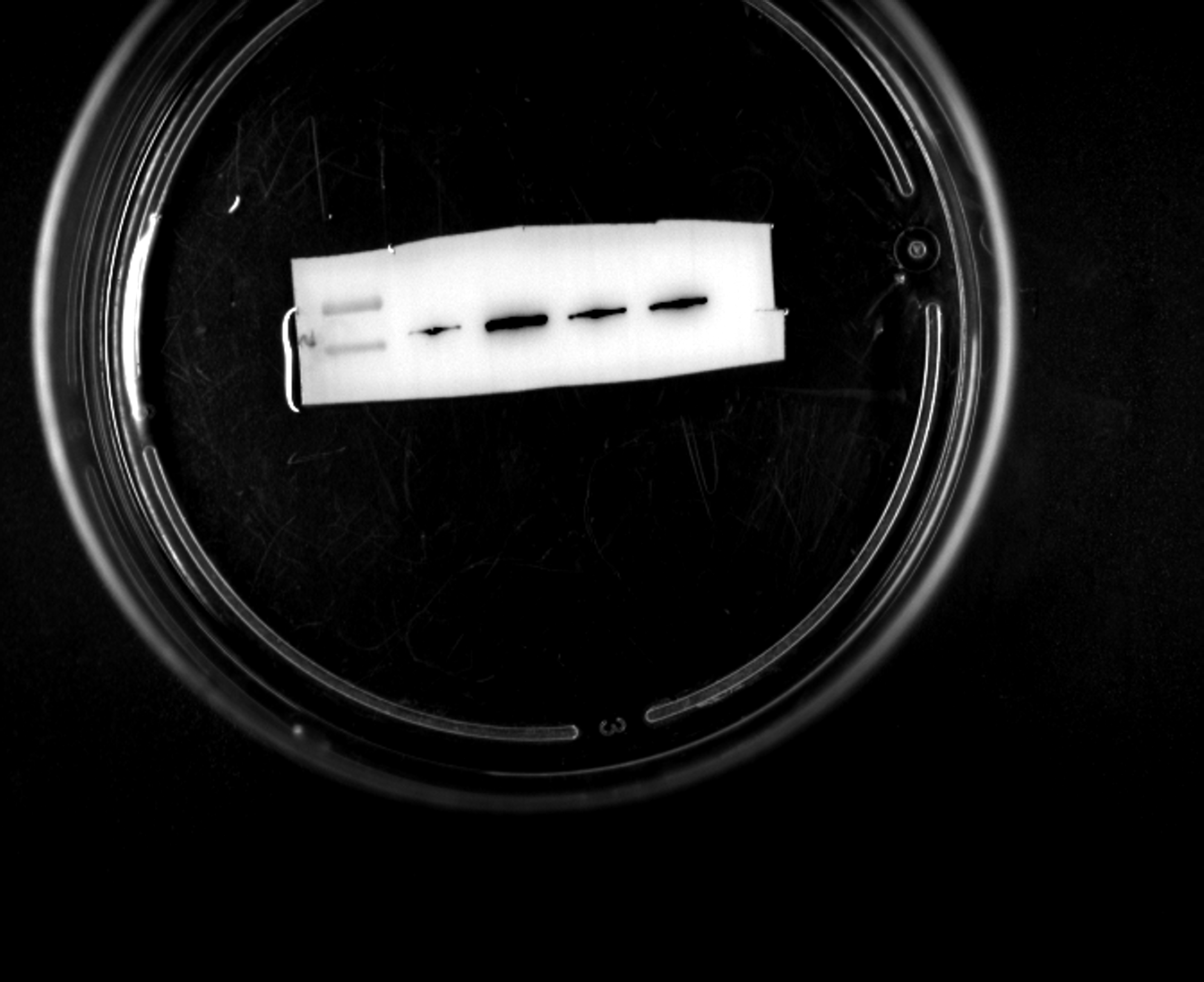

Supplement: Supplementary file 2 [file DataSheet1.ZIP › WB-rawdata/FIG5D/PI3K-3.Tif]

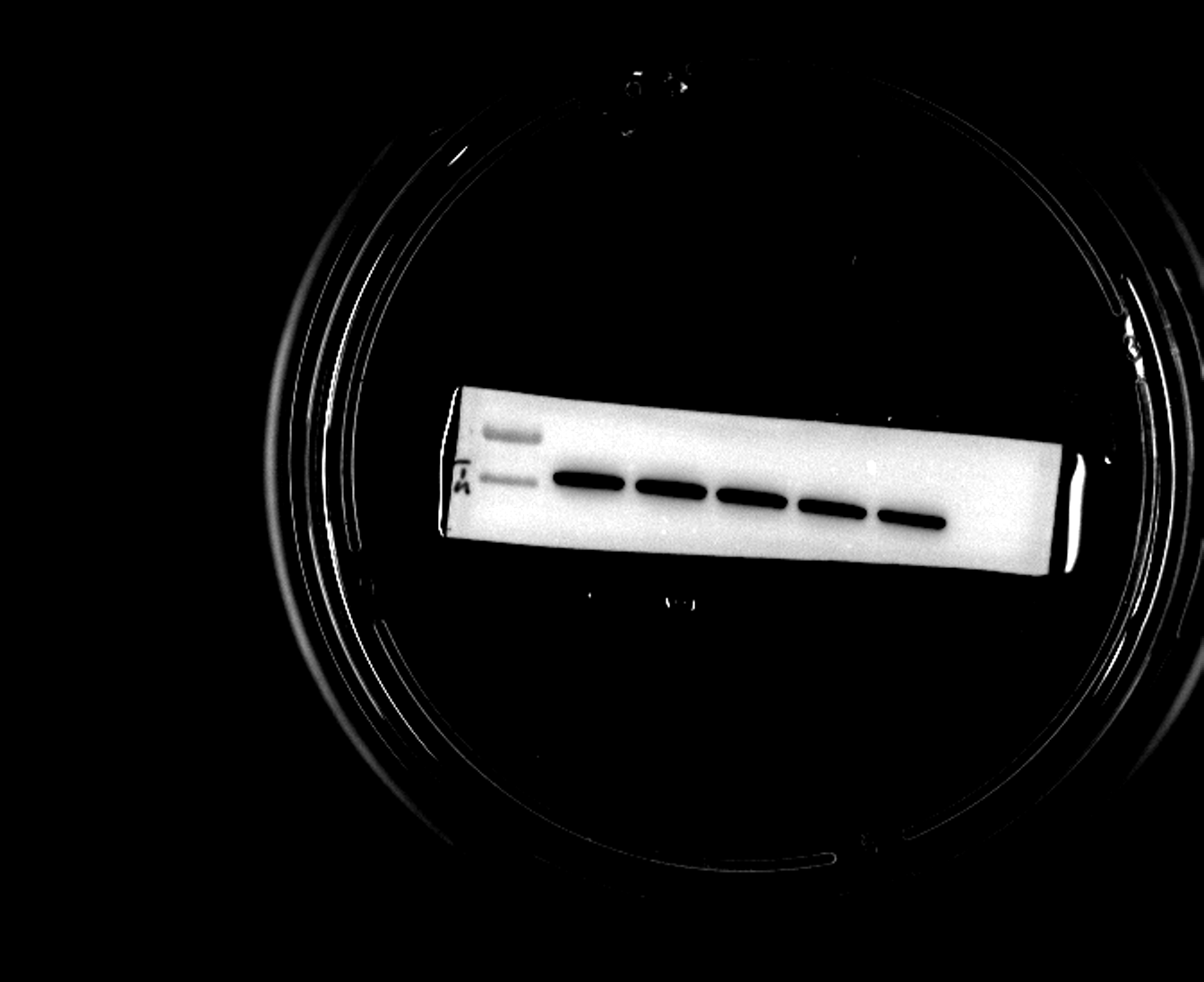

Supplement: Supplementary file 2 [file DataSheet1.ZIP › WB-rawdata/FIG4A/actin-1.Tif]

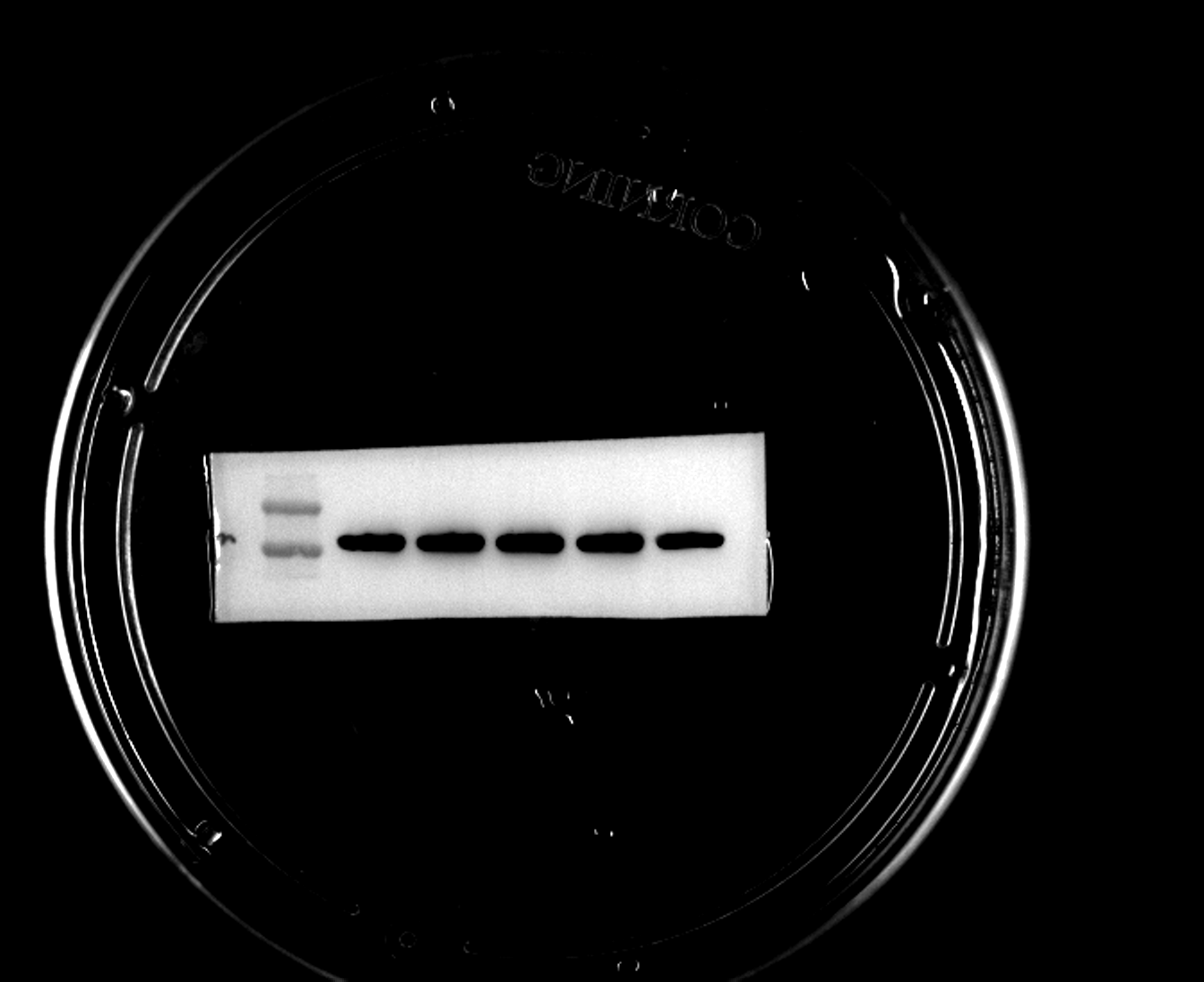

Supplement: Supplementary file 2 [file DataSheet1.ZIP › WB-rawdata/FIG4A/actin-3.Tif]

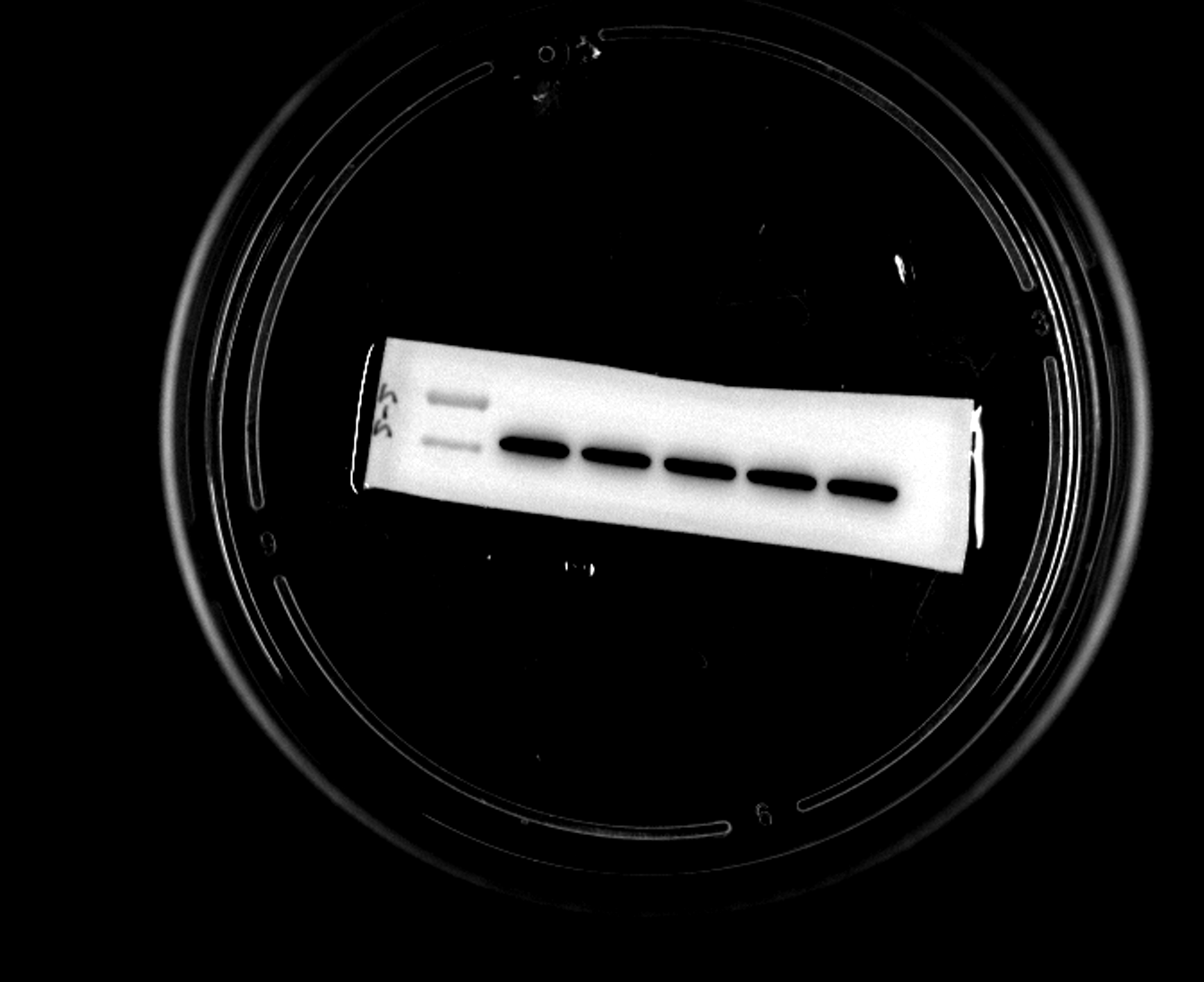

Supplement: Supplementary file 2 [file DataSheet1.ZIP › WB-rawdata/FIG4A/actin-2.Tif]

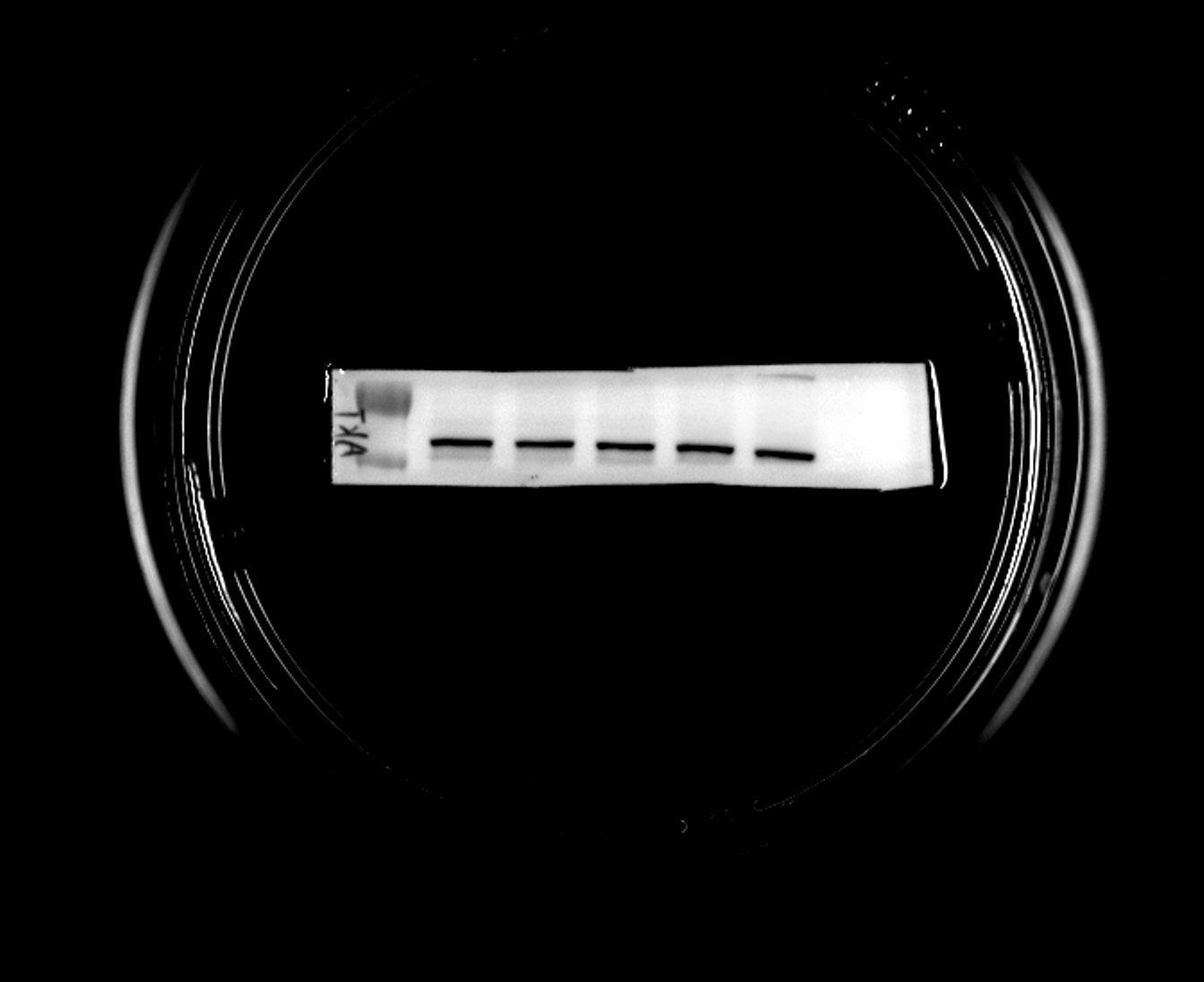

Supplement: Supplementary file 2 [file DataSheet1.ZIP › WB-rawdata/FIG4A/AKT-1.Tif]

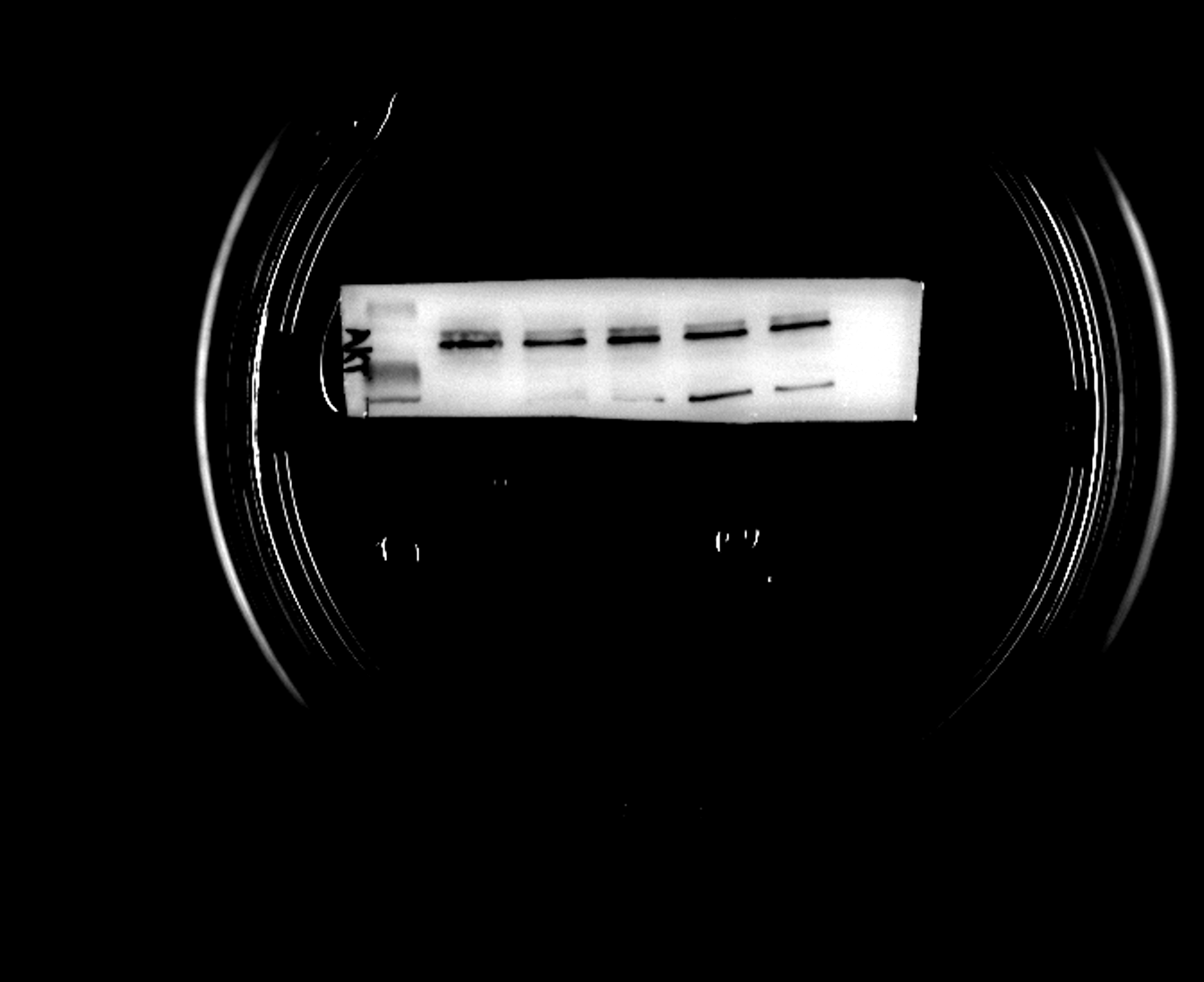

Supplement: Supplementary file 2 [file DataSheet1.ZIP › WB-rawdata/FIG4A/AKT-3.Tif]

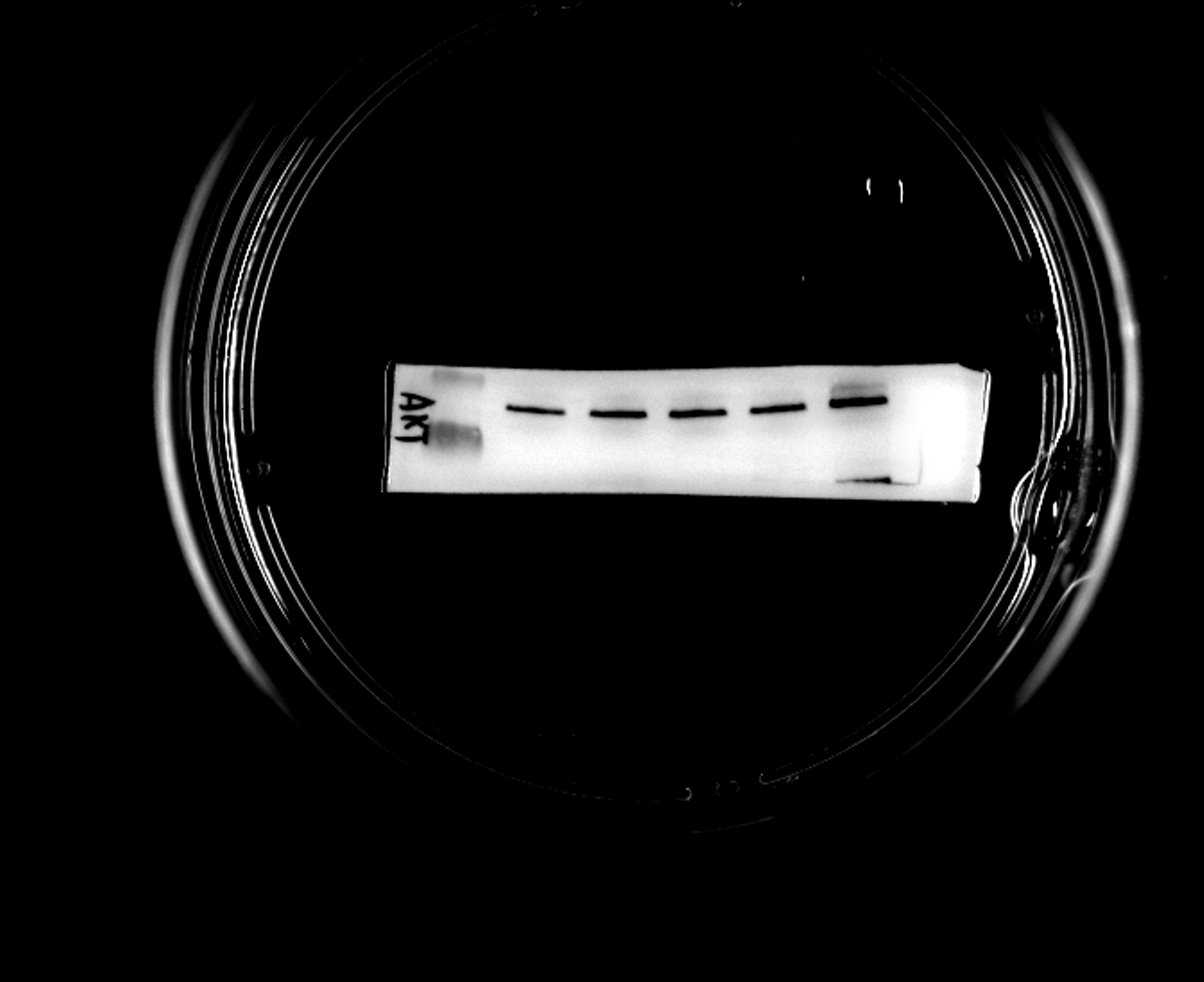

Supplement: Supplementary file 2 [file DataSheet1.ZIP › WB-rawdata/FIG4A/AKT-2.Tif]

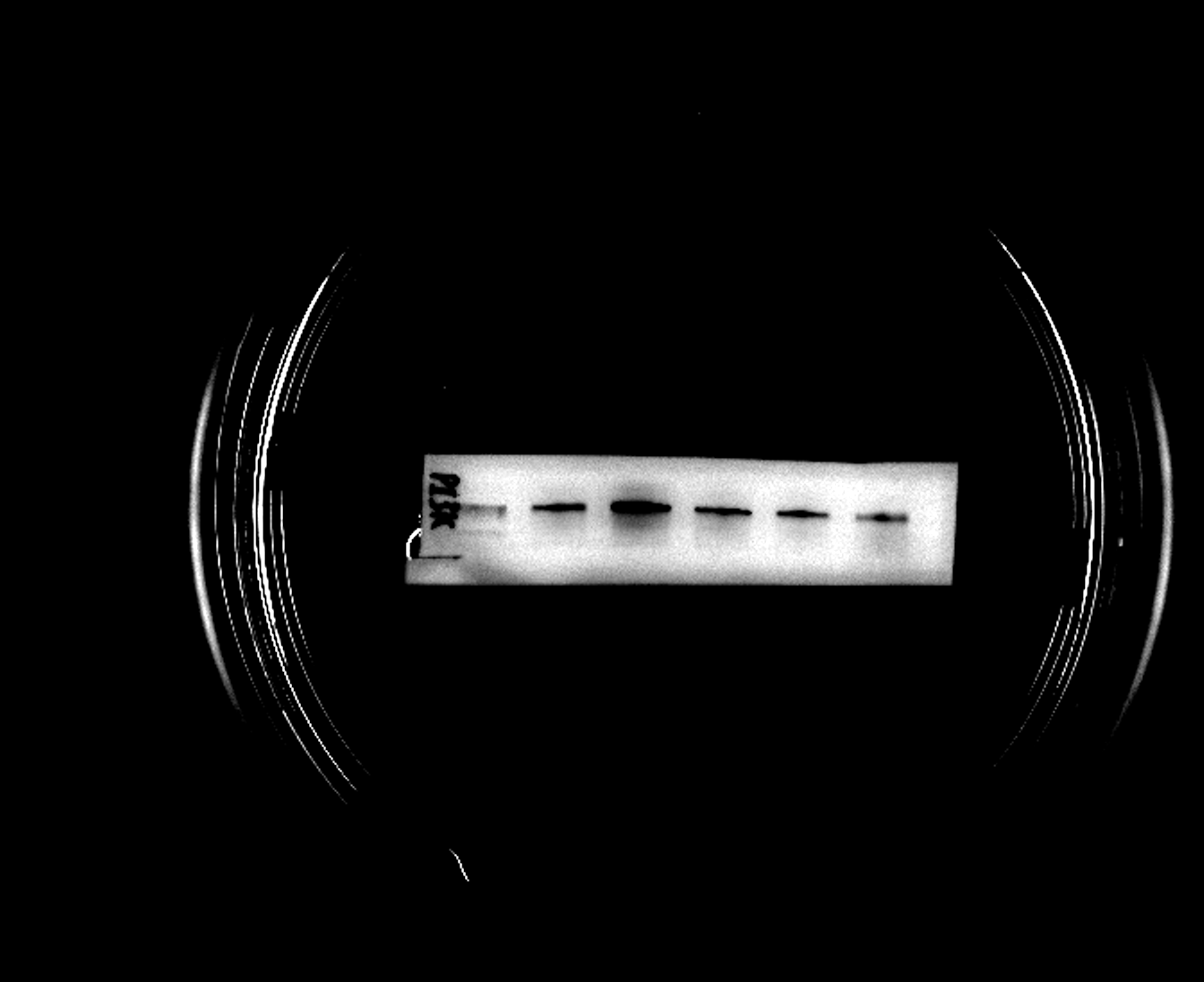

Supplement: Supplementary file 2 [file DataSheet1.ZIP › WB-rawdata/FIG4A/PI3K-1.Tif]

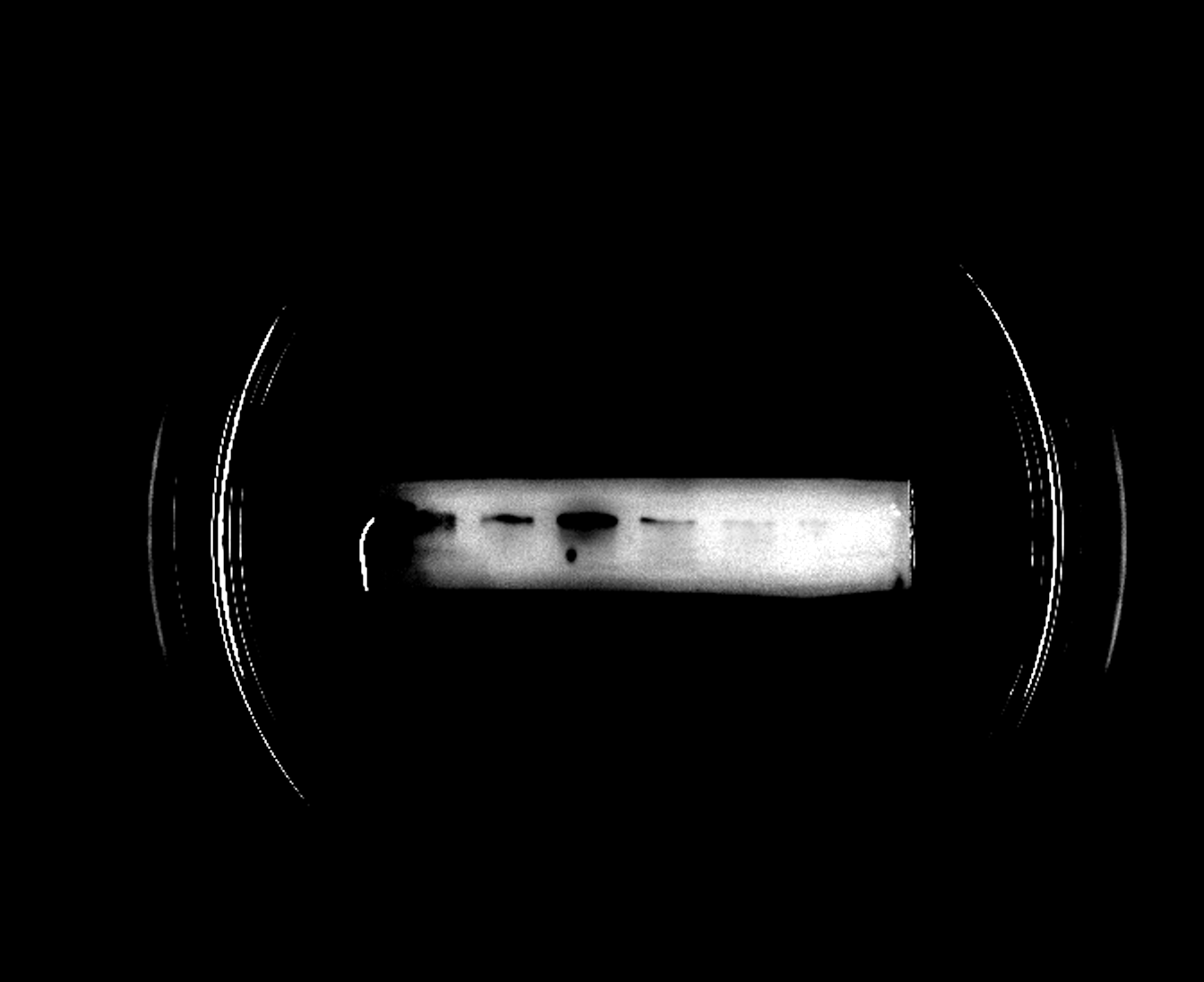

Supplement: Supplementary file 2 [file DataSheet1.ZIP › WB-rawdata/FIG4A/PI3K-2.Tif]

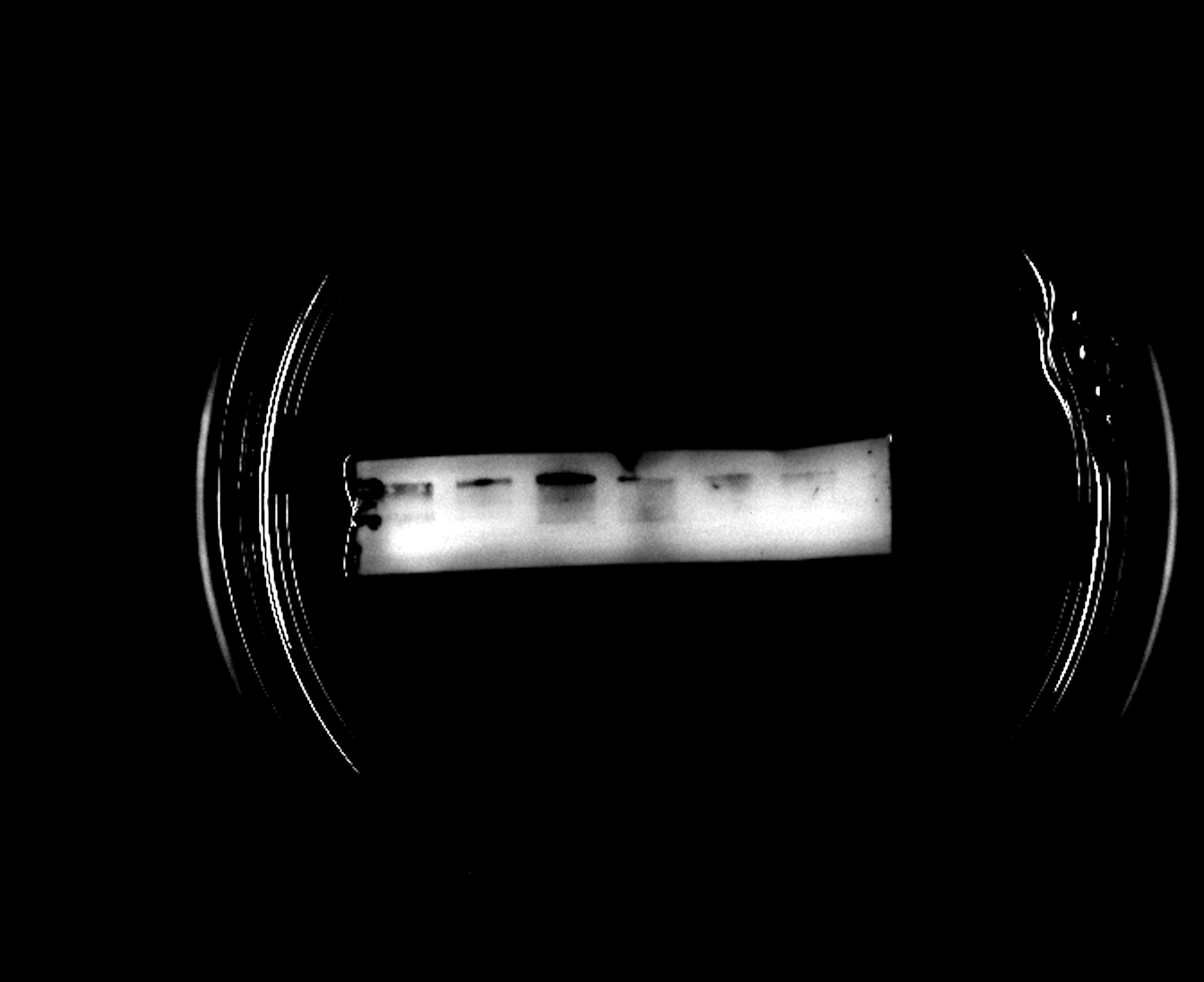

Supplement: Supplementary file 2 [file DataSheet1.ZIP › WB-rawdata/FIG4A/PI3K-3.Tif]

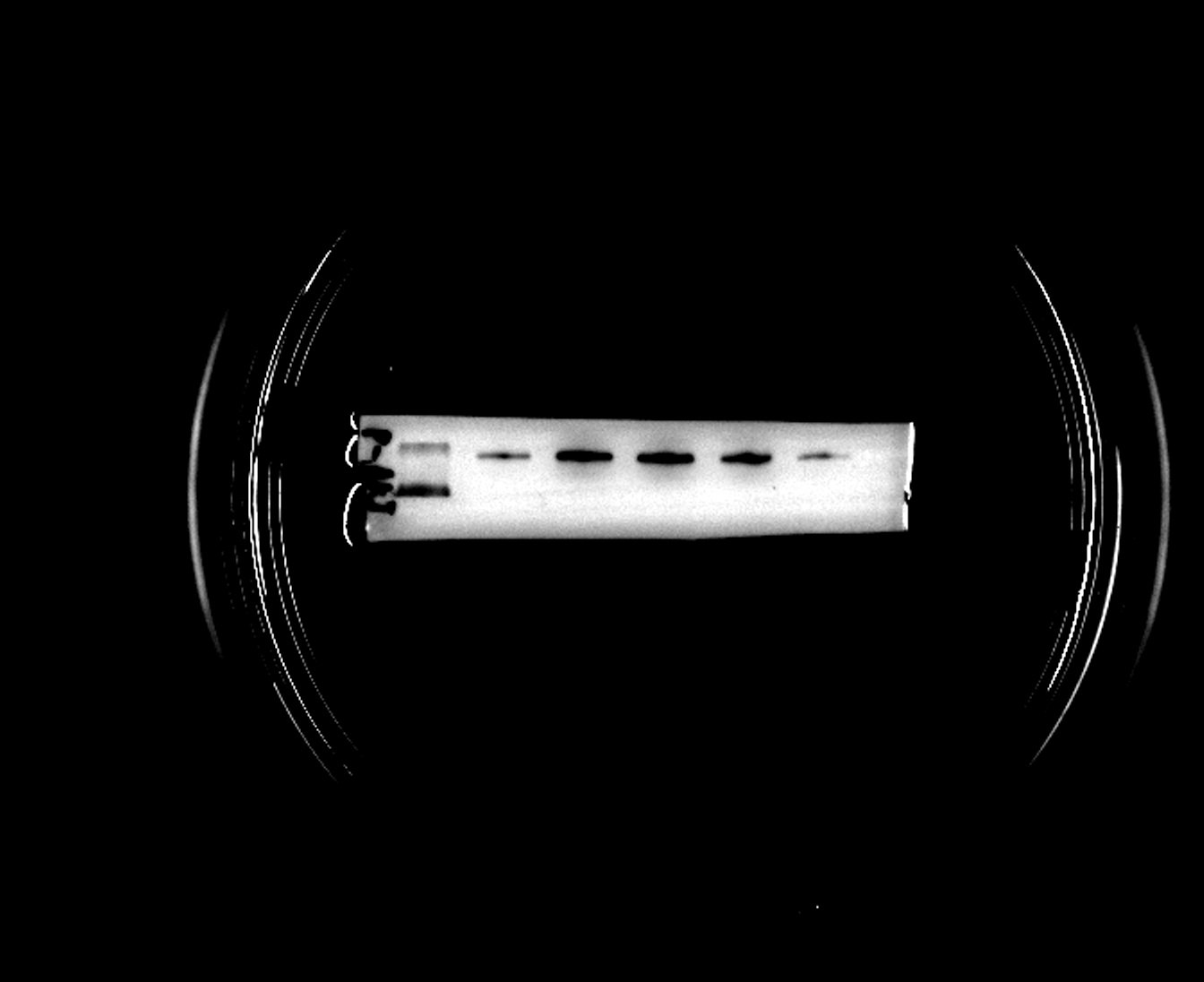

Supplement: Supplementary file 2 [file DataSheet1.ZIP › WB-rawdata/FIG4A/PAKT-3.Tif]

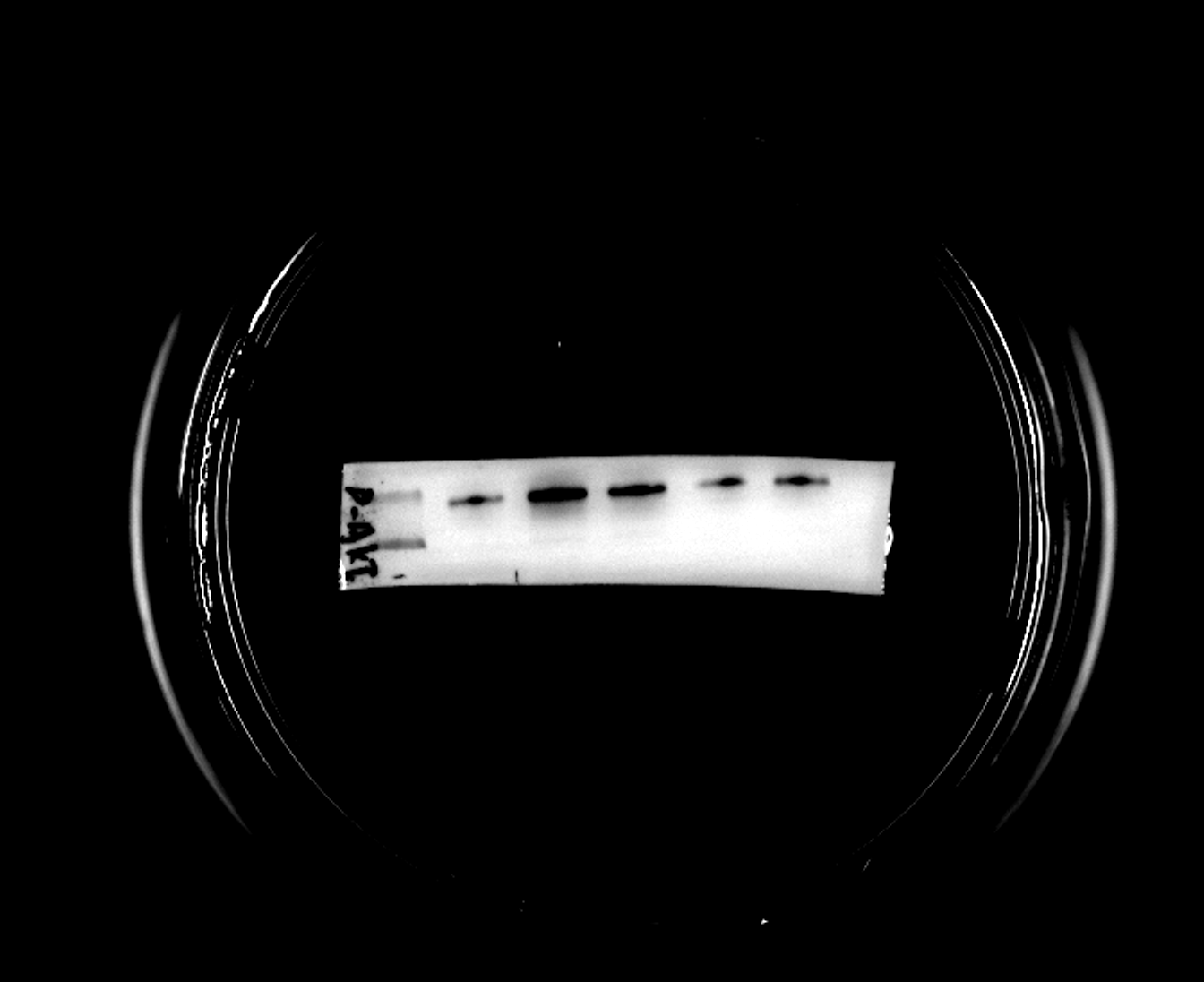

Supplement: Supplementary file 2 [file DataSheet1.ZIP › WB-rawdata/FIG4A/PAKT-2.Tif]

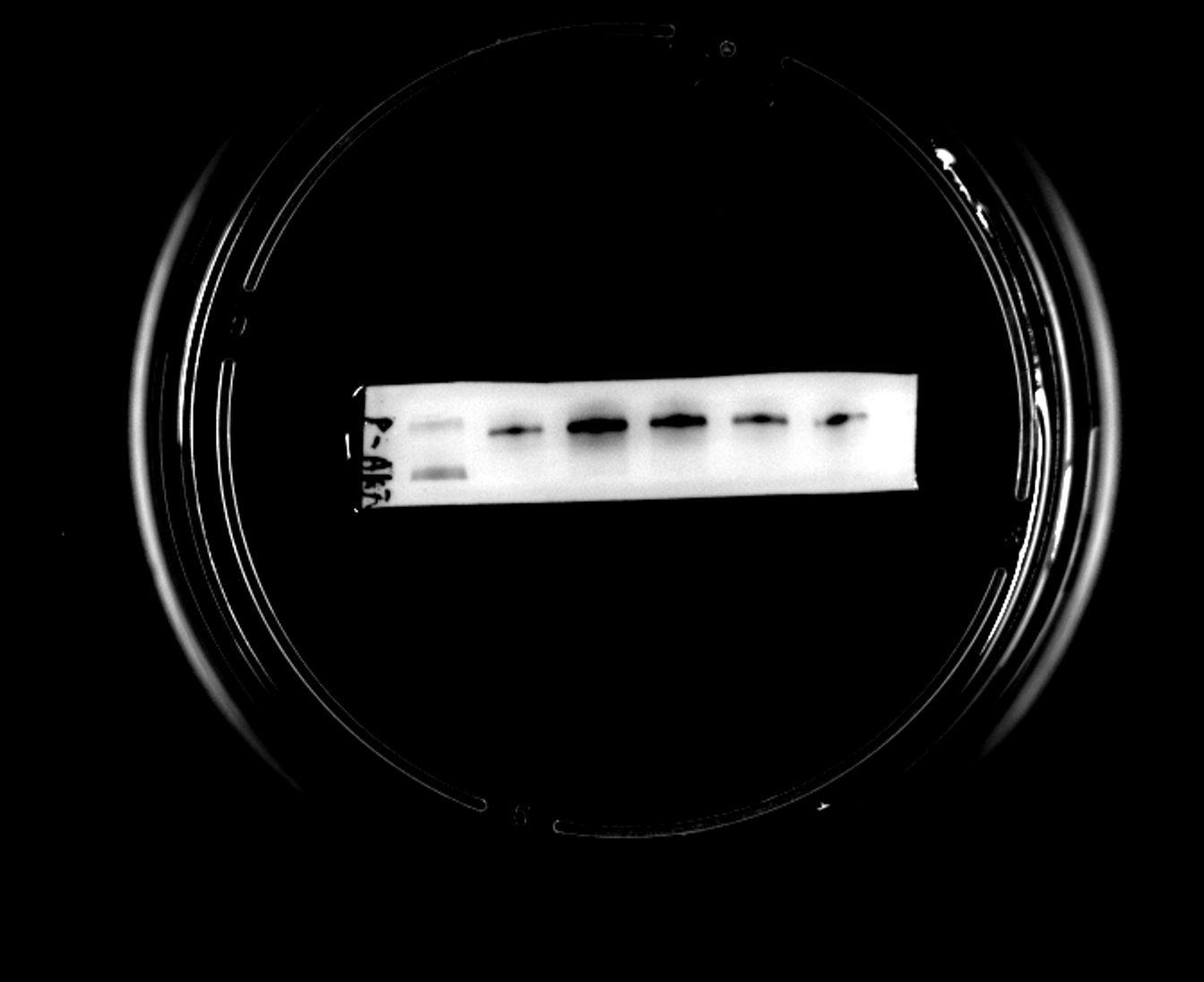

Supplement: Supplementary file 2 [file DataSheet1.ZIP › WB-rawdata/FIG4A/PAKT-1.Tif]

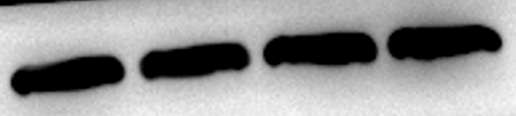

Supplement: Supplementary file 2 [file DataSheet1.ZIP › WB-rawdata/FIG2-C/actin.png]

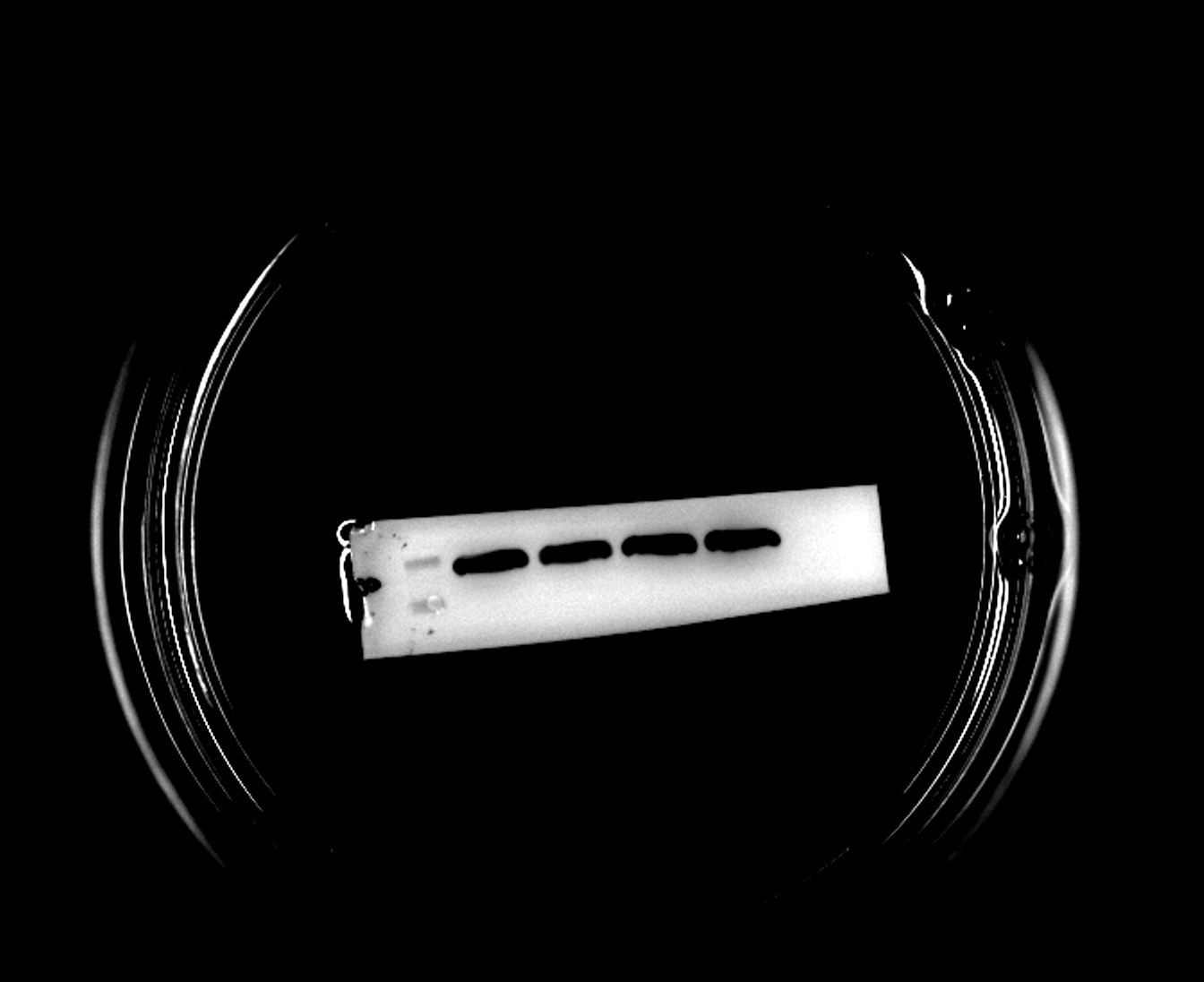

Supplement: Supplementary file 2 [file DataSheet1.ZIP › WB-rawdata/FIG2-C/Actin-1.Tif]

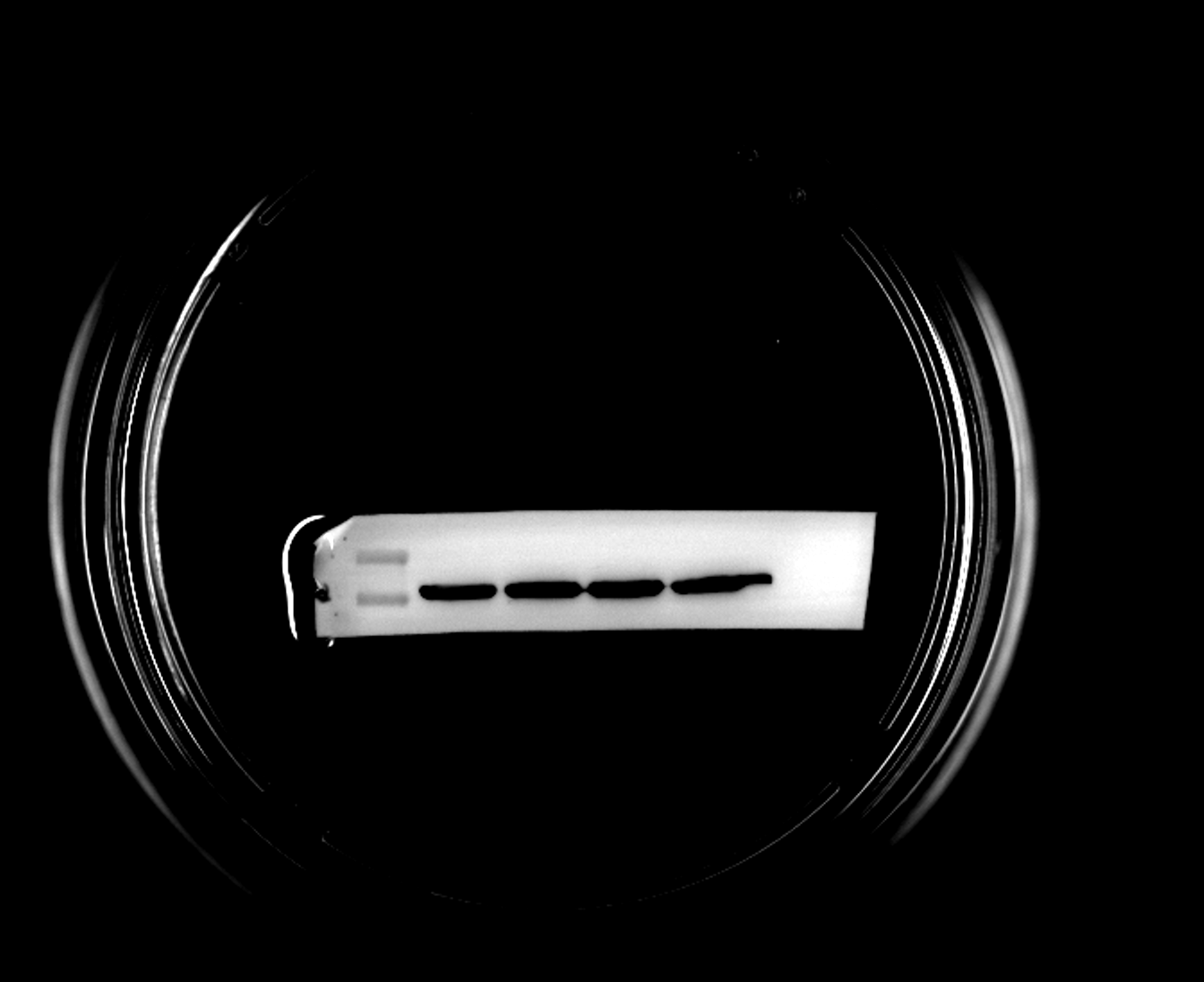

Supplement: Supplementary file 2 [file DataSheet1.ZIP › WB-rawdata/FIG2-C/Actin-3.Tif]

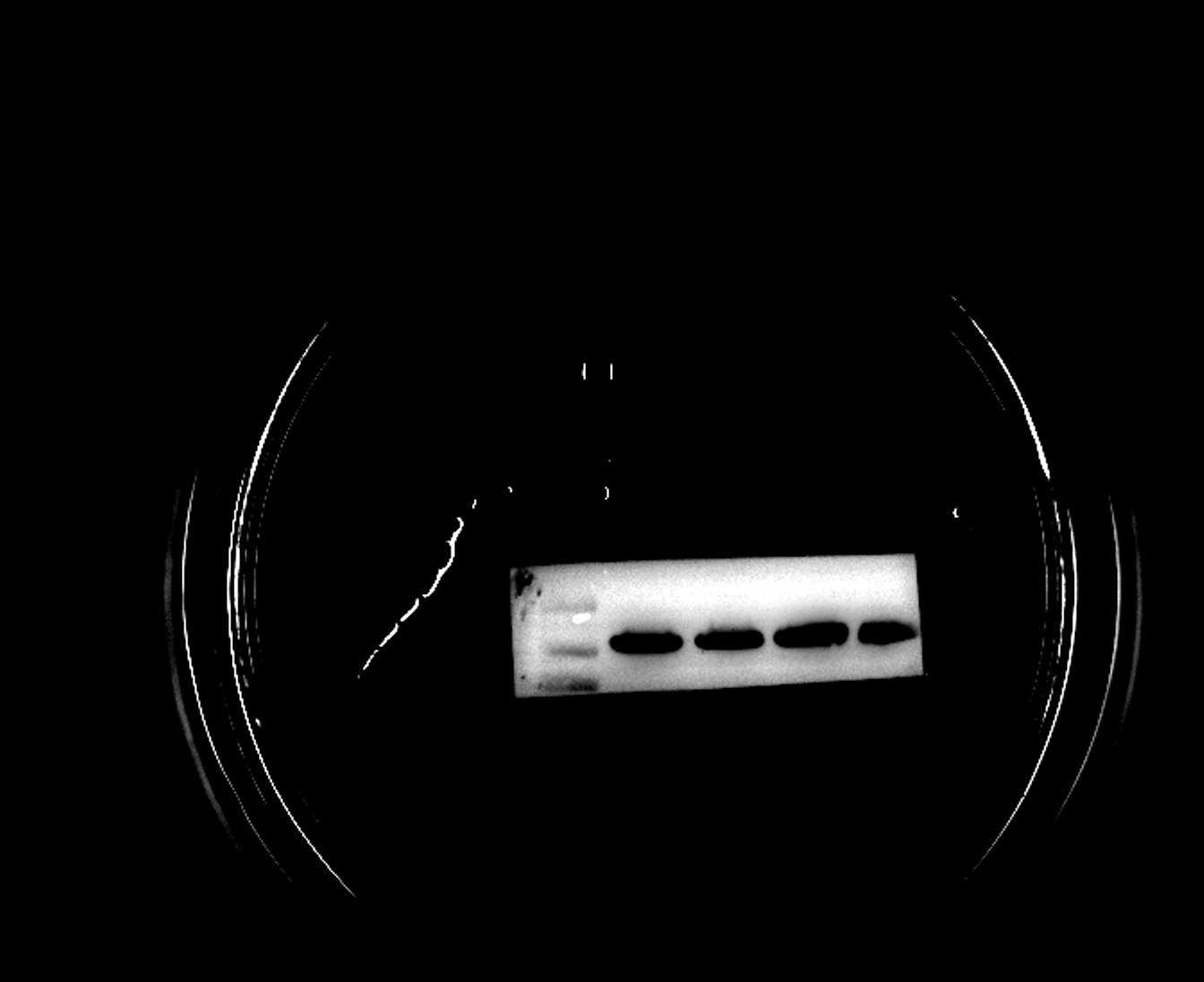

Supplement: Supplementary file 2 [file DataSheet1.ZIP › WB-rawdata/FIG2-C/ACTIN-2.Tif]

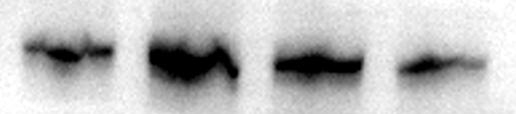

Supplement: Supplementary file 2 [file DataSheet1.ZIP › WB-rawdata/FIG2-C/runx2.png]

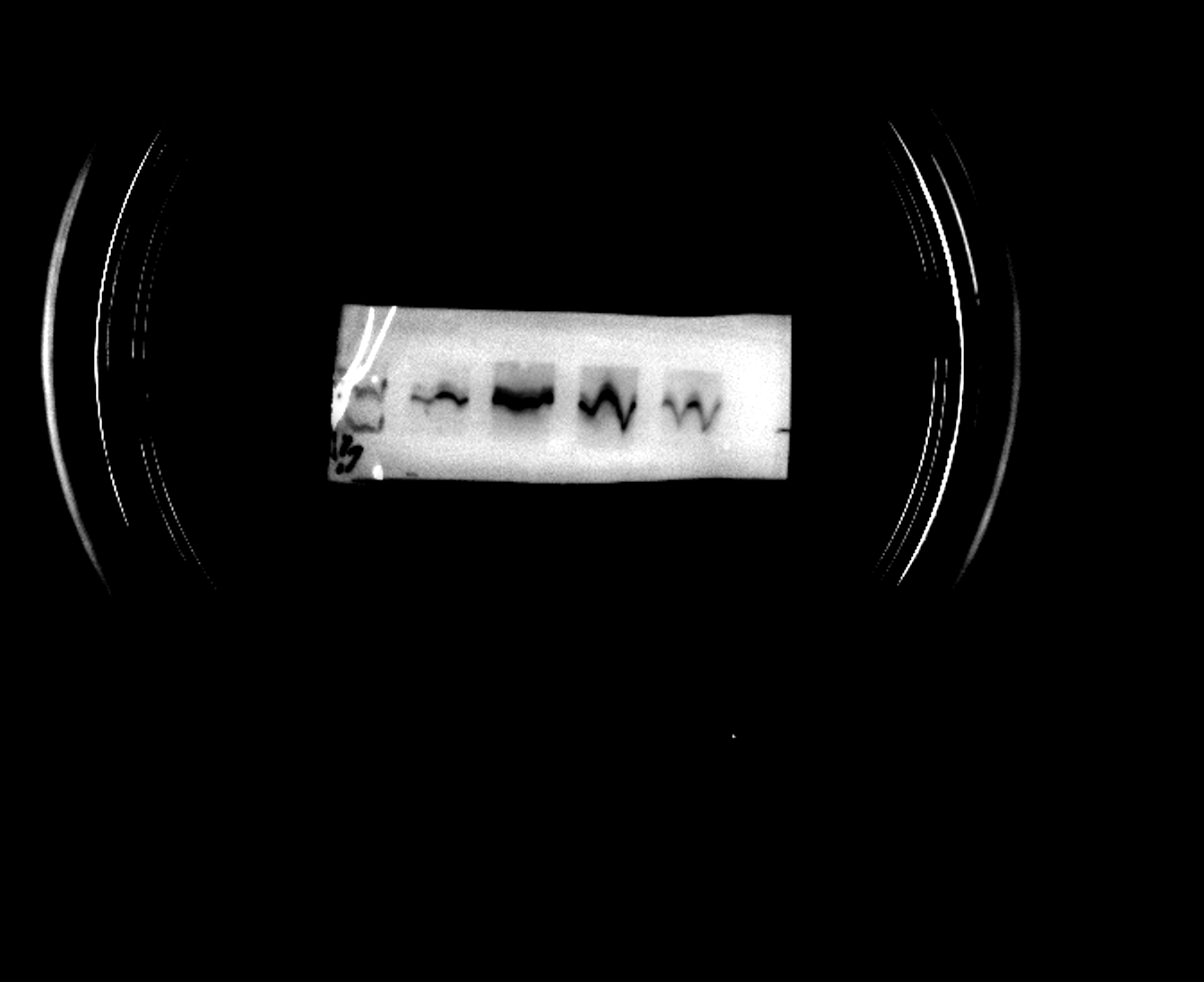

Supplement: Supplementary file 2 [file DataSheet1.ZIP › WB-rawdata/FIG2-C/RUNX2-3.Tif]

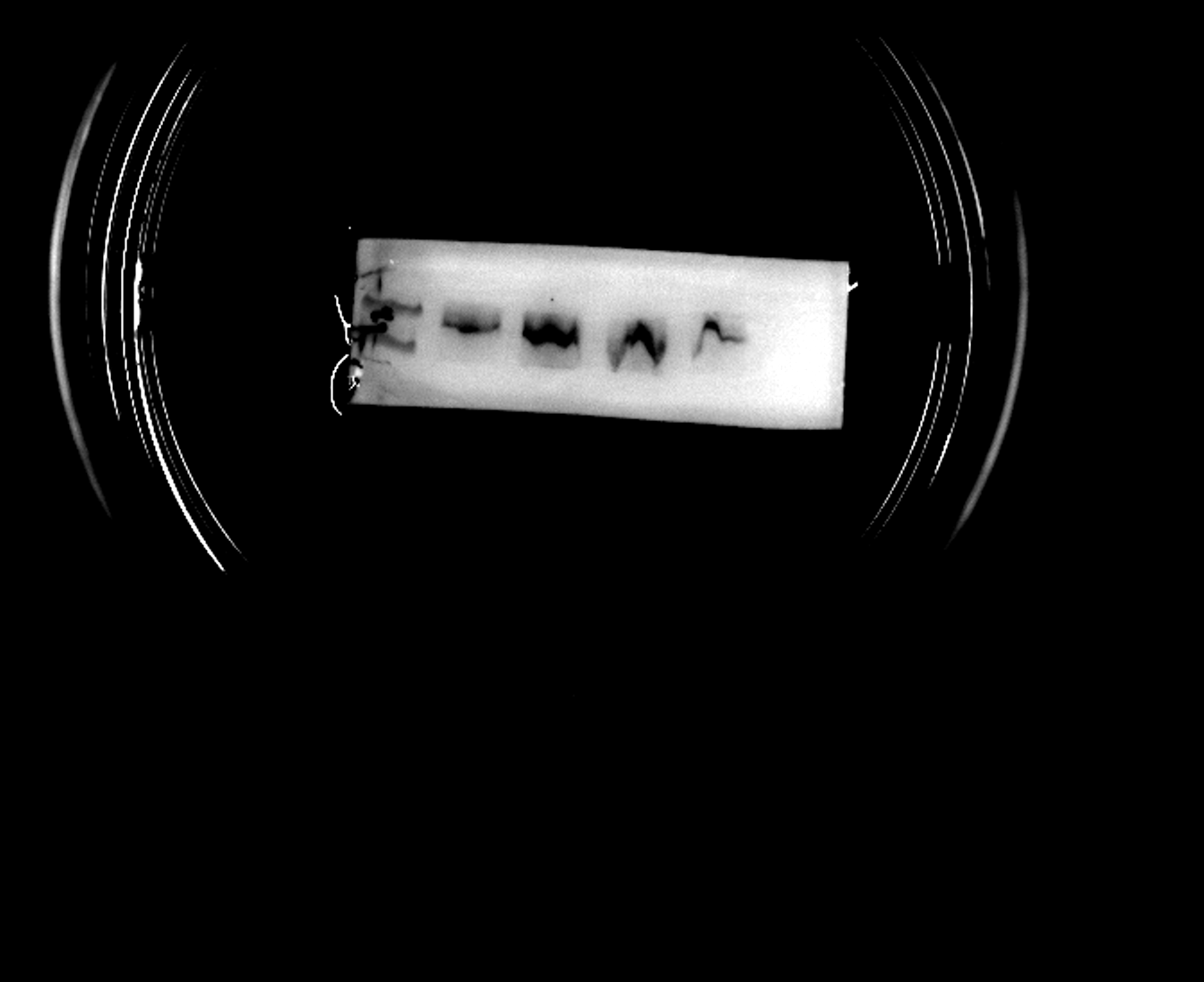

Supplement: Supplementary file 2 [file DataSheet1.ZIP › WB-rawdata/FIG2-C/RUNX2-2.Tif]

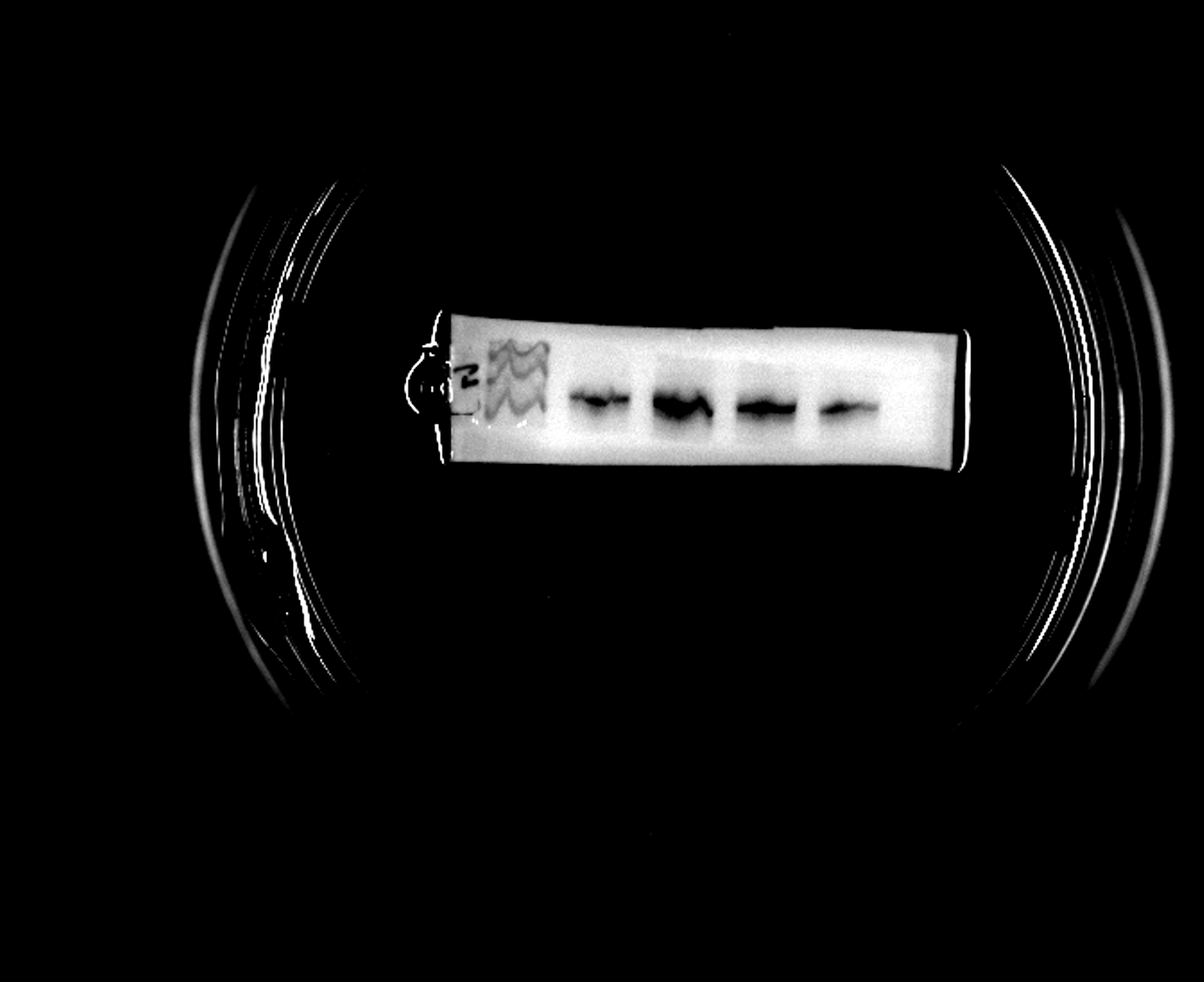

Supplement: Supplementary file 2 [file DataSheet1.ZIP › WB-rawdata/FIG2-C/RUNX2-1.Tif]

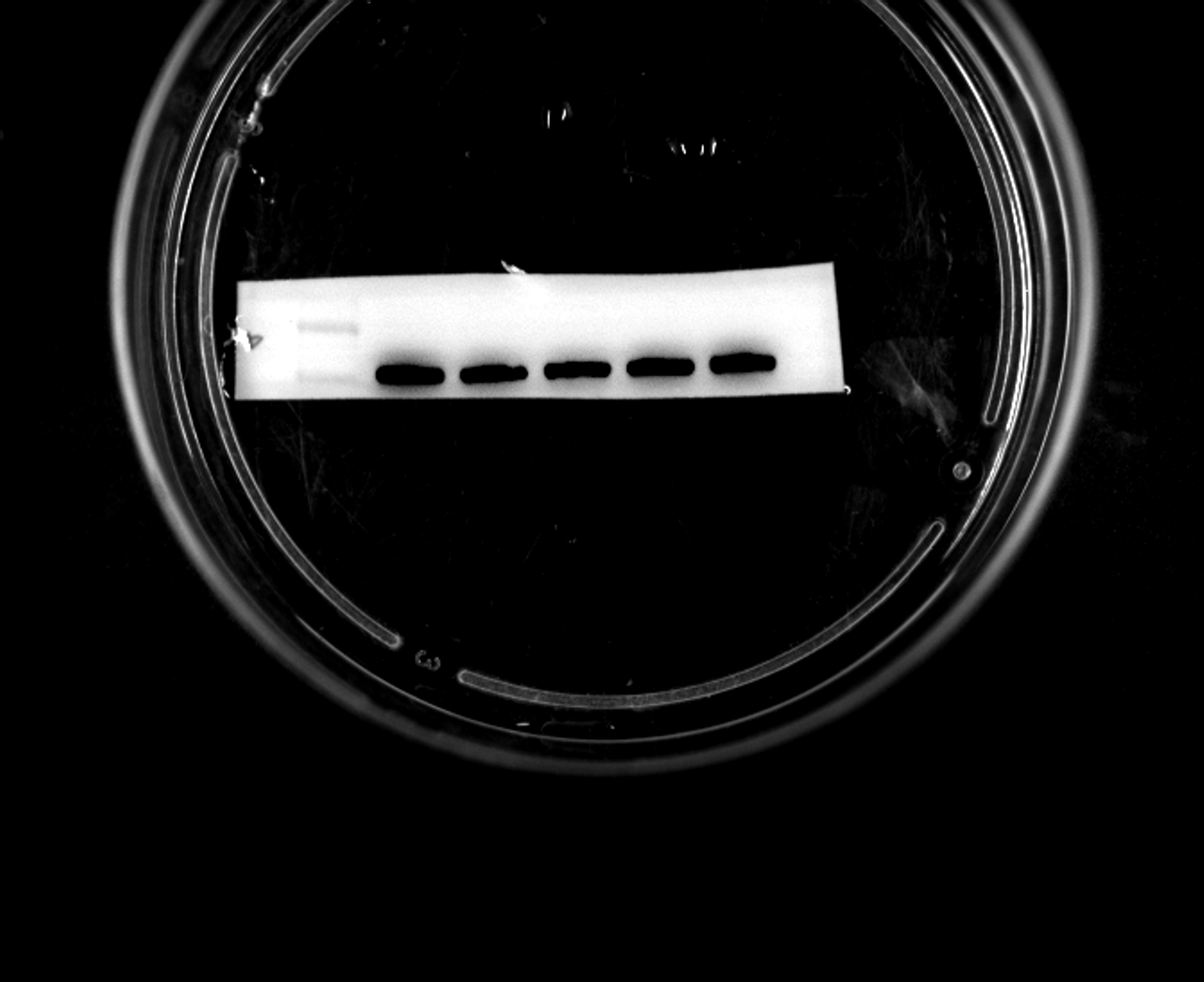

Supplement: Supplementary file 2 [file DataSheet1.ZIP › WB-rawdata/FIG4C/actin-1-merge.Tif]

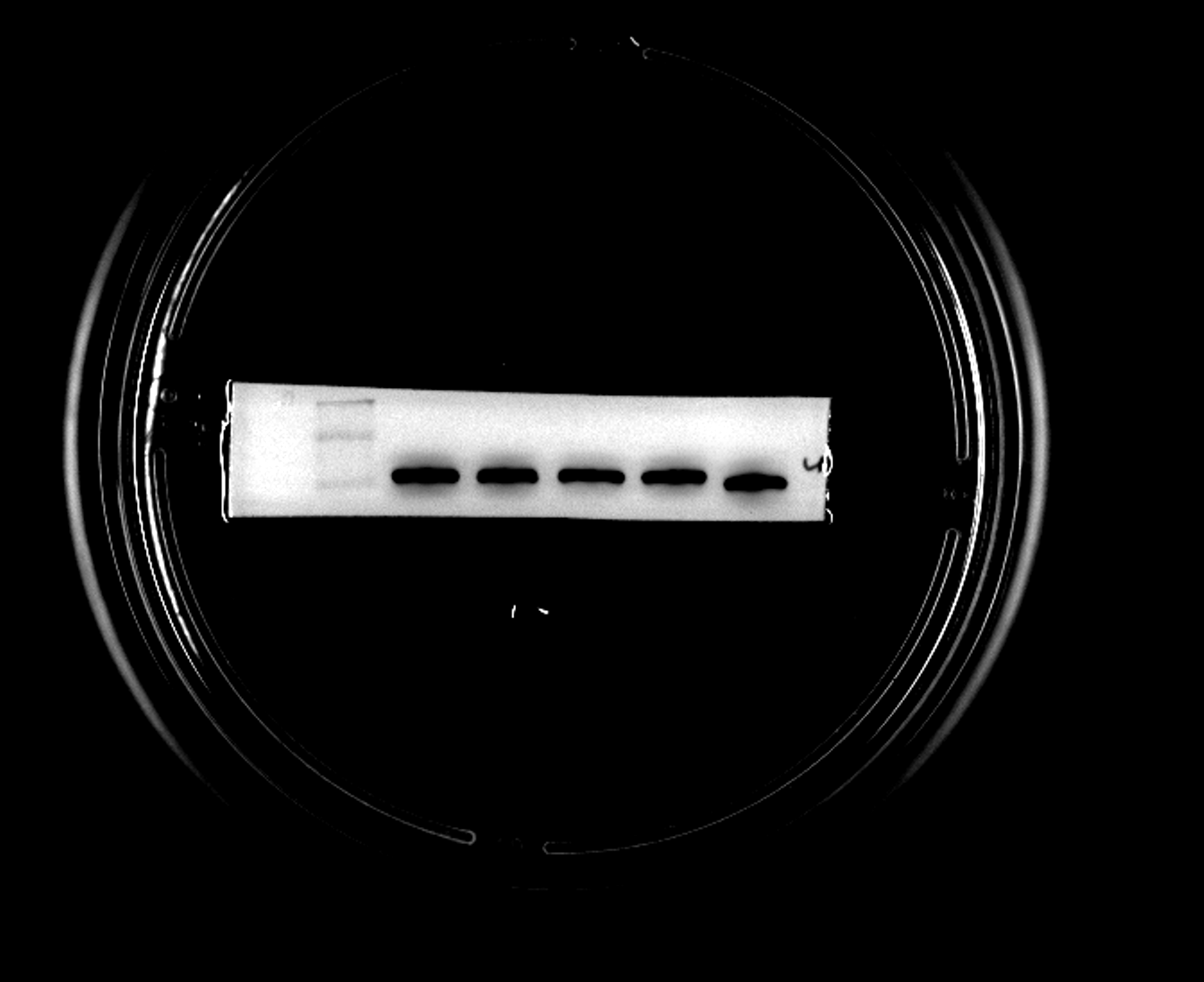

Supplement: Supplementary file 2 [file DataSheet1.ZIP › WB-rawdata/FIG4C/actin-2-merge.Tif]

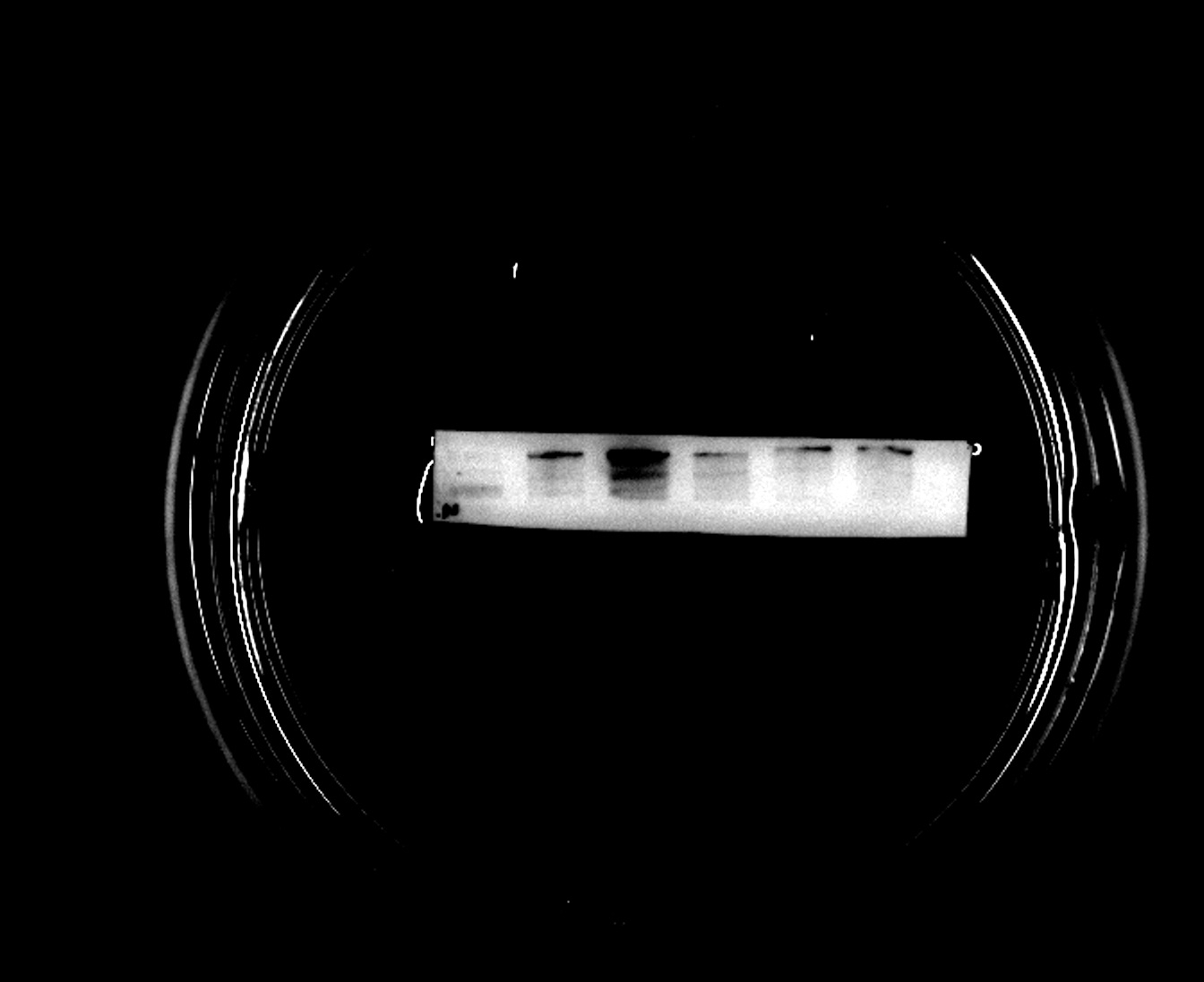

Supplement: Supplementary file 2 [file DataSheet1.ZIP › WB-rawdata/FIG4C/HIF1-╬▒-3.Tif]

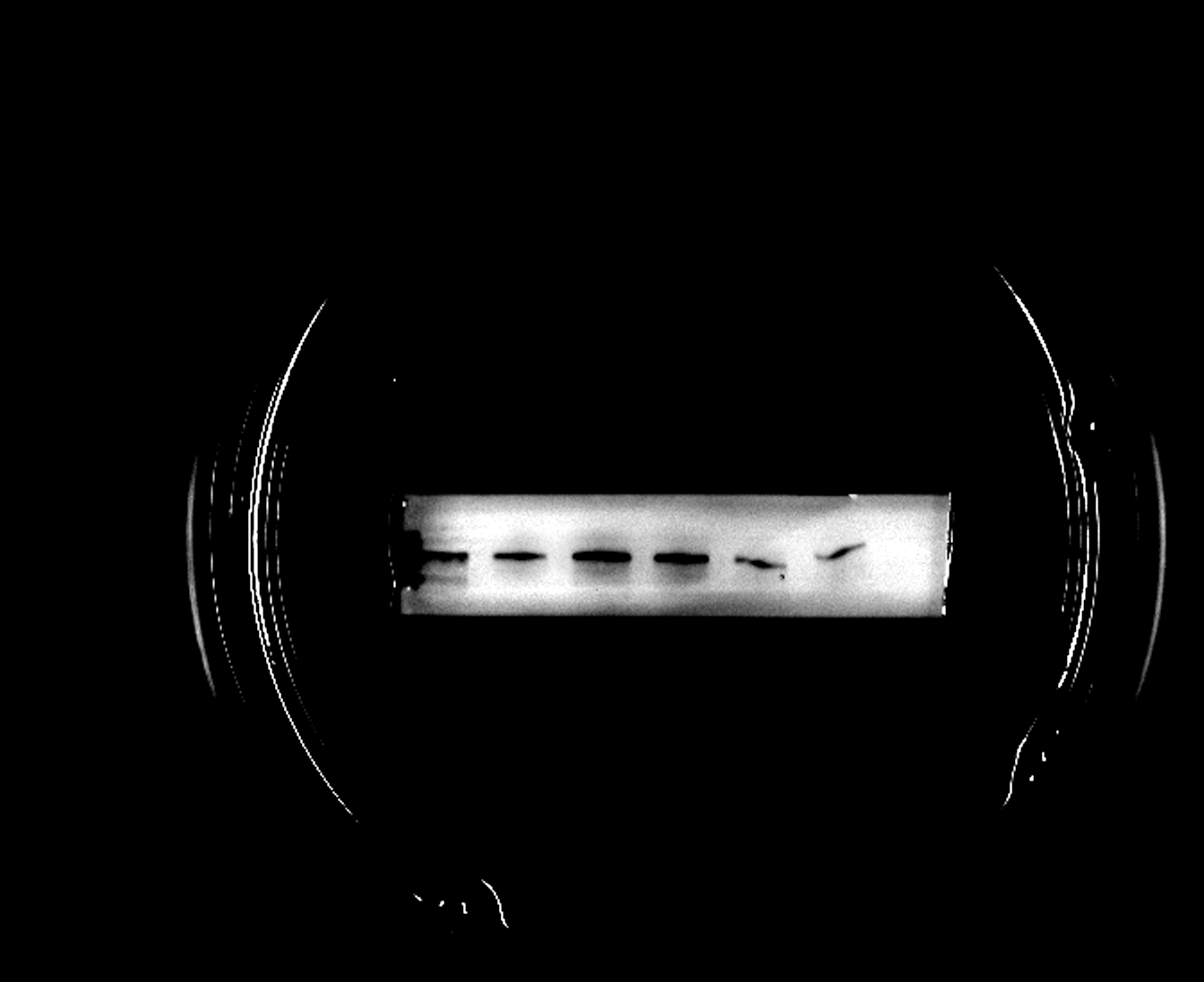

Supplement: Supplementary file 2 [file DataSheet1.ZIP › WB-rawdata/FIG4C/HIF1-╬▒-2.Tif]

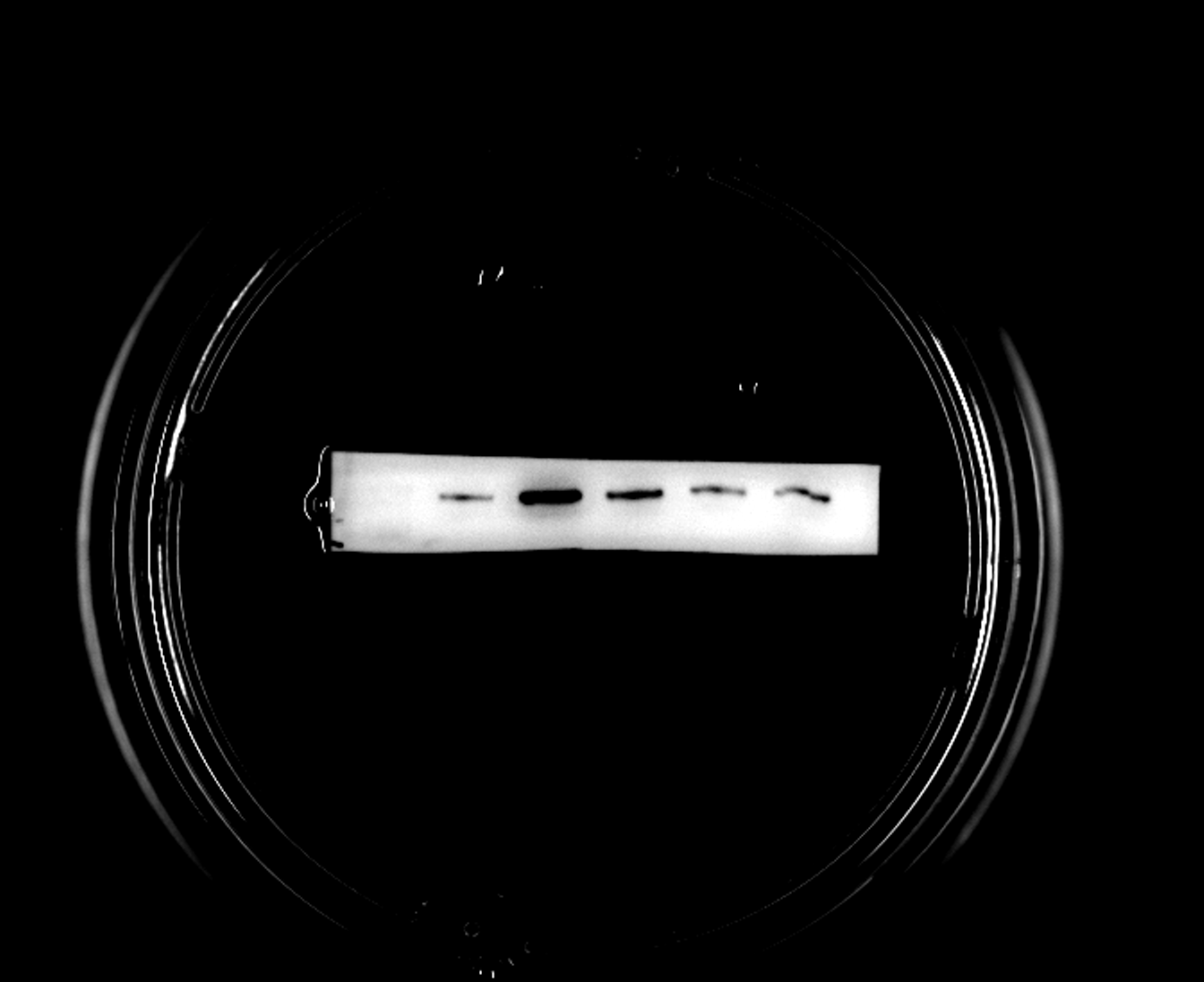

Supplement: Supplementary file 2 [file DataSheet1.ZIP › WB-rawdata/FIG4C/HIF1-╬▒-1.Tif]

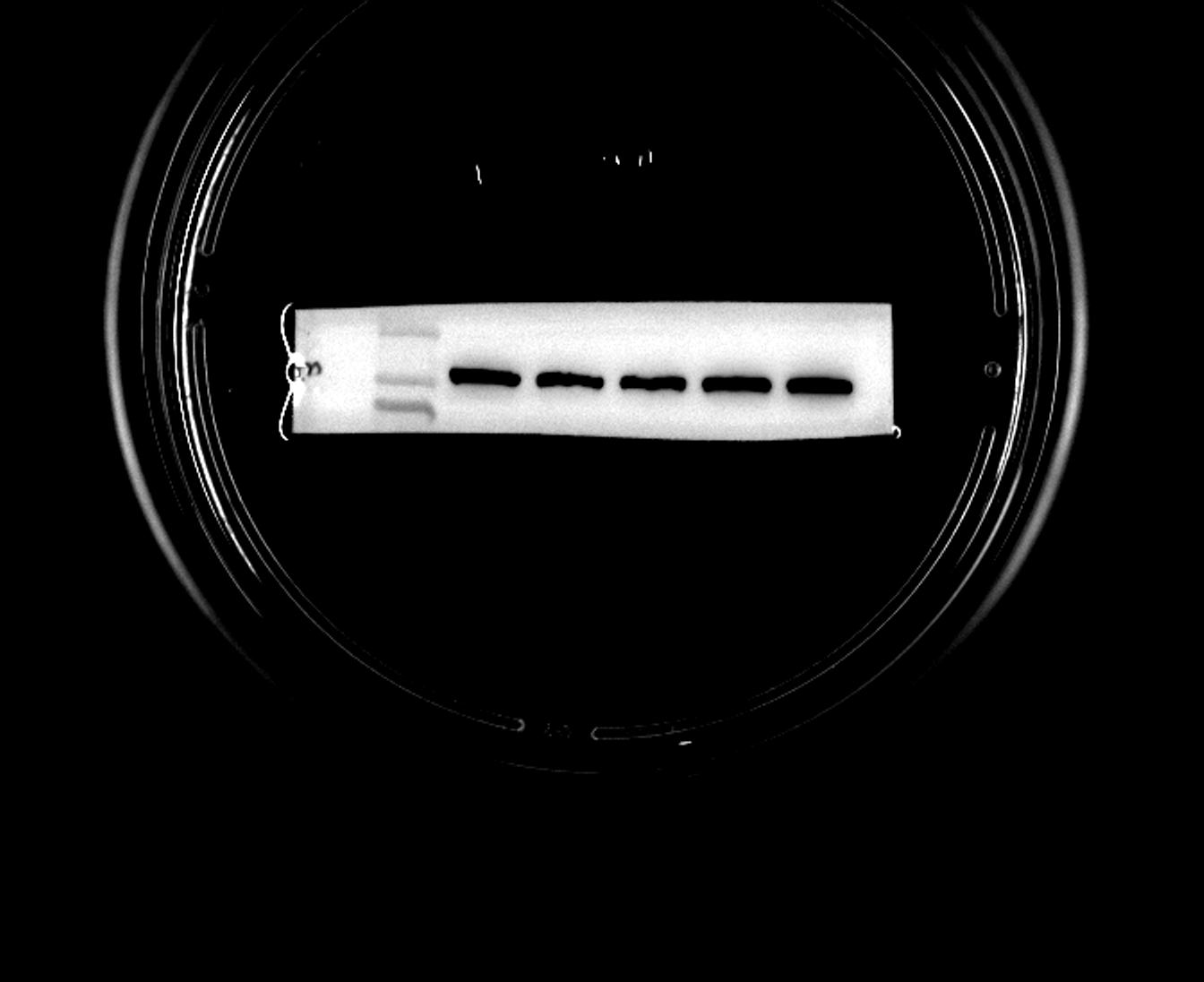

Supplement: Supplementary file 2 [file DataSheet1.ZIP › WB-rawdata/FIG4C/actin-3-merge.Tif]

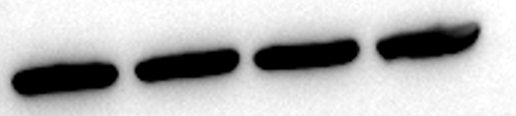

Supplement: Supplementary file 2 [file DataSheet1.ZIP › WB-rawdata/FIG2-B/ACTIN.png]

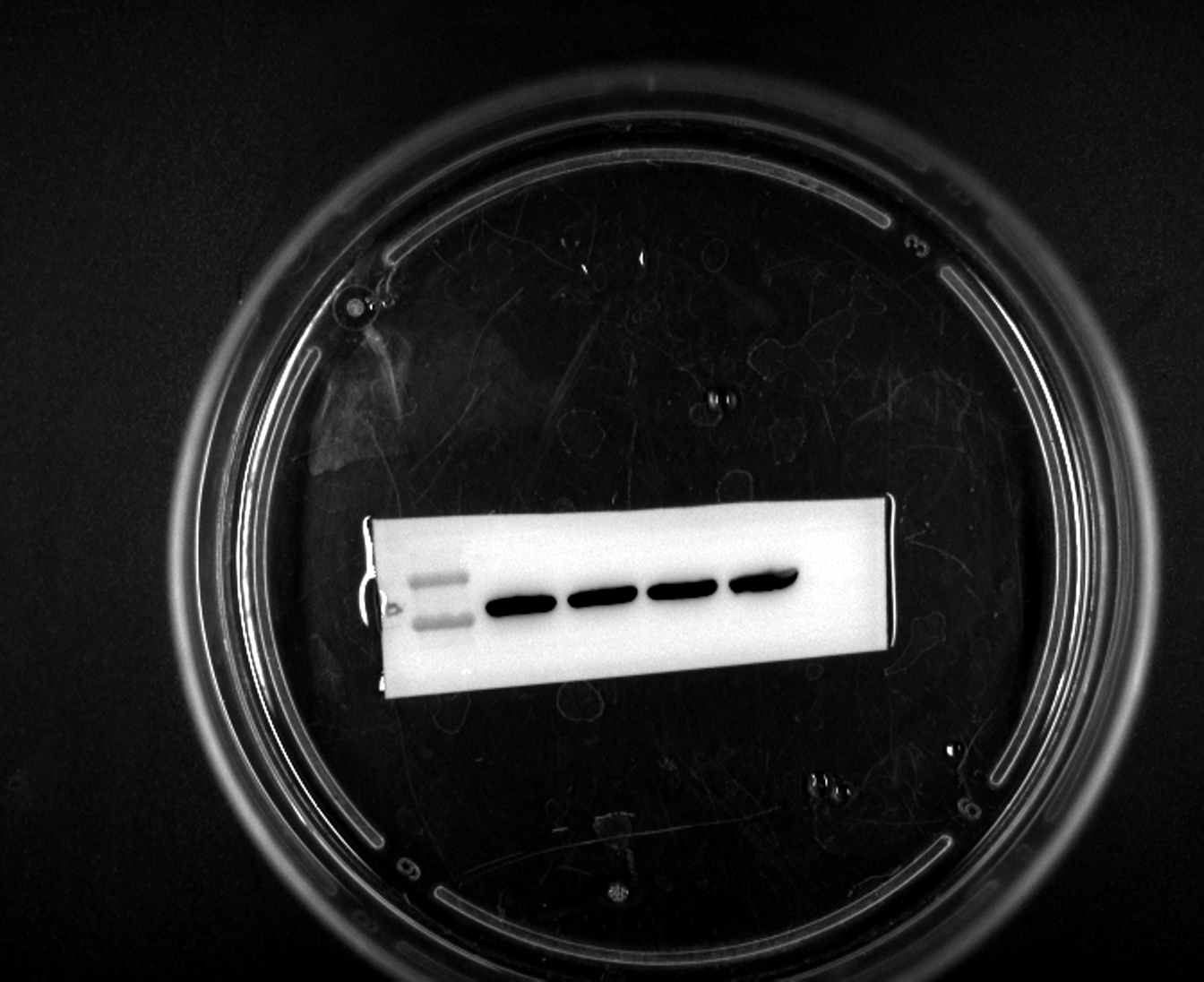

Supplement: Supplementary file 2 [file DataSheet1.ZIP › WB-rawdata/FIG2-B/ACTIN-1.Tif]

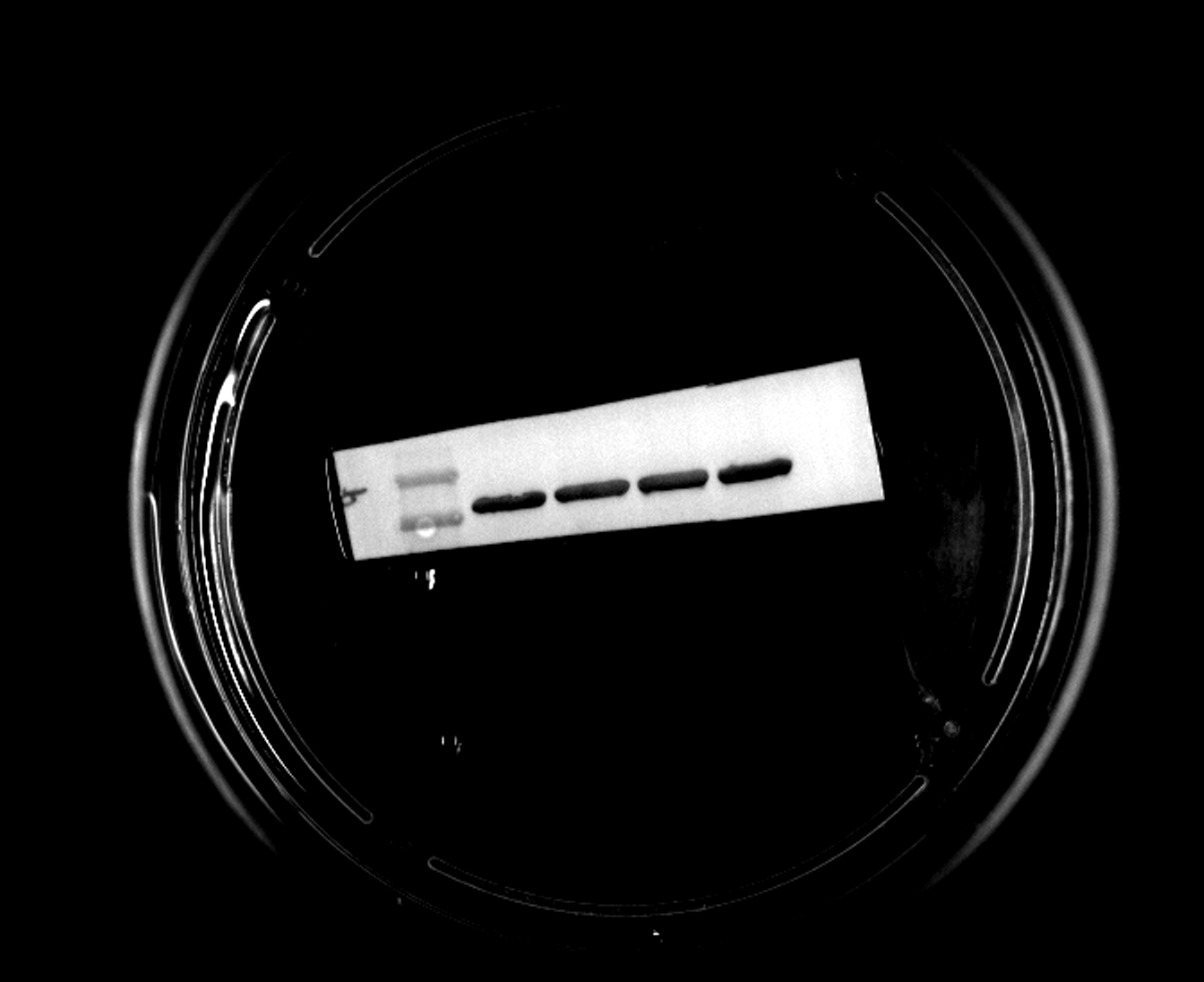

Supplement: Supplementary file 2 [file DataSheet1.ZIP › WB-rawdata/FIG2-B/ACTIN-3.Tif]

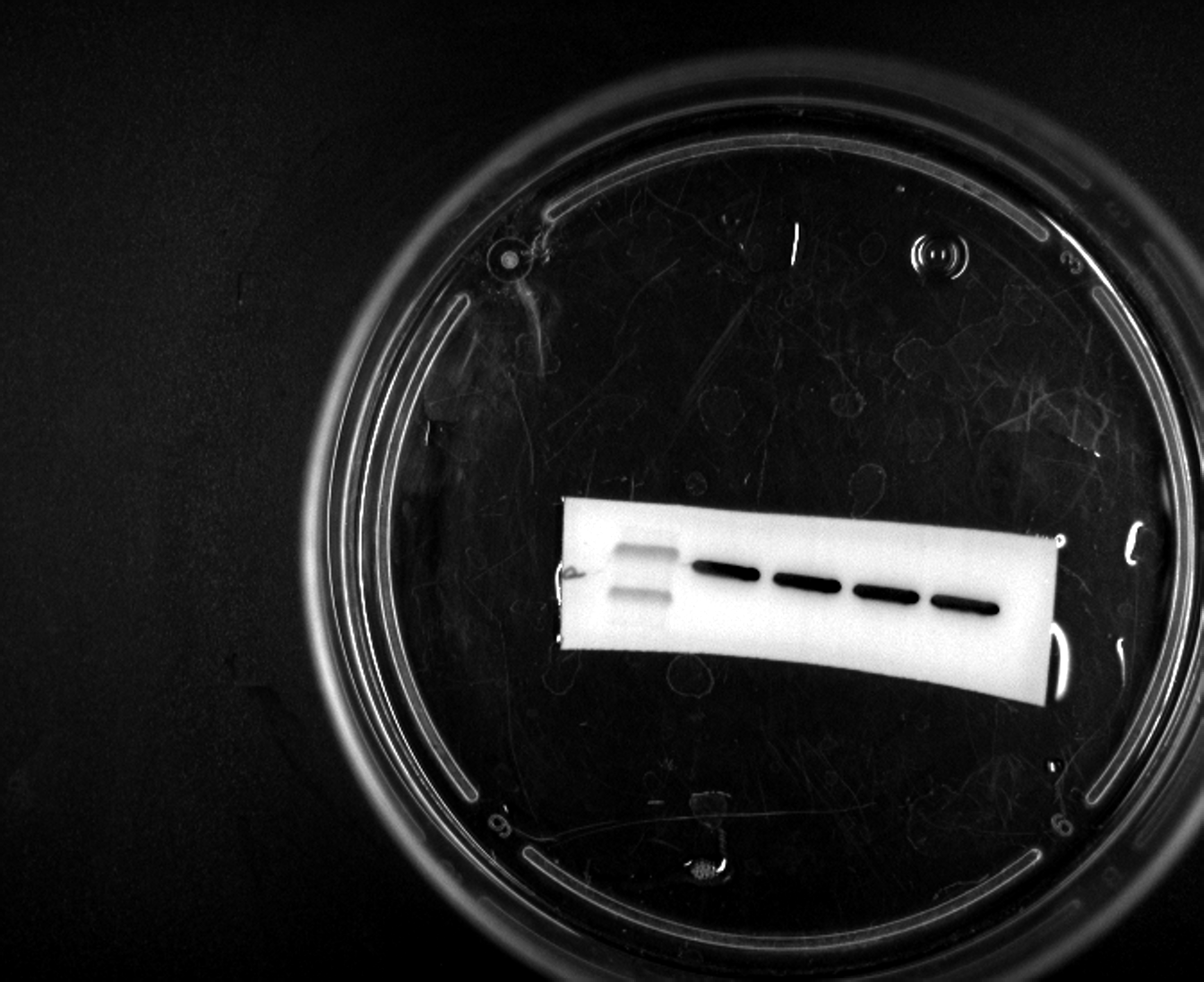

Supplement: Supplementary file 2 [file DataSheet1.ZIP › WB-rawdata/FIG2-B/ACTIN-2.Tif]

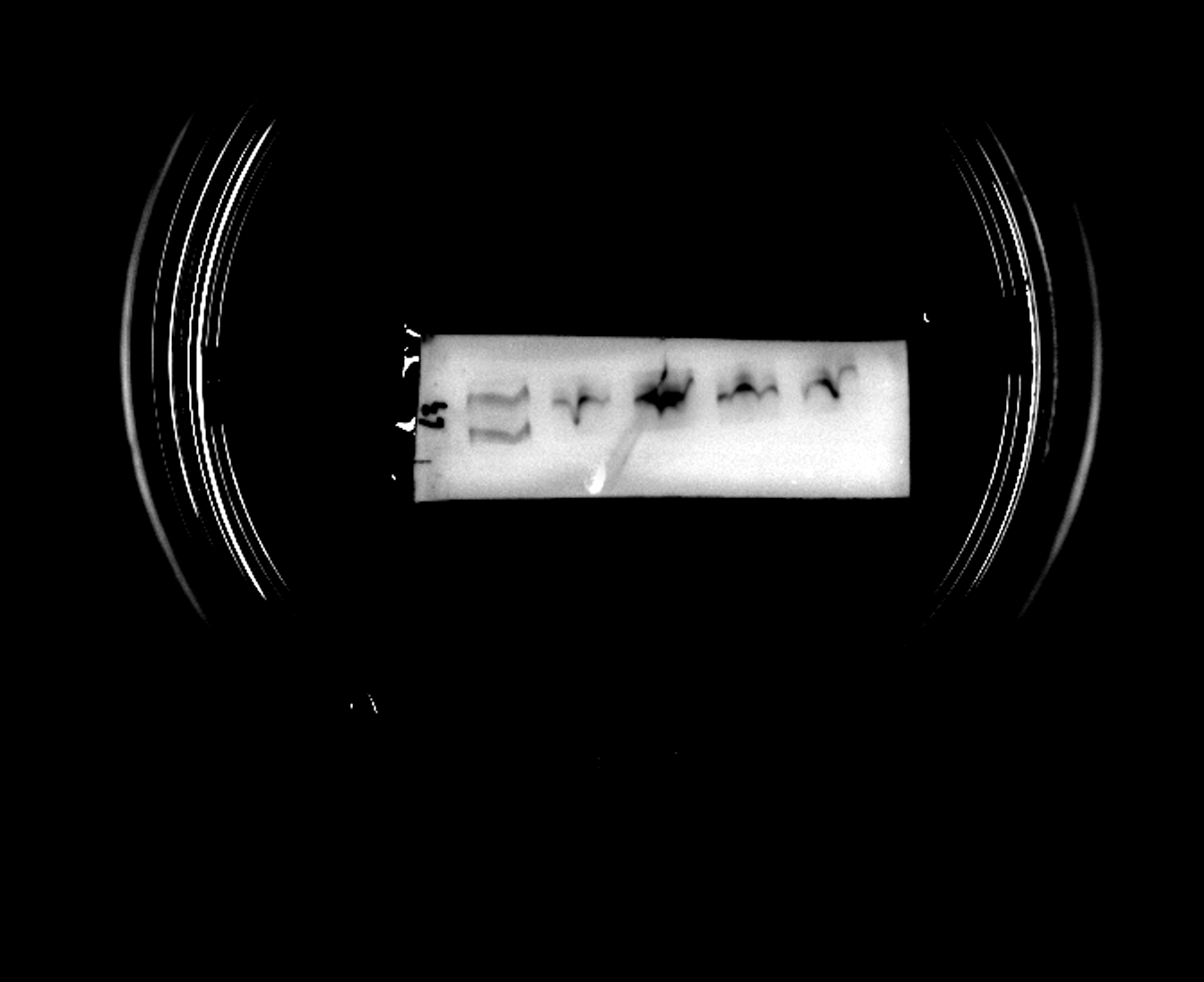

Supplement: Supplementary file 2 [file DataSheet1.ZIP › WB-rawdata/FIG2-B/ALP-2.Tif]

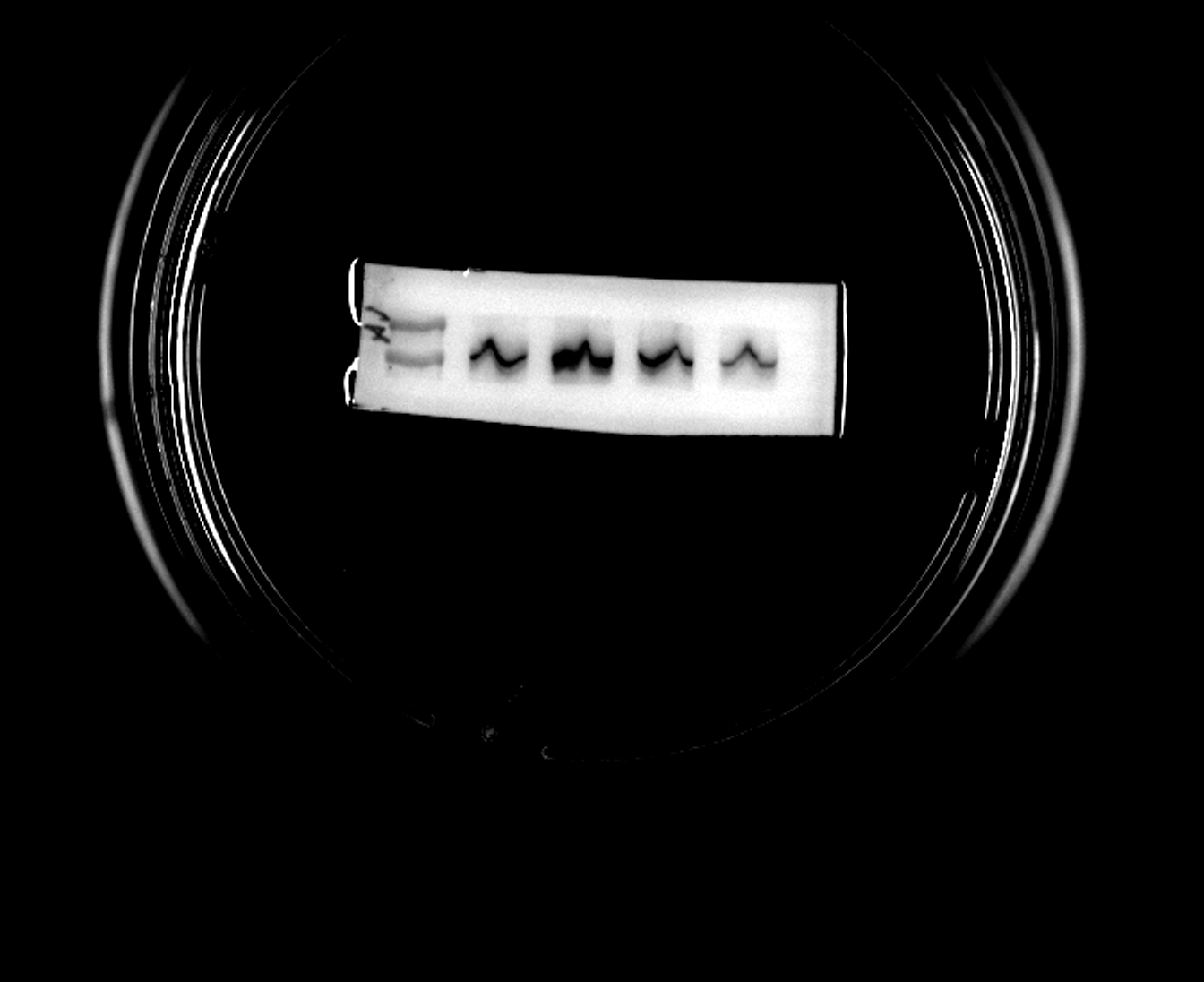

Supplement: Supplementary file 2 [file DataSheet1.ZIP › WB-rawdata/FIG2-B/ALP-3.Tif]

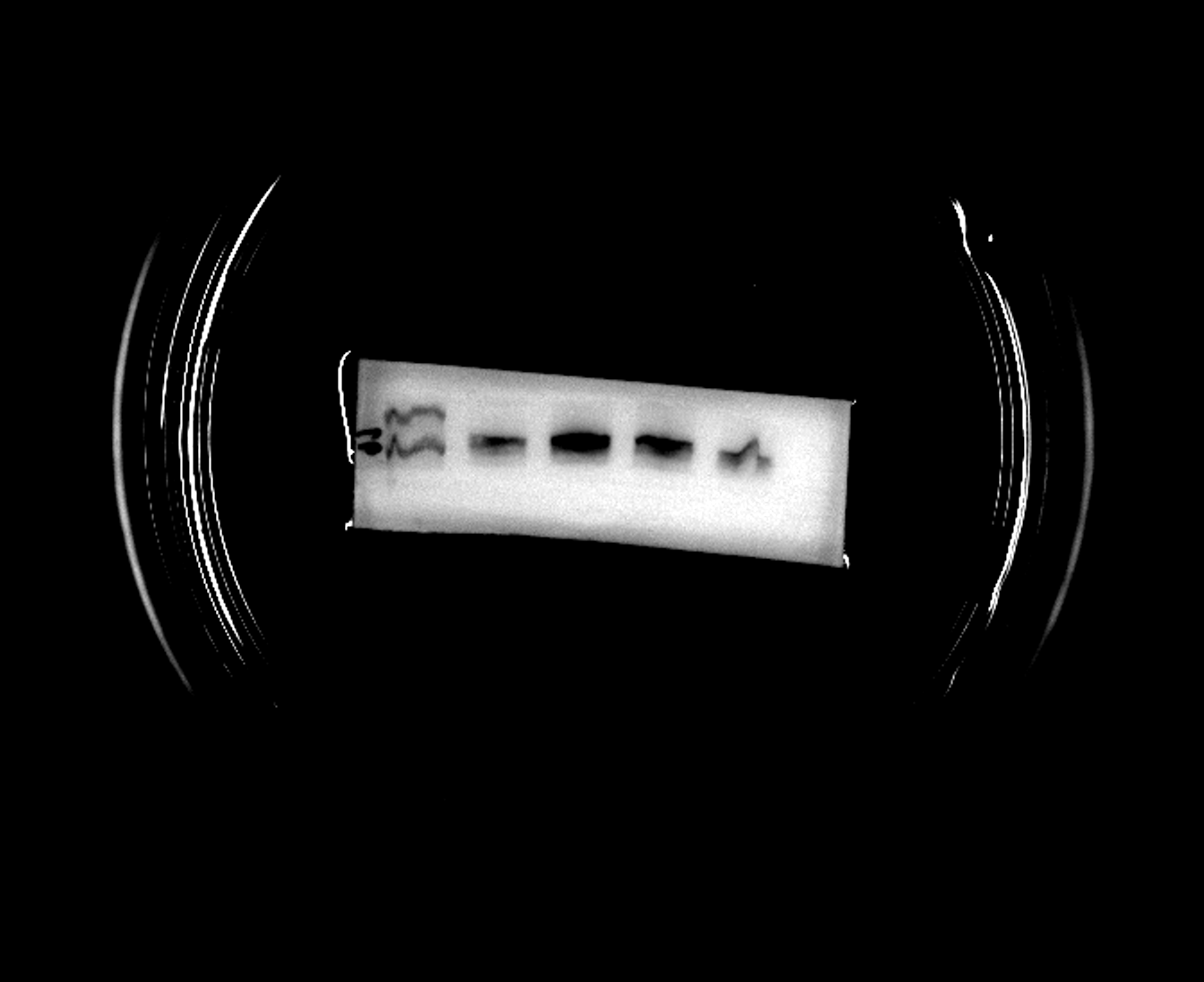

Supplement: Supplementary file 2 [file DataSheet1.ZIP › WB-rawdata/FIG2-B/ALP-1.Tif]

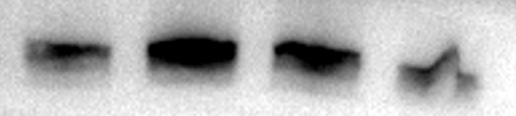

Supplement: Supplementary file 2 [file DataSheet1.ZIP › WB-rawdata/FIG2-B/ALP.png]
